# Methylomic trajectories across human fetal brain development

**DOI:** 10.1101/gr.180273.114

**Published:** 2015-03

**Authors:** Helen Spiers, Eilis Hannon, Leonard C. Schalkwyk, Rebecca Smith, Chloe C.Y. Wong, Michael C. O’Donovan, Nicholas J. Bray, Jonathan Mill

**Affiliations:** 1 Institute of Psychiatry, Psychology & Neuroscience, King’s College London, London SE5 8AF, United Kingdom;; 2 University of Exeter Medical School, University of Exeter, Exeter EX2 5DW, United Kingdom;; 3 School of Biological Sciences, University of Essex, Colchester CO4 3SQ, United Kingdom;; 4 MRC Centre for Neuropsychiatric Genetics and Genomics, Cardiff University School of Medicine, Cardiff CF24 4HQ, United Kingdom

## Abstract

Epigenetic processes play a key role in orchestrating transcriptional regulation during development. The importance of DNA methylation in fetal brain development is highlighted by the dynamic expression of de novo DNA methyltransferases during the perinatal period and neurodevelopmental deficits associated with mutations in the methyl-CpG binding protein 2 (*MECP2*) gene. However, our knowledge about the temporal changes to the epigenome during fetal brain development has, to date, been limited. We quantified genome-wide patterns of DNA methylation at ∼400,000 sites in 179 human fetal brain samples (100 male, 79 female) spanning 23 to 184 d post-conception. We identified highly significant changes in DNA methylation across fetal brain development at >7% of sites, with an enrichment of loci becoming hypomethylated with fetal age. Sites associated with developmental changes in DNA methylation during fetal brain development were significantly underrepresented in promoter regulatory regions but significantly overrepresented in regions flanking CpG islands (shores and shelves) and gene bodies. Highly significant differences in DNA methylation were observed between males and females at a number of autosomal sites, with a small number of regions showing sex-specific DNA methylation trajectories across brain development. Weighted gene comethylation network analysis (WGCNA) revealed discrete modules of comethylated loci associated with fetal age that are significantly enriched for genes involved in neurodevelopmental processes. This is, to our knowledge, the most extensive study of DNA methylation across human fetal brain development to date, confirming the prenatal period as a time of considerable epigenomic plasticity.

Human brain development is an intricate process involving the dynamic orchestration of gene expression. Prenatal transcriptional changes in the brain occur more rapidly than at any other stage of life (Johnson et al. 2009; Colantuoni et al. 2011; Kang et al. 2011). The precise temporal regulation of transcriptional processes is necessary for the correct development of structural and functional complexity in the brain. Although cell-specific and temporally appropriate gene expression is primarily controlled through the direct action of transcription factors, there is growing recognition of the role of epigenetic mechanisms in the dynamic regulation of gene function during cellular development and differentiation (Henikoff and Matzke 1997; Jaenisch and Bird 2003; Hirabayashi and Gotoh 2010).

DNA methylation is the most extensively studied epigenetic modification. It is known to play a role in many important genomic regulatory processes, including X Chromosome inactivation, genomic imprinting and the repression of tumor suppressor genes in cancer. DNA methylation refers to the addition of a single methyl group to carbon five of the cytosine pyrimidine ring, typically in the context of palindromic 5′-CpG-3′ dinucleotides, of which there are ∼28 million in the haploid human genome, and more rarely in a non-CpG context. The covalently attached methyl groups project into the major groove of DNA where they can inhibit transcription by blocking the binding of transcription factors and by recruiting methyl-CpG binding proteins such as MECP2 which remodel chromatin into a condensed heterochromatic state. Recent work has revealed a more nuanced relationship between DNA methylation and transcription that is dependent on genomic and cellular context (Jones 2012); although DNA methylation at promoter regulatory regions is typically associated with repressed expression, DNA methylation in the gene body is often positively correlated with expression (Ball et al. 2009; Maunakea et al. 2010) and is thought to play a role in alternative splicing (Maunakea et al. 2013).

The establishment and maintenance of cell-specific DNA methylation patterns is crucial for normal mammalian development (Reik 2007; Geiman and Muegge 2010; Smith and Meissner 2013; Ziller et al. 2013). Recent evidence strongly implicates a role for dynamic epigenetic processes in the regulation of transcriptional plasticity in the developing brain (Numata et al. 2012; Lister et al. 2013). A critical role for DNA methylation in neurodevelopment is supported by the dynamic expression of the de novo DNA methyltransferases *DNMT3A* and *DNMT3B* during prenatal brain development (Feng et al. 2005), and by the occurrence of neurodevelopmental deficits in humans as a consequence of mutations in the methyl-CpG binding protein 2 (*MECP2*) gene, which interacts with methylated DNA to control neuronal gene expression (Guy et al. 2011; Jakovcevski and Akbarian 2012). Furthermore, the dynamic regulation of DNA methylation is known to influence key neurobiological and cognitive functions in the brain across the life course, including neuronal plasticity (Borrelli et al. 2008; Ma et al. 2010; Guo et al. 2011), memory formation and maintenance (Day and Sweatt 2010; Zovkic et al. 2013), and circadian processes (Azzi et al. 2014). Knowledge about the specific temporal methylomic changes occurring during human fetal brain development has, however, been limited due to a lack of tissue samples, with previous studies focusing on a small number of samples obtained from a narrow range of fetal ages (Numata et al. 2012; Lister et al. 2013).

Here, we describe an analysis of neurodevelopmental trajectories in DNA methylation in human fetal brain samples, identifying changes to the epigenome during development across ∼400,000 sites.

## Results

### Methodological overview

**Figure 1.**
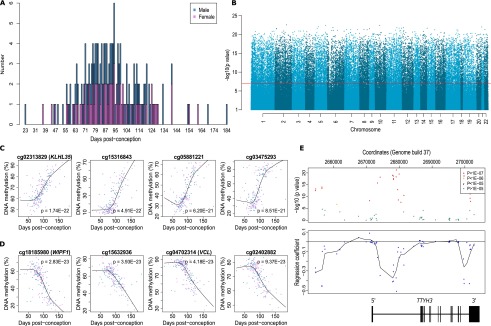
DNA methylation changes during human brain development are widespread across the genome. (*A*) Overview of the 179 human fetal brain samples (100 male, 79 female) spanning 23 to 184 DPC profiled in this study. (*B*) Manhattan plot showing the widespread distribution of Bonferroni-significant fetal brain dDMPs (*P*-value corresponds to association with age). (*C*) The four top-ranked dDMPs showing increased DNA methylation with fetal age (hypermethylated dDMPs). DNA methylation (%) is plotted against DPC. Females are shown in pink, males in blue. (*D*) The four top-ranked dDMPs showing decreased DNA methylation with fetal age (hypomethylated dDMPs). DNA methylation (%) is plotted against DPC. Females are shown in pink, males in blue. (*E*) Many loci are characterized by regions of extended differential DNA methylation associated with fetal brain development. Shown is the *TTYH3* gene that contains three discrete differentially methylated regions (DMRs), which become hypomethylated during fetal brain development. The *top* panel depicts the association statistic for individual probes, with color corresponding to significance. The *bottom* panel depicts the regression coefficient between DNA methylation and brain development for individual probes, with a line of best-fit highlighting three domains characterized by hypomethylation across brain development.

We assessed genome-wide patterns of DNA methylation in 179 human fetal brain samples (100 male, 79 female) spanning 23 to 184 d post-conception (DPC) (Fig. 1A). Fetal brain tissue was acquired frozen from the Human Developmental Biology Resource (HDBR) (http://www.hdbr.org) and MRC Brain Banks network (http://www.mrc.ac.uk/research/facilities/brain-banks/access-for-research) under strict ethical regulations and used to isolate genomic DNA. DNA methylation was quantified using the Illumina Infinium HumanMethylation450 BeadChip, with preprocessing, normalization, and stringent quality control undertaken as previously described (Pidsley et al. 2013). Our analyses focused on identifying DNA methylation changes associated with brain development, and whether these were enriched in certain genomic regions and features or differed between males and females. We subsequently employed systems-level network-based approaches to identify modules of highly comethylated loci and their relationship to fetal brain development. See the Methods section for an in depth description of the samples and analytical approaches used in this study.

### Human fetal brain development is characterized by widespread changes in DNA methylation

A combined analysis of all 408,608 probes on the Illumina 450K array passing stringent quality control (QC) metrics showed that global levels of DNA methylation do not change significantly over the course of human fetal brain development (r = −0.02, *P* = 0.76) (Supplemental Fig. 1). In contrast, DNA methylation at individual autosomal sites was highly variable across neurodevelopment, with levels at 28,718 sites (7.19% of the 399,364 autosomal probes assessed) differing according to fetal age at a significance level surpassing Bonferroni correction for all tested probes (*P* < 1.25 × 10^−7^) (Fig. 1B). We refer to these as developmentally differentially methylated positions (dDMPs). The 20 top autosomal dDMPs (ranked by *P*-value for age-association), characterized by a positive correlation between DNA methylation and fetal age (henceforth referred to as hypermethylated dDMPs), or a negative correlation (henceforth referred to as hypomethylated dDMPs), are listed in Table 1. A complete list of all 28,718 significant dDMPs is available from our laboratory website (http://epigenetics.iop.kcl.ac.uk/fetalbrain/dDMPs.xls) and in Supplemental File 1. Although the distribution of dDMPs is relatively consistent across most autosomal chromosomes (Supplemental Table 1), some chromosomes show a notable enrichment or depletion of significant sites; for example, 8.87% of CpG probes on Chromosome 13 were identified as Bonferroni-significant dDMPs (relative enrichment = 1.26, *P* = 3.68 × 10^−10^), whereas only 4.09% of CpG probes on Chromosome 19 reach this criterion (relative enrichment = 0.55, *P* = 7.01 × 10^−79^). The four top-ranked hyper- and hypomethylated fetal brain dDMPs are shown in Figure 1, C and D, respectively. Overall, there is a highly significant enrichment of hypomethylated autosomal dDMPs compared with hypermethylated dDMPs (Table 2) (hypomethylated sites: *n* = 16,190 [56.4%]; hypermethylated sites: *n* = 12,528 [43.6%]; *P* = 6.74 × 10^−53^), which is consistent with previous reports (Numata et al. 2012). We find a significant correlation (r = 0.57, *P* = 2.82 × 10^−9^) between developmental DNA methylation changes at the 100 top-ranked fetal cortex dDMPs identified in a previous, smaller-scale study (Numata et al. 2012) and changes observed at the same loci in our study (Supplemental Fig. 2). Although we have not directly generated transcriptomic data on the samples profiled in this study, fetal brain gene expression data for loci annotated to the top 20 hypermethylated and top 20 hypomethylated dDMPs (listed in Table 1) were extracted from the Brain Cloud resource (http://braincloud.jhmi.edu) (Colantuoni et al. 2011). This resource contains cortex transcriptomic data from 38 human fetal samples spanning 14 to 20 gestational weeks (∼84–126 DPC). Despite the much narrower range of ages in this cohort, nine (29.03%) of the 31 transcripts for which data were available displayed a nominally significant (*P* < 0.05) association between transcript abundance and brain development (Supplemental Table 2), with several transcripts showing highly significant associations with developmental age (Supplemental Fig. 3). Many dDMPs do not represent isolated changes at specific sites but rather occur in clusters (Fig. 1E; Supplemental Fig. 4) representing developmentally differentially methylated regions (dDMRs). We used *comb-p* (Pedersen et al. 2012) to identify spatially correlated regions of differential DNA methylation significantly associated with fetal brain development (Sidak-corrected *P* < 0.05, number of probes ≥ 3). In total, 4825 dDMRs were identified with a mean size of 391 base pairs (bp) (SD = 250 bp) and spanning an average of 4.77 probes (Supplemental Table 3). The top-ranked dDMRs (ranked by Sidak-corrected *P*-value) are shown in Supplemental Table 4 and Supplemental Figure 4, and a complete list of all significant dDMRs is available for download from our laboratory website (http://epigenetics.iop.kcl.ac.uk/fetalbrain/4825_age_DMRs.csv) and in Supplemental File 2. Of note, several dDMRs are located in the vicinity of loci known to play a key role in neurodevelopment, including the Wnt antagonist *SFRP1* that regulates cell differentiation and proliferation in the developing brain (Augustine et al. 2001), the nuclear receptor gene *NR4A2*, which is important for the differentiation and maintenance of dopaminergic neurons during neurogenesis (Jankovic et al. 2005; Vuillermot et al. 2012), and *SHANK2*, encoding a synaptic protein that functions as a molecular scaffold in the developing brain which has been implicated in neurodevelopmental disorders, including autism (Schmeisser et al. 2012; Guilmatre et al. 2014).

**Table 1.**
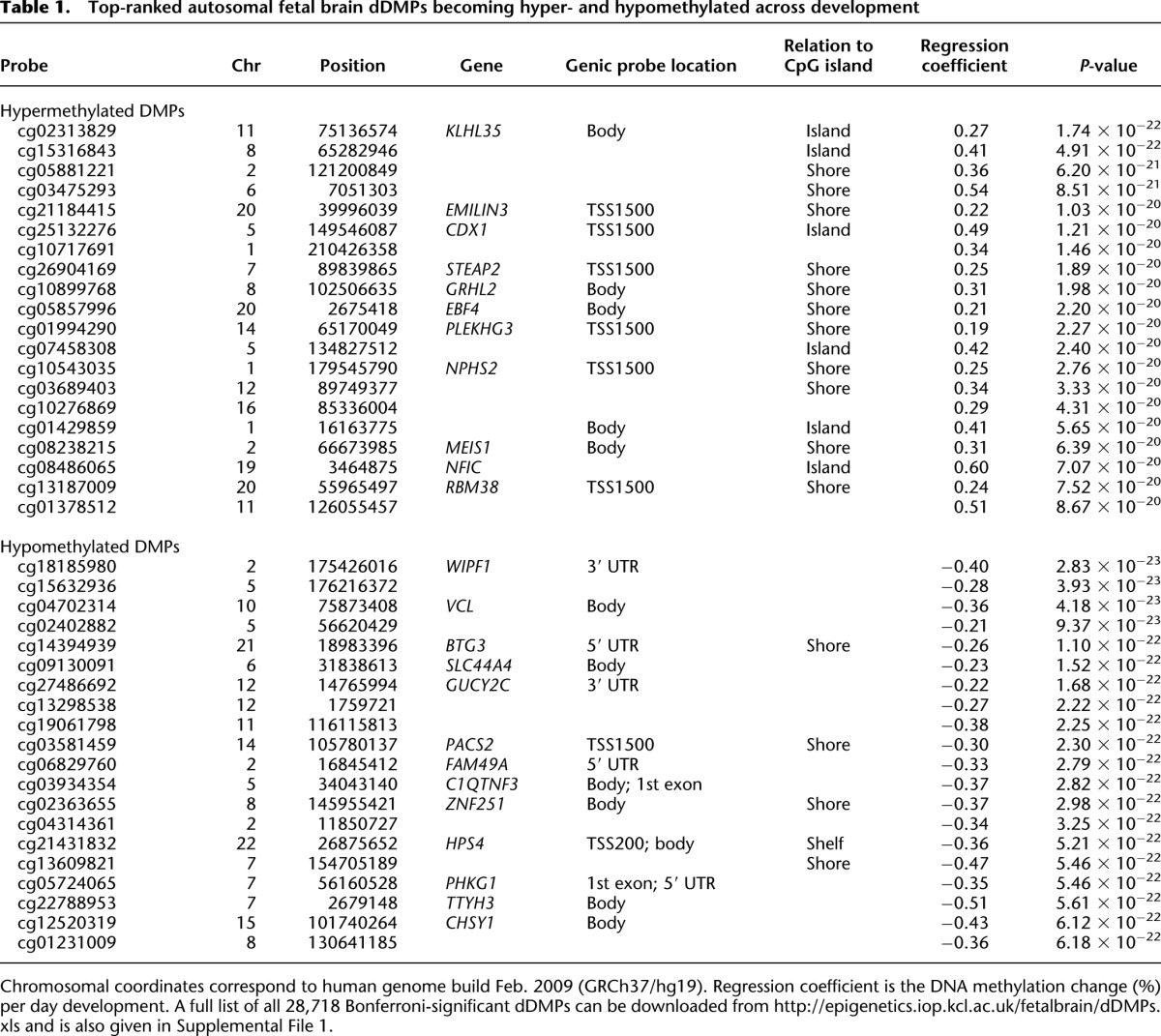
Top-ranked autosomal fetal brain dDMPs becoming hyper- and hypomethylated across development

**Table 2.**
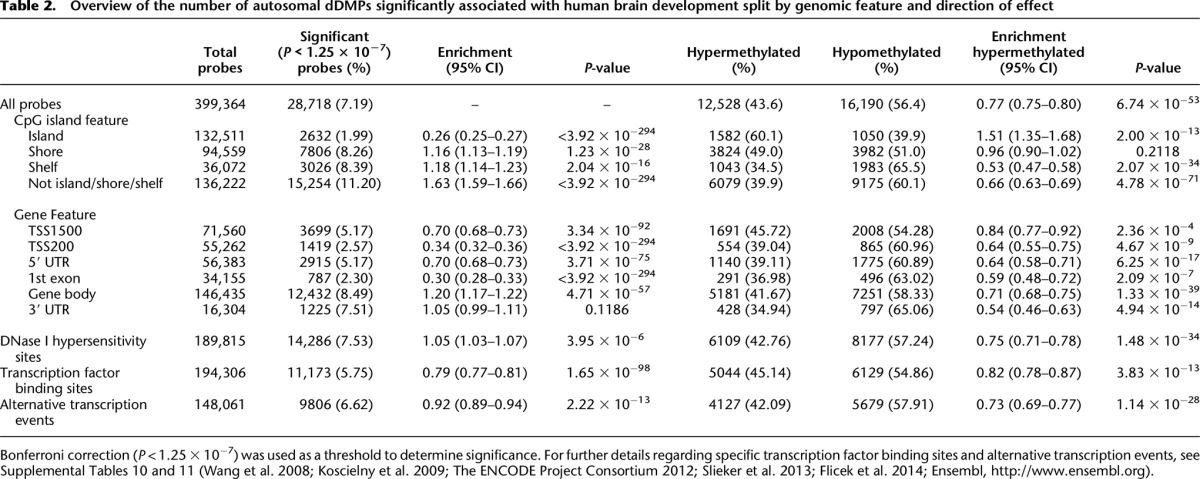
Overview of the number of autosomal dDMPs significantly associated with human brain development split by genomic feature and direction of effect

### There is a significant reduction in non-CpG DNA methylation during human fetal brain development

Of the 408,608 probes passing QC, a small subset (*n* = 743) interrogated DNA methylation at non-CpG sites. The level of DNA methylation at these sites is generally low (mean DNA methylation across all non-CpG sites = 14.93%, SD = 6.83), with the majority of probes being relatively unmethylated throughout brain development, confirming the results of a previous study reporting negligible levels of non-CpG methylation in fetal cortex (Lister et al. 2013). There is, however, a small but significant overall decrease in average non-CpG DNA methylation across the period of fetal brain development assessed in this study (r = −0.24, *P*-value = 1.14 × 10^−3^) (Supplemental Fig. 5), with 698 (93.9%) of the interrogated non-CpG probes becoming less methylated with developmental age, supporting previous data showing that non-CpG methylation is reduced following differentiation of embryonic stem cells (Lister et al. 2009). In total, 14 non-CpG sites (1.88%) demonstrated Bonferroni-significant (*P* < 6.73 × 10^−5^) changes in DNA methylation across brain development (Supplemental Fig. 6; Supplemental Table 5).

### The distribution of fetal brain dDMPs differs depending on CG density

**Figure 2.**
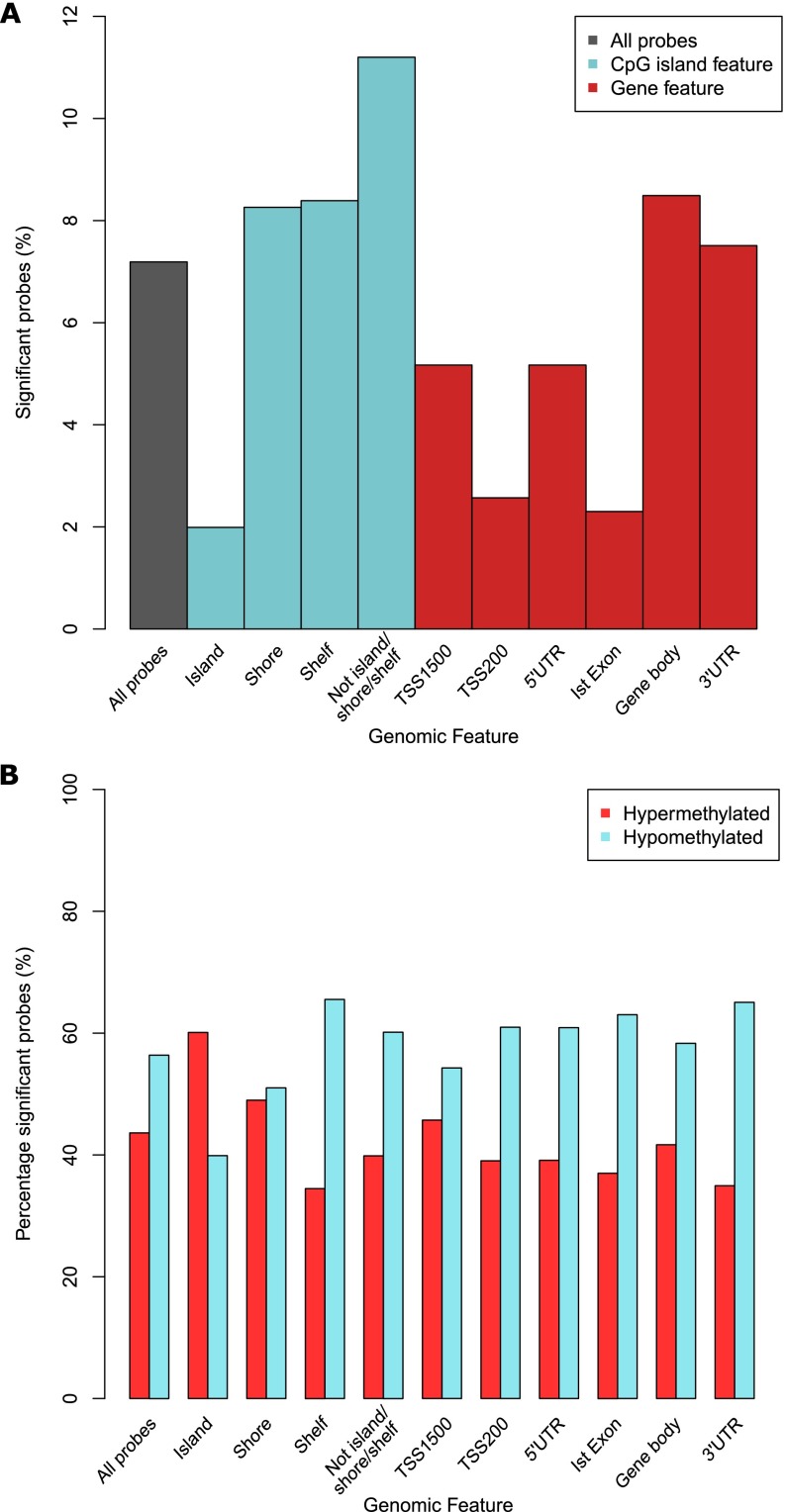
The distribution and direction of fetal brain dDMPs differs markedly across genomic features. (*A*) Compared to the genome average, dDMPs are significantly underrepresented in CpG islands, transcription start-sites, 5′ UTRs, and first exons, but significantly enriched in CpG island shores, CpG island shelves, and the gene body (see also Table 2). (*B*) There is an overall enrichment of hypomethylated fetal brain dDMPs (*P* = 6.74 × 10^−53^), but CpG islands are unique in showing an enrichment for sites becoming hypermethylated with fetal brain development (*P* = 2.00 × 10^−13^) (see also Table 2).

Although fetal brain dDMPs are seen throughout the genome, they are not equally distributed with respect to annotated genic features (Table 2). There is a significant underrepresentation of dDMPs in promoter regulatory regions, including CpG islands (CGIs) (percentage of significant probes = 1.99%, relative enrichment = 0.26, *P* < 3.92 × 10^−294^), genic first exons (percentage of significant probes = 2.30%, relative enrichment = 0.30, *P* < 3.92 × 10^−294^), 5′ untranslated regions (5′ UTRs) (percentage of significant probes = 5.17%, relative enrichment = 0.70, *P* = 3.71 × 10^−75^), and in the vicinity of annotated transcription start sites (TSSs) (TSS-200: percentage of significant probes = 2.57%, relative enrichment = 0.34, *P* < 3.92 × 10^−294^; TSS-1500: percentage of significant probes = 5.17%, relative enrichment = 0.70, *P* = 3.34 × 10^−92^) (Fig. 2A). Conversely, fetal brain dDMPs are significantly enriched in CGI shores (percentage of significant probes = 8.26%, relative enrichment = 1.16, *P* = 1.23 × 10^−28^), CGI shelves (percentage of significant probes = 8.39%, relative enrichment = 1.18, *P* = 2.04 × 10^−16^), and gene bodies (percentage of significant probes = 8.49%, relative enrichment = 1.20, *P* = 4.71 × 10^−57^). Interestingly, dDMPs within CGIs are more likely to become hypermethylated with fetal age (hypermethylated *n* = 1582 [60.1%] vs. hypomethylated *n* = 1050 [39.9%], relative enrichment = 1.51, *P* = 2.00 × 10^−13^) in contrast to dDMPs annotated to other genic features, which tend to become hypomethylated (see Table 2; Fig. 2B). Stratifying genomic regions by CpG density confirms a highly significant enrichment of dDMPs in all non-CGI genic features, with the highest percentage of significant probes found in non-CGI intergenic sites (percentage of significant probes = 13.16%, relative enrichment = 1.96, *P* = 3.92 × 10^−294^). In contrast, dDMPs were underrepresented in CG-rich regions, especially in CGI proximal promoter sites (percentage of significant probes = 0.60%, relative enrichment = 0.08, *P* < 3.92 × 10^−294^) (Supplemental Table 6; Supplemental Fig. 7).

### Fetal brain dDMPs are enriched in certain functional elements and depleted in others

A modest enrichment of dDMPs compared to the genome average was seen in DNase I hypersensitivity sites (percentage of significant probes = 7.53%, relative enrichment = 1.05, *P* = 3.95 × 10^−6^) (Table 2; The ENCODE Project Consortium 2012; Slieker et al. 2013) marking regulatory DNA regions (Maurano et al. 2012). In contrast, probes associated with transcription factor binding sites (TFBSs) (The ENCODE Project Consortium 2012; Slieker et al. 2013) were under-enriched for dDMPs compared to the genome average (percentage of significant probes = 5.75%, relative enrichment = 0.79, *P* = 1.65 × 10^−98^). In total, 117 of the 148 (79.05%) classes of TFBS motifs assessed were significantly (*P* < 0.05) under-enriched for dDMPs, with 12 (8.11%) being significantly enriched for dDMPs (see Supplemental Table 7 for a list of under- and over-enriched TFBSs). Taken together, regions characterized by alternative transcription events (Wang et al. 2008; Koscielny et al. 2009; Flicek et al. 2014; Ensembl, http://www.ensembl.org) were under-enriched for dDMPs (percentage of significant probes = 6.62%, relative enrichment = 0.92, *P* = 2.22 × 10^−13^) (Table 2), although there was considerable heterogeneity between different domains (Supplemental Table 8; Supplemental Fig. 8). There is a significant over-enrichment of dDMPs in alternative last exons (percentage of significant probes = 9.82%, relative enrichment = 1.41, *P* = 1.05 × 10^−21^), cassette exons (percentage of significant probes = 8.24%, relative enrichment = 1.16, *P* = 8.09 × 10^−21^), and mutually exclusive exons (percentage of significant probes = 8.11%, relative enrichment = 1.14, *P* = 4.62 × 10^−5^), with all other events displaying a significant under-enrichment of dDMPs.

### There are sex differences and distinct sex-specific developmental trajectories in the human fetal brain methylome, including at autosomal locations

**Figure 3.**
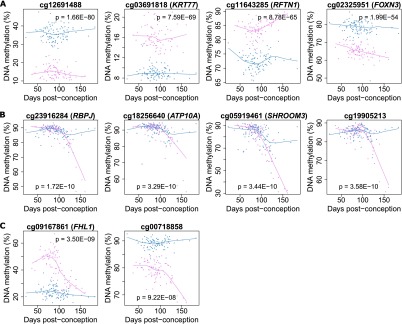
Sex-specific DNA methylation differences in the developing fetal brain. A total of 8059 (1.99% of the 408,594 autosomal and X-linked probes assessed) were identified as significantly differentially methylated between males and females in the fetal brain, with most differences occurring on the X Chromosome as expected. However, a number of autosomal loci also show significant differences between males and females, as illustrated by the four probes shown in *A*. Of note, DNA methylation at a number of (*B*) autosomal and (*C*) X Chromosome probes showed significant interactions between sex and developmental stage, indicating differential trajectories of DNA methylation in the developing male and female brain.

A total of 8059 (1.99% of the 408,594 autosomal and X-linked probes assessed) were identified as significantly (Bonferroni-corrected threshold: *P* < 1.22 × 10^−7^) differentially methylated between all male and female samples (Supplemental Fig. 9). As expected, the majority (*n* = 7538, 93.53%) of these differences are located on the X Chromosome and are likely associated with X Chromosome dosage compensation mechanisms in females. However, an appreciable proportion of sex differences (*n* = 521, 6.47%) are autosomal (see Fig. 3A and Table 3 for examples). Of these, approximately half are hypermethylated in females (*n* = 261, 50.10%). A full list of all 521 Bonferroni-significant autosomal sex DMPs can be downloaded from our website (http://epigenetics.iop.kcl.ac.uk/fetalbrain/sDMPs.xlsx) and is also given in Supplemental File 3. Of the 521 sites showing sex differences, 119 overlap with significant autosomal DNA methylation differences identified between males and females in a recent analysis of the postnatal adult cortex (Xu et al. 2014), with 99.16% (*n* = 118) of these displaying the same direction of effect (Supplemental Table 9). Furthermore, there is a highly significant correlation (r = 0.88, *P* < 1 × 10^−200^) between the magnitude and direction of sex differences in DNA methylation at autosomal probes reported as significantly different in the Xu et al. (2014) analysis of adult brain (*n* = 544) and sex differences at the same CpG sites in fetal brain (Supplemental Fig. 10), indicating that most gender differences in the brain methylome manifest early in fetal development and are stable across the life course. A total of 1099 DMRs between males and females in the fetal brain were identified (Sidak-corrected *P* < 0.05, number of probes ≥ 3) (Supplemental Table 3), of which the majority (*n* = 908, 82.62%) are located on the X Chromosome, with 191 (17.38%) located on the autosomes (see Supplemental Table 10; Supplemental Fig. 11). A list of all significant sex-associated DMRs is available from our laboratory website (http://epigenetics.iop.kcl.ac.uk/fetalbrain/1099_sex_DMRs.csv) and in Supplemental File 4. We next explored sex-specific changes in DNA methylation across fetal brain development by analyzing interactions between sex and developmental age. We identified 61 sites (*n* = 59 autosomal, *n* = 2 X Chromosomes) characterized by significant (Bonferroni-corrected threshold: *P* < 1.22 × 10^−7^) sex-specific DNA methylation trajectories across brain development (Table 4; Supplemental Fig. 12). The top-ranked autosomal sex-specific dDMP (cg23916284, *P* = 1.72 × 10^−10^) (Fig. 3B) is located in the gene body of *RBPJ*, which encodes a transcriptional regulator in the Notch signaling pathway that is involved in neurogenesis and neuronal maturation. The pattern of DNA methylation observed at this site is typical of other autosomal sex-specific dDMPs; a high level of DNA methylation is observed in both males and females until ∼100 DPC, when females diverge from males, becoming progressively hypomethylated. These findings should be treated with some caution given the low number of samples at these later gestational ages. The two significant X Chromosome sex-specific dDMPs (cg09167861 [*FHL1*], *P* = 3.50 × 10^−9^; cg00718858, *P* = 9.22 × 10^−8^) show more distinct male and female trajectories across brain development (Fig. 3C) and potentially reflect dynamic changes in X Chromosome dosage compensation mechanisms. Using *comb-p*, we identified 66 DMRs displaying sex-specific DNA methylation trajectories across brain development (Supplemental Fig. 13; Supplemental Tables 3, 11). A complete list of regions characterized by sex-specific DNA methylation trajectories can be downloaded from http://epigenetics.iop.kcl.ac.uk/66_age_sex_DMRs.csv and is also given in Supplemental File 5.

**Table 3.**
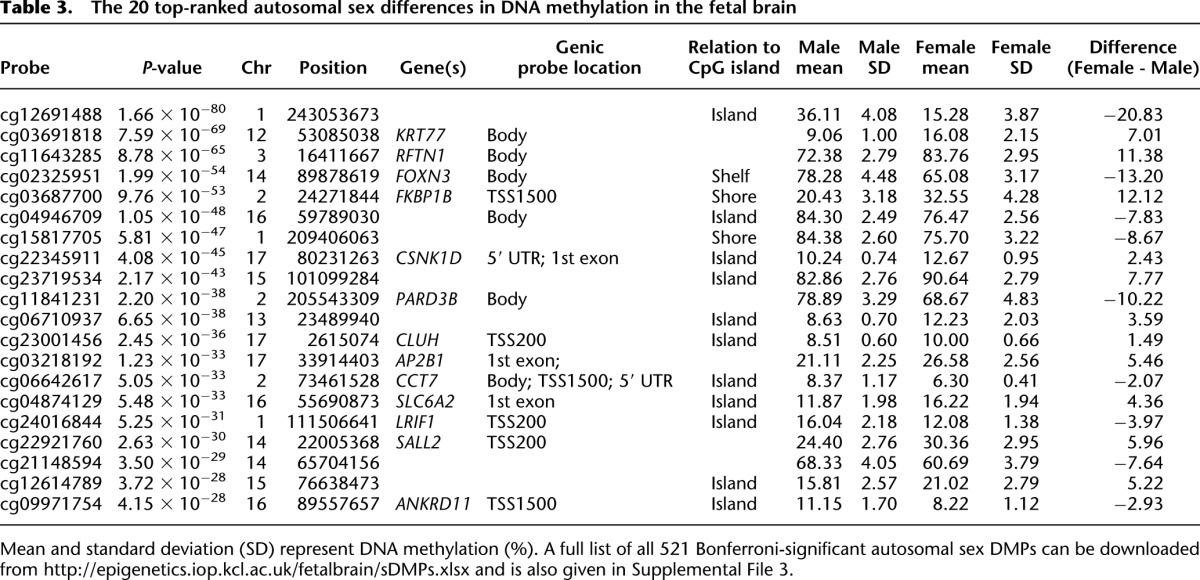
The 20 top-ranked autosomal sex differences in DNA methylation in the fetal brain

**Table 4.**
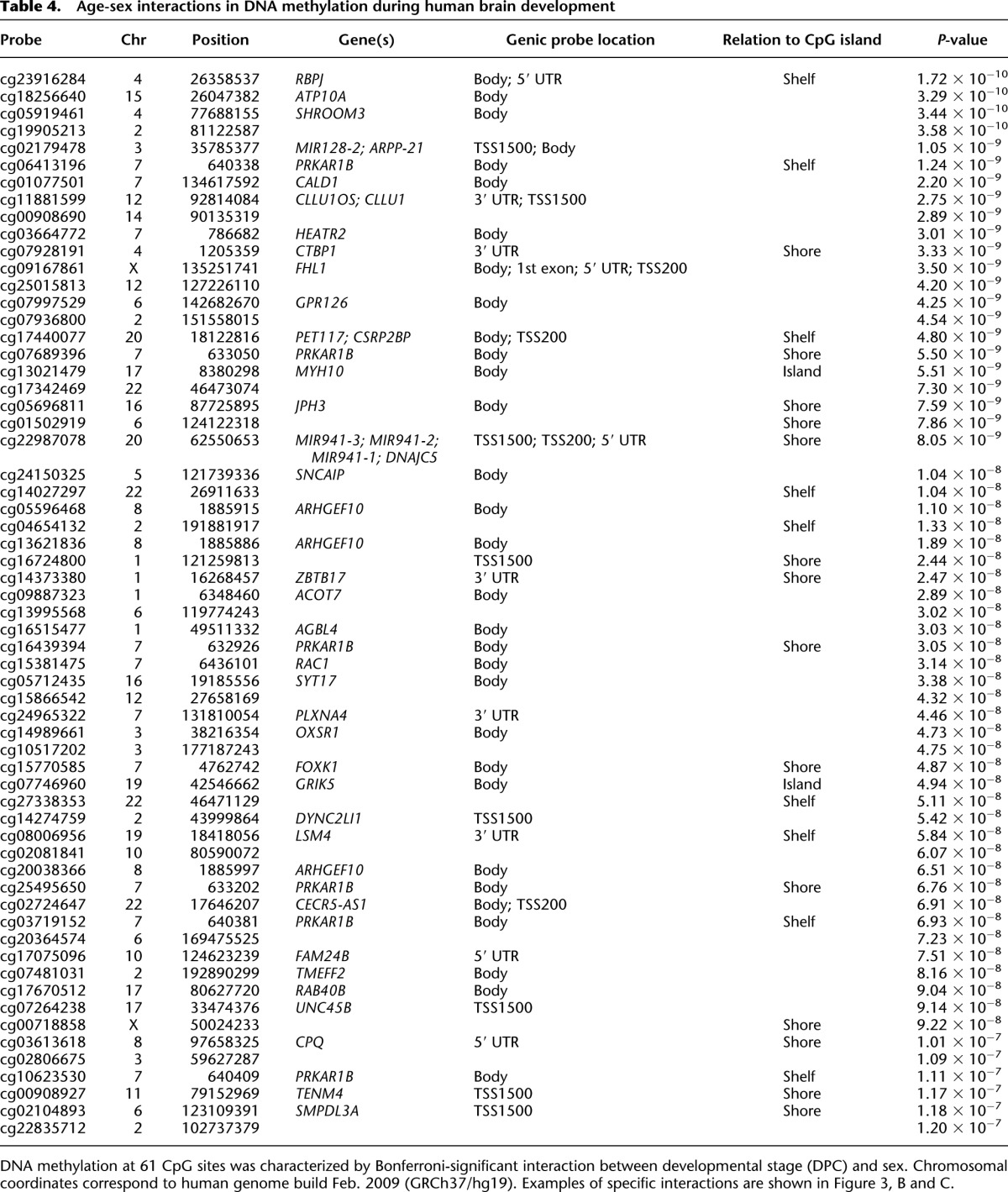
Age-sex interactions in DNA methylation during human brain development

### There are distinct modules of comethylated loci in the developing human brain

**Figure 4.**
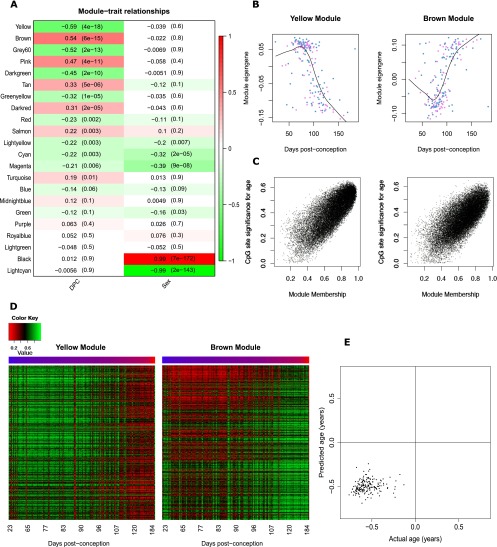
Gene comethylation modules in the developing human brain. (*A*) Heatmap representing the correlation between module eigenvalues (ME) and the samples traits of DPC and sex. Each row represents a module, as indicated on the *y*-axis, and each column a trait. As shown in the color scale bar, strong positive correlation is indicated by dark red, strong negative correlation is indicated by dark green, and white indicates no correlation. Each cell contains the corresponding correlation and *P*-value given in parentheses. The yellow (*P* = 3.94 × 10^−18^) and brown (*P* = 5.63 × 10^−15^) WCGNA modules are most significantly associated with human fetal brain development. (*B*) The module eigengene of the yellow and brown modules is significantly associated with brain development, and (*C*) module membership in both modules is strongly correlated with probe significance with brain development (yellow module: r = 0.79, *P* < 1 × 10^−200^; brown module: r = 0.72, *P* < 1 × 10^−200^). Probe significance is the absolute value of the correlation between DNA methylation at a CpG site and developmental age, and module membership is defined as the correlation between the module eigengene and the probe methylation profile. (*D*) Heatmap of probes in the yellow and brown modules showing changes across brain development. Color corresponds to DNA methylation level at probes with a module membership >0.85. (*E*) There is a significant correlation (r = 0.15, *P* = 0.038) between predicted and actual age at the resolution of days.

We next employed weighted gene comethylation network analysis (WGCNA) (Langfelder and Horvath 2008) to undertake a systems-level characterization of the DNA methylation changes associated with human brain development. WGCNA identified 22 discrete modules of comethylated positions (Supplemental Table 12), and the first principal component of each individual module (the “module eigengene”) was used to assess their relationship with fetal brain development (Fig. 4A). Nine modules were found to be significantly correlated with DPC, most notably the “yellow” (*n* = 21,999 probes, r = −0.59, *P* = 3.94 × 10^−18^) and “brown” modules (*n* = 24,002 probes, r = 0.54, *P* = 5.63 × 10^−15^) (Fig. 4B; Supplemental Fig. 14). Module membership within these modules is highly correlated with probe significance (yellow module: r = 0.79, *P* < 1 × 10^−200^; brown module: r = 0.72, *P* < 1 × 10^−200^) (Fig. 4C), showing a clear relationship between DNA methylation at the core members of each module and fetal brain development (Fig. 4D). To test whether the identified modules were biologically meaningful, over-enrichment analyses (see Methods) were performed using gene ontology (GO) pathways (Ashburner et al. 2000). Both modules were highly enriched for specific functional pathways directly related to development of the nervous system and neurobiological function (Supplemental Tables 13, 14). In particular, the brown module was highly enriched for pathways related to development of the brain including epithalamus development (odds ratio = 78.18, *P* = 3.79 × 10^−28^), central nervous system neuron axonogenesis (odds ratio = 8.26, *P* = 9.70 × 10^−21^), neuron maturation (odds ratio = 6.97, *P* = 9.78 × 10^−15^), and nervous system development (odds ratio = 1.61, *P* = 1.25 × 10^−13^). In contrast, modules not associated with fetal brain development showed no significant enrichment of neurodevelopmental functions (for example, see Supplemental Table 15 for pathways associated with the light green WGCNA module). The two modules strongly associated with sex across all fetal brain samples (light cyan, Supplemental Table 16; black, Supplemental Table 17) were, as expected, primarily comprised of X-linked loci and enriched for pathways relating to epigenetic processes (e.g., negative regulation of histone H3K36 methylation, odds ratio = 63.83, *P* = 7.34 × 10^−18^, light cyan module), sex-specific genomic regulation (e.g., sex determination, odds ratio = 6.97, *P* = 7.45 × 10^−19^, black module), and brain development (e.g., establishment or maintenance of neuroblast polarity, odds ratio = 43.97, *P* = 7.26 × 10^−33^, black module). These analyses suggest that the large coordinated changes in DNA methylation identified across our samples relate specifically to development of the brain. It is, however, noteworthy that a recent “epigenetic clock” algorithm developed by Steve Horvath (Horvath 2013), based on DNA methylation values at 353 CpG sites shown to be associated with postpartum age, accurately predicts that each of our samples is prenatal, with a significant positive correlation (r = 0.15, *P* = 0.038) between predicted and actual age at the resolution of days (Fig. 4E). This supports the notion that a core set of loci are likely to be more generally associated with aging across the life course and that these basic aging processes start very early in fetal life and occur across tissues.

### dDMPs are common in several genomic regions associated with two neurodevelopmental disorders (schizophrenia and autism)

**Figure 5.**
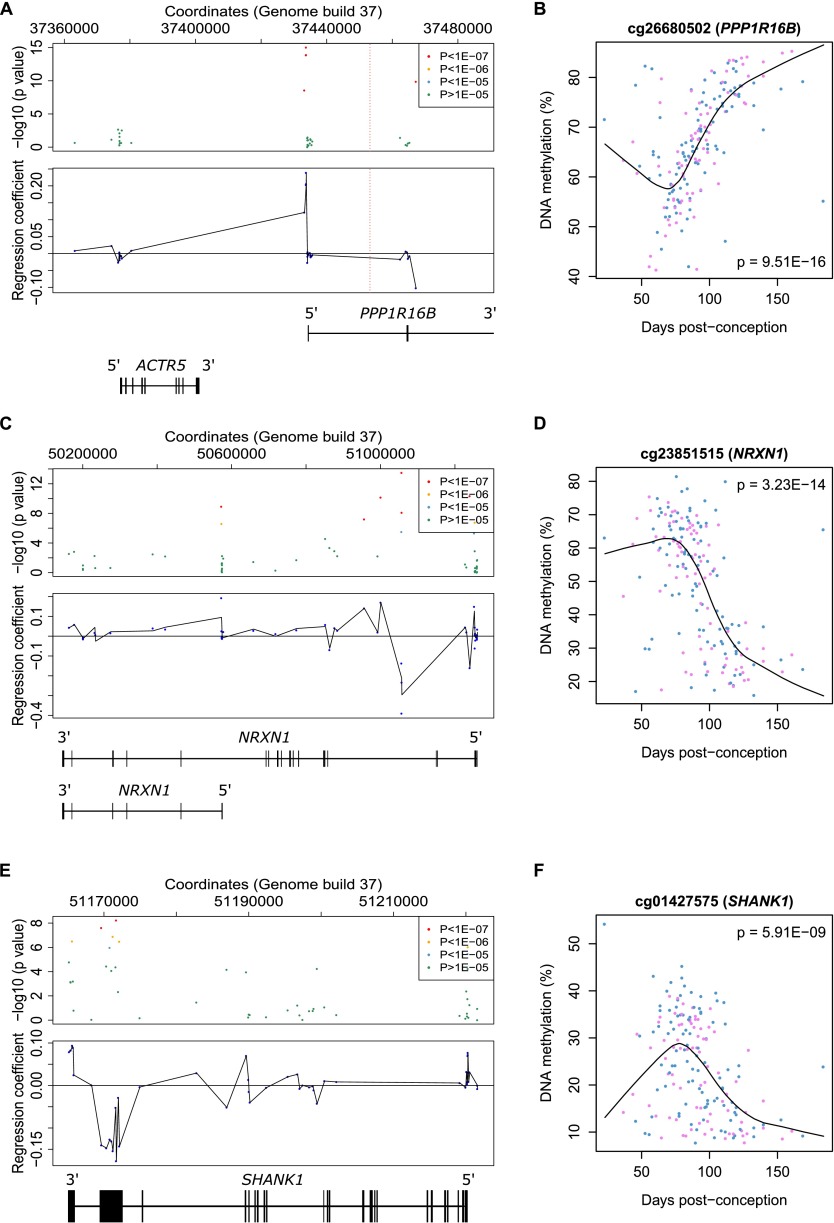
Fetal brain dDMPs in the vicinity of genetic loci associated with neurodevelopmental disorders. (*A*) Fetal brain dDMPs within high-confidence regions identified in a recent large GWAS of schizophrenia may provide insight regarding causal variants, e.g., region Chr 20: 37361494–37485994 contains two genes, of which one overlaps with five highly significant dDMPs: *PPP1R16B*. The position of the most significant SNP (rs6065094) is indicated by a dashed red line. (*B*) The top-ranked dDMP (cg26680502) in the schizophrenia-associated region displays significant hypermethylation across fetal brain development (*P* = 9.51 × 10^−16^). (*C*) Examples of dDMPs in the vicinity of autism candidate gene *NRXN1*, with (*D*) the top-ranked *NRXN1* probe (cg23851515) becoming significantly hypomethylated across fetal brain development (*P* = 3.23 × 10^−14^). (*E*) Examples of dDMPs in the vicinity of autism candidate gene *SHANK1*, with (*F*) the top-ranked probe (cg01427575) becoming significantly hypomethylated across fetal brain development (*P* = 5.91 × 10^−9^).

Schizophrenia and autism spectrum disorder are highly heritable neuropsychiatric disorders that are believed to have an early neurodevelopmental component (Weinberger 1995; Geschwind and Levitt 2007; Fatemi and Folsom 2009). A recent large-scale genome-wide association study (GWAS) of schizophrenia (Schizophrenia Working Group of the Psychiatric Genomics Consortium 2014) identified 108 conservatively defined loci meeting genome-wide significance. To aid the functional characterization of these loci and identify genes potentially involved in the neurodevelopmental origins of this disorder, we provide annotation of the fetal brain dDMPs within the 89 genomic regions containing at least one informative CpG site (Supplemental Table 18). Of note, several specific schizophrenia-associated genomic regions contain multiple dDMPs (for example, see Fig. 5A,B), highlighting regions of potential interest for understanding the neurodevelopmental origins of this disorder. Likewise, for autism, high-confidence candidate gene regions were selected from the Simons Foundation Autism Research Initiative (SFARI) gene database (Basu et al. 2009; Abrahams et al. 2013) and complemented by loci identified in recent exome sequencing and genomic studies (Neale et al. 2012; O’Roak et al. 2012a,b; Sanders et al. 2012; Willsey et al. 2013). The number of dDMPs in each of the genomic regions assessed can be seen in Supplemental Table 19, with clusters of fetal brain dDMPs observed in the vicinity of autism-associated genes with known neurodevelopmental functions, including *NRXN1* (Fig. 5C,D) and *SHANK1* (Fig. 5E,F). As autism is known to have a substantial sex bias (Werling and Geschwind 2013), we also investigated whether any of the regions containing high-confidence autism risk genes are subject to sex differences in fetal brain methylation, which could mediate this effect. Of the 19 genomic regions investigated, only one autosomal probe, annotated to *SHANK1*, displayed sex differences in DNA methylation at a Bonferroni-corrected threshold of *P* < 7.05 × 10^−5^.

## Discussion

In this study, we assessed neurodevelopmental methylomic trajectories in 179 human fetal brain samples (100 male, 79 female) spanning 23 to 184 DPC. To our knowledge, this represents the most extensive methylomic study of human fetal brain to date. Our data indicate that considerable epigenomic changes take place in the human brain during fetal brain development. We observe Bonferroni-significant changes in DNA methylation at >7% of sites assessed, with sex-specific methylomic trajectories observed for multiple dDMPs including dDMPs on autosomes. Our data also reveal modules of comethylated loci associated with fetal age, which are significantly enriched for genes involved in neurodevelopmental processes.

Although there is an overall enrichment of sites becoming less methylated during fetal brain development, several genomic regions are characterized by distinctly different developmental patterns. DNA methylation in CpG-rich regions, for example, appears to be considerably less dynamic during fetal brain development than other genomic regions. Furthermore, although CpG-rich regions are characterized by an enrichment of hypermethylated sites compared to hypomethylated sites, the majority of dDMPs in the genome become hypomethylated with brain development. It is not surprising that CpG islands are relatively less dynamic than other regions interrogated in this study given their role in regulating the stable expression of key housekeeping loci (Deaton and Bird 2011). Additionally, we observe an enrichment of dDMPs within the gene body, and these sites tend to display decreasing levels of DNA methylation with age. Interestingly, gene body DNA methylation is often positively correlated with gene expression (Ball et al. 2009; Maunakea et al. 2010) and has been hypothesized to enhance transcription elongation efficiency, suppress the spurious initiation of transcription within active genes, contribute to splicing mechanisms, and regulate alternative promoter use. These changes in gene body DNA methylation during brain development is therefore interesting given the dramatic transcriptional plasticity occurring in the brain at this time (Colantuoni et al. 2011).

Due to the differences in prevalence observed between males and females for many neurodevelopmental disorders, it is important to understand sex-specific processes in brain development (Baron-Cohen et al. 2005). Our data demonstrate notable sex differences in the fetal brain methylome, including at autosomal loci. Many of these overlap with autosomal DNA methylation sex differences in the adult cortex (Xu et al. 2014), indicating that sex-differences in the brain methylome manifest during fetal development and are stable across life. We also identify sex differences in DNA methylation trajectories across fetal brain development, with DNA methylation at a number of loci showing significant interactions between sex and fetal age. The top-ranked CpG site showing a sex-specific neurodevelopmental trajectory is located in the *RBPJ* gene, which encodes a transcriptional regulator in the Notch signaling pathway that is directly involved in neurogenesis and neuronal maturation (Louvi and Artavanis-Tsakonas 2006; Fujimoto et al. 2009; Imayoshi et al. 2010). Of note, recent evidence suggests there is considerable interaction between Notch signaling and sex hormones including estradiol (Soares et al. 2004; Arevalo et al. 2011; Ruiz-Palmero et al. 2011). Sex hormones are believed to contribute to the sexual differentiation of the brain that occurs in the second half of pregnancy (Bakker and Brock 2010) and are known to be potent modifiers of epigenetic status and transcription in the brain (Ghahramani et al. 2014). In this regard, it is interesting that male-female divergence at many loci occurs at ∼100 DPC (see Fig. 3B), coinciding with a testosterone peak occurring between weeks 12 and 18 of pregnancy that is believed to mediate sexual differentiation of the brain (Finegan et al. 1989).

Schizophrenia and autism spectrum disorder are common neuropsychiatric disorders that are believed to have an early neurodevelopmental component (Weinberger 1995; Geschwind and Levitt 2007; Fatemi and Folsom 2009). Understanding the epigenomic changes occurring during fetal brain development could therefore provide useful insight about the molecular etiology of these conditions, especially given recent evidence for disease-associated epigenetic dysfunction in the brain (Mill et al. 2008; Ladd-Acosta et al. 2013; Pidsley et al. 2014). We include the location of dDMPs in relation to high-confidence schizophrenia- and autism-associated loci, which could provide clues to the location of functional genetic variants contributing to the etiology of these disorders.

There are several limitations to this study. First, legal restrictions on later-term abortions precluded the assaying of brain samples from later stages of fetal development. However, in comparison with previous studies, we have assessed a much larger number of brain samples spanning a wider range of fetal ages (Numata et al. 2012; Lister et al. 2013). For example, the largest previous study (Numata et al. 2012) assayed only 30 prenatal samples spanning ∼100 to 130 DPC. Another limitation is that the brain tissue used in the present study was acquired frozen and undissected, and was therefore processed as a composite of brain regions and cell types. Although this precludes us from specifying a particular brain region or cell type from which our data are derived, our results have the potential advantage of being representative of the predominant changes in the developing human brain as a whole. Third, although the Illumina 450K platform can accurately quantify DNA methylation at single-base resolution with probes associated with 99% of genes and 96% of CGIs, the array targets <2% of the CpG sites in the human genome, and probes are not equally distributed across all genomic features. As costs diminish, future studies should employ sequencing-based genomic-profiling technologies to more thoroughly interrogate the epigenome across development in large numbers of samples. Fourth, it is likely that the Bonferroni-corrected *P*-value cutoff applied in this study is overly conservative due to the nonindependence of CpG sites (Shoemaker et al. 2010). Although we report that ∼7% of probes represent true fetal brain dDMPs, the reality is likely to be higher. Fifth, no demographic or phenotypic information other than developmental age was available for the samples assayed, although we were able to determine sex using an XY Chromosome PCR assay and the DNA methylation data. Sixth, we do not have gene expression data from these samples and are therefore unable to make direct conclusions about the transcriptional consequences of the developmental changes in DNA methylation we observe. Using publicly available microarray gene expression data from an independent set of fetal brain samples, however, we find evidence that a number of genes in the vicinity of our top-ranked dDMPs are differentially expressed across brain development. Given the low number of samples and limited developmental ages available in the resource utilized (Colantuoni et al. 2011), as well as the lack of information on alternative splicing and isoform abundance, these data suggest that many of the fetal brain dDMPs will have transcriptional consequences in the developing brain. Finally, we are not able to distinguish between DNA methylation and its oxidized derivative, DNA hydroxymethylation, as standard bisulfite sequencing does not distinguish between these modifications (Huang et al. 2010). This is an important confounder given that the latter is known to be abundant and to have functional relevance in the brain (Globisch et al. 2010; Khare et al. 2012; Lister et al. 2013). It will therefore be important to further characterize these modifications across fetal brain development using techniques capable of discriminating DNA methylation and DNA hydroxymethylation (Booth et al. 2012), as well as additional epigenetic modifications, including the more highly oxidized DNA methylation derivatives, 5-formylcytosine and 5-carboxycytosine, and histone modifications (Plongthongkum et al. 2014).

In conclusion, we identify highly dynamic hypo- and hypermethylation occurring throughout the genome during human fetal brain development, with notable sex-specific methylomic trajectories observed for several dDMPs. There are discrete modules of comethylated loci associated with brain development that are significantly enriched for genes involved in neurodevelopmental processes. Our data indicate that epigenetic changes during human brain development are highly dynamic, sometimes sex-specific, and may provide clues to the origins of common neurodevelopmental disorders.

## Methods

### Samples

Human fetal brain tissue was acquired from the Human Developmental Biology Resource (HDBR) (http://www.hdbr.org) and the MRC Brain Banks network (http://www.mrc.ac.uk/research/facilities/brain-banks/access-for-research). Ethical approval for the HDBR was granted by the Royal Free Hospital research ethics committee under reference 08/H0712/34 and Human Tissue Authority (HTA) material storage license 12220; ethical approval for the MRC Brain Bank was granted under reference 08/MRE09/38. The age of these samples ranged from 23 to 184 DPC, determined by Carnegie staging in the case of embryonic samples (defined as ≤56 DPC) and foot and knee to heel length measurements for fetal samples (defined as ≥57 DPC). No additional phenotypic or demographic information was available. An overview of the samples is given in Figure 1A and Supplemental Table 20. Brain tissue was obtained frozen and had not been dissected into regions. We therefore homogenized half of the brain tissue from each individual fetus for subsequent genomic DNA extraction, which was performed by standard phenol-chloroform procedures. Sample sex was determined via PCR amplification of a region in the vicinity of the amelogenin gene (*AMELY*) that produces different sized PCR products for the X and Y Chromosomes (977 and 788 bp, respectively) (Eng et al. 1994). Following methylation array processing, sex-calls were confirmed using multidimensional scaling plots of the variable XY Chromosome probes (see below).

### Genome-wide DNA methylation quantification

All samples were randomized with respect to age and sex to avoid batch effects throughout all experimental procedures. Genomic DNA (500 ng) from each sample was treated in duplicate with sodium bisulfite using the Zymo EZ DNA Methylation-Lightning Kit (Zymo Research). Genome-wide DNA methylation was quantified using the Illumina Infinium HumanMethylation450 BeadChip (Illumina) and scanned on the HiScan System (Illumina). Illumina GenomeStudio software (Illumina) was used to extract signal intensities for each probe, generating a final report that was imported into the R statistical environment 3.0.2 (http://www.r-project.org) (R Development Core Team 2005) using the *methylumi* and *minfi* packages (http://www.bioconductor.org/packages/release/bioc/html/methylumi.html). Data quality control and preprocessing were performed using the *wateRmelon* package as described previously (Pidsley et al. 2013). Stringent filtering of the prenormalized Illumina 450K data was performed using the *pfilter* function; CpG sites with a beadcount of <3 in 5% of samples were removed (*n* = 146) as were CpG sites with a detection *P*-value > 0.05 in 1% of samples (*n* = 22,379). Finally, cross-reactive probes and polymorphic CpGs, as detailed in the Illumina annotation file and identified in recent publications (Chen et al. 2013; Price et al. 2013), were removed (Supplemental Table 21), leaving 408,608 probes for further analysis (including *n* = 9230 X Chromosome and *n* = 14 Y Chromosome probes). Data were normalized with the *dasen* function of the *wateRmelon* package. Demographic information for the final cohort of the 179 samples remaining following quality control (*n* = 100 males [age range = 23 to 184 DPC]; *n* = 79 females [age range = 37 to 161 DPC]) is shown in Supplemental Table 20.

### Statistical analysis

A multiple linear regression model with DPC and sex was used to identify methylation changes associated with brain development or sex. Autosomal probes (*n* = 399,364) were considered significantly associated with DPC if they passed a Bonferroni-corrected significance threshold of *P* < 1.25 × 10^−7^ (α = 0.05), while autosomal and X Chromosome probes (*n* = 408,594) were considered to be significantly associated with sex if they passed a Bonferroni-corrected significance threshold of *P* < 1.22 × 10^−7^ (α = 0.05). The model was then extended to include an interaction term between sex and DPC and fitted for all autosomal and X-linked probes (*n* = 408,594); a significance threshold of *P* < 1.22 × 10^−7^ (α = 0.05) was used to identify probes with different developmental methylation trajectories between the sexes.

### WGCNA analysis

Weighted gene comethylation network analysis (WGCNA) was used to identify modules of highly comethylated probes (Langfelder and Horvath 2008). Modules were identified from their pairwise correlations, using a signed block-wise network construction at power 4. Modules identified were assigned a color name, and a weighted average-methylation profile, known as a module eigengene (ME), was calculated for each. Correlations between the MEs and the phenotypic traits available (DPC, sex) were used to identify modules associated with these traits. The relationship between each probe and each module was assessed through calculation of module membership (MM), the absolute correlation between a probe’s DNA methylation status and the ME, allowing the identification of the subset of probes with the strongest membership to each module. Probe significance (PS), the absolute value of correlation between each probe and phenotypic trait, was calculated to quantify the association of probes with age. To assess the biological meaning of modules associated with age and sex, genes associated with probes possessing an absolute MM value of >0.85 were extracted and assessed using pathway and gene ontology analysis.

### Gene ontology and pathway analysis

Gene annotations provided by Illumina were used to map probes to genes. Gene ontology terms were downloaded and annotated to all probes based on these gene annotations where all parent terms for each child term were also included. Probes without genic annotation or without any gene ontology annotation were excluded from the subsequent analysis. Fisher’s exact tests were used to test for an over-enrichment of WGCNA module probes in each pathway compared to all probes passing QC and inputted to the WGCNA algorithm. Only pathways containing at least 50 probes were considered. By running the analysis at the probe level, we were able to control for the uneven distribution of probes per gene, minimizing any bias introduced by genes with an above average number of probes.

### Enrichment analyses in genomic regions associated with autism

Candidate loci were selected from the SFARI gene database (selecting genes having an evidence level of 1 [high confidence] or 2 [strong candidate]) (Basu et al. 2009; Abrahams et al. 2013), supplemented with genes implicated by exome sequencing studies (Neale et al. 2012; O’Roak et al. 2012a,b; Sanders et al. 2012), as well as those identified as high-confidence ASD genes in a recent study (Willsey et al. 2013). Probes annotated to these genes were tested for enrichment of dDMPs using with a two-tailed Fisher’s exact test, compared to the frequency of dDMPs in all gene-annotated probes on the Illumina 450K array (38,845 [11.03%] of 352,191 probes).

## Data access

Raw and normalized Illumina 450K methylation data has been submitted to the NCBI Gene Expression Omnibus (GEO; http://www.ncbi.nlm.nih.gov/geo/) under accession number GSE58885. A searchable database of our fetal brain DNA methylation data is available at http://epigenetics.iop.kcl.ac.uk/fetalbrain.

## Supplementary Material

Supplemental Material
