# Supplementary material for: Methylomic trajectories across human fetal brain development
Source: Genome Res. 2015 Mar;25(3):338–52. doi: 10.1101/gr.180273.114 (PMC4352878; doi:10.1101/gr.180273.114)
Supplement: Supplemental Material [file supp_25_3_338__index.html]

Methylomic trajectories across human fetal brain development — Methylomic trajectories across human fetal brain development — Supplemental Material 

# Methylomic trajectories across human fetal brain development

## Supplemental Material

**Files in this Data Supplement:**

- Supplemental Figures.pdf
- Supplemental File1.csv
- Supplemental File2.csv
- Supplemental File3.csv
- Supplemental File4.csv
- Supplemental File5.csv
- Supplemental Tables.docx
