# Supplementary material for: Methylomic trajectories across human fetal brain development
Source: Genome Res. 2015 Mar;25(3):338–52. doi: 10.1101/gr.180273.114 (PMC4352878; doi:10.1101/gr.180273.114)

SUPPLEMENTARY FIGURE 1 – Global levels of DNA methylation do not differ significantly across human fetal brain development (r = -0.02, p = 0.76). As expected females show slightly higher global DNA methylation compared to males (female β=0.4869, male β=0.4865, p = 0.0012).

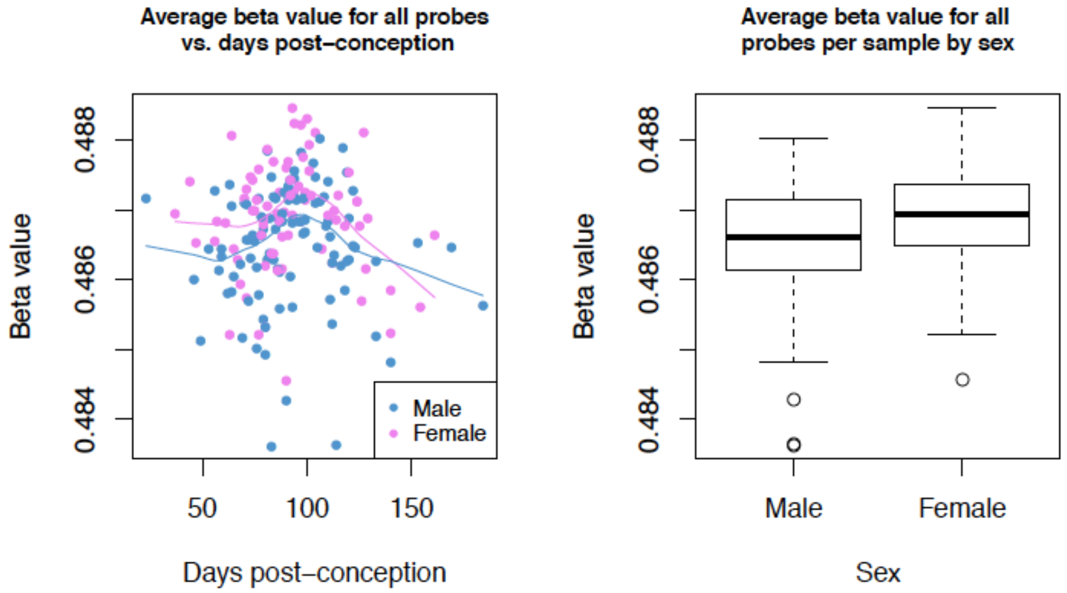

SUPPLEMENTARY FIGURE 2 – Significant correlation (r = 0.57, p = 2.82E-09) between developmental DNA methylation changes at the 100 top-ranked dDMPs reported by Numata *et al.*, (2012) and changes observed at the same loci in our study.

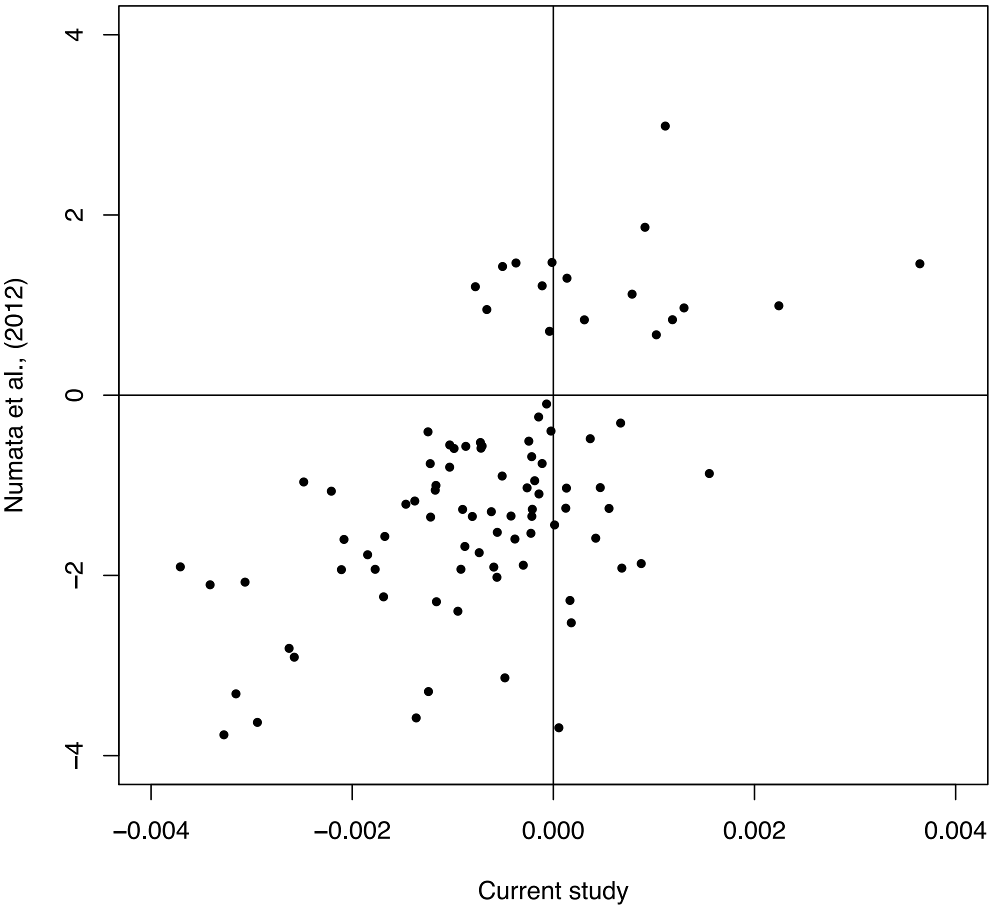

SUPPLEMENTARY FIGURE 3 – The expression of several genes associated with the top-ranked hypermethylated and hypomethylated probes is associated with brain development (see also **Supplementary Table 2**). Age refers to the age of subject at death in years, with negative numbers referring to prenatal age in years calculated back from day 0, the day of expected birth after 40 gestational weeks. Gene expression data was obtained from the Brain Cloud resource (http://braincloud.jhmi.edu) (Colantuoni et al. 2011)

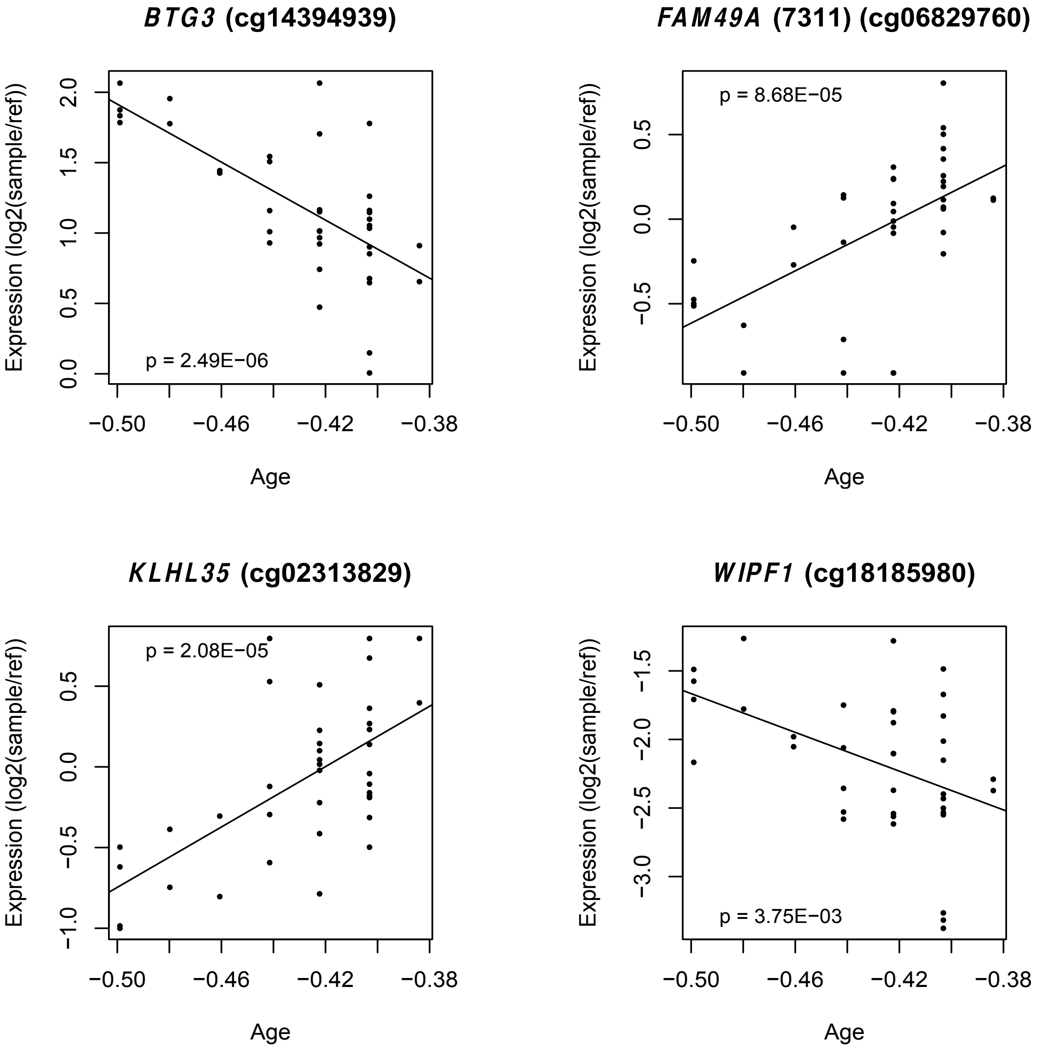

SUPPLEMENTARY FIGURE 4 – The top-ranked dDMR associated with fetal brain development spans a 822bp region upstream of the *SFRP1* gene (Chr8:41167660 – 41168482). See **Supplementary Table 4** for other top-ranked dDMRs. A full list of dDMRs is available for download from our laboratory website – http://epigenetics.iop.kcl.ac.uk/fetalbrain/4825_age_DMRs.csv. The dDMR is denoted by dashed lines. Chromosomal coordinates correspond to human genome build Feb. 2009 (GRCh37/hg19).

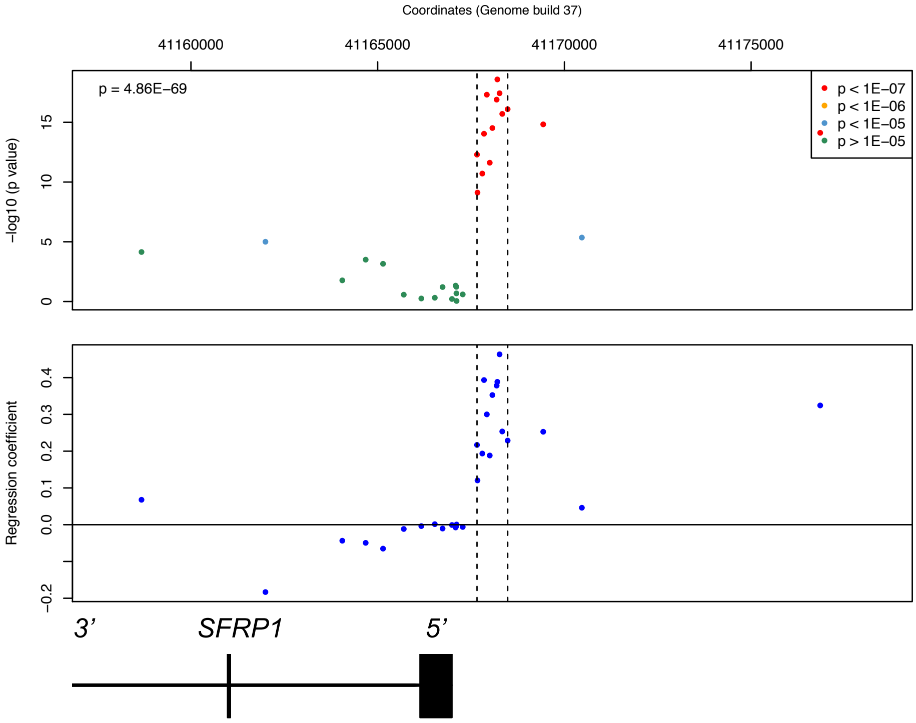

SUPPLEMENTARY FIGURE 5 – The average level of non-CpG DNA methylation decreases across fetal brain development ($r = -0.24$, $p = 1.14E-3$).

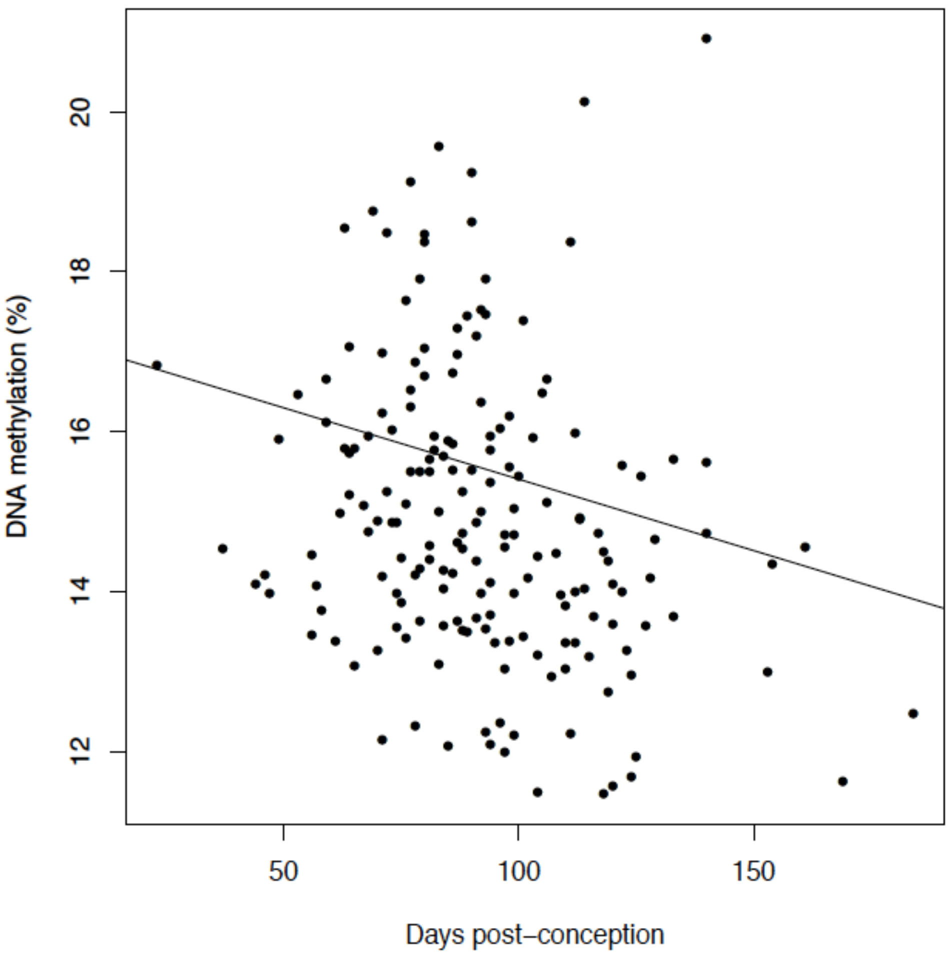

SUPPLEMENTARY FIGURE 6 – The four top-ranked non-CpG sites characterized by changes in DNA methylation across fetal brain development. DNA methylation (%) is plotted against days post-conception. See also **Supplementary Table 5**.

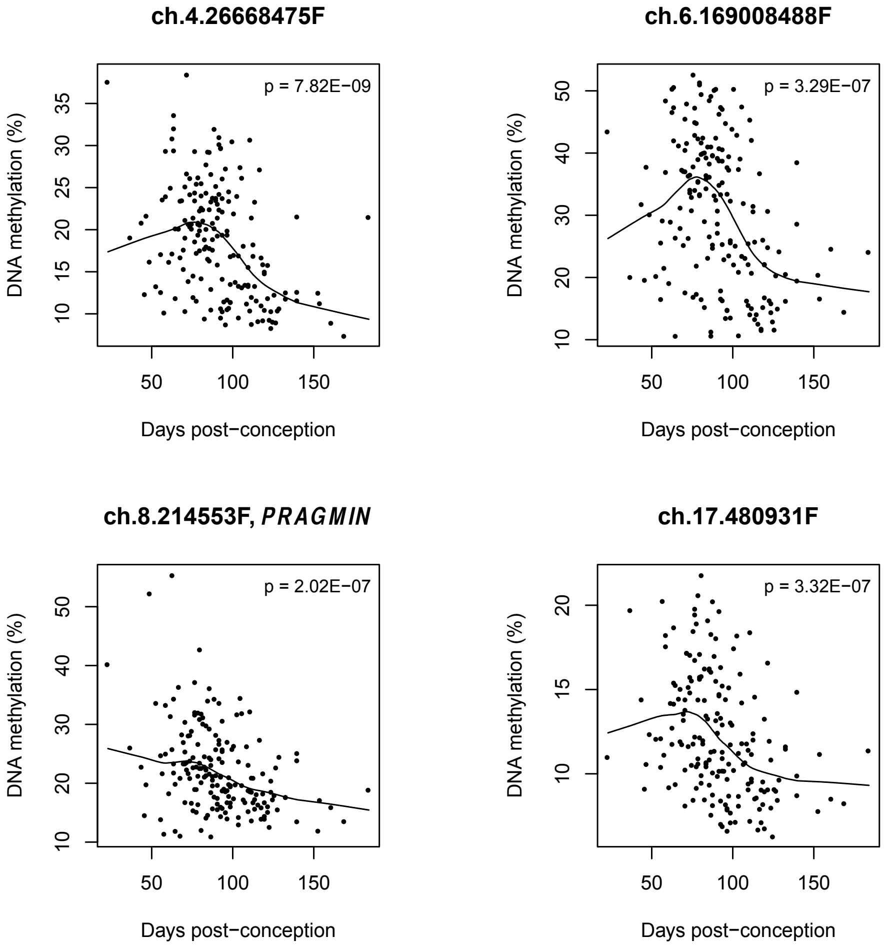

SUPPLEMENTARY FIGURE 7 – (A) Distribution and (B) direction of fetal brain dDMPs across genic features by CpG density (see also **Supplementary Table 6).** Red line represents average percentage of dDMPs across all autosomal probe sites (7.19%).

(A)

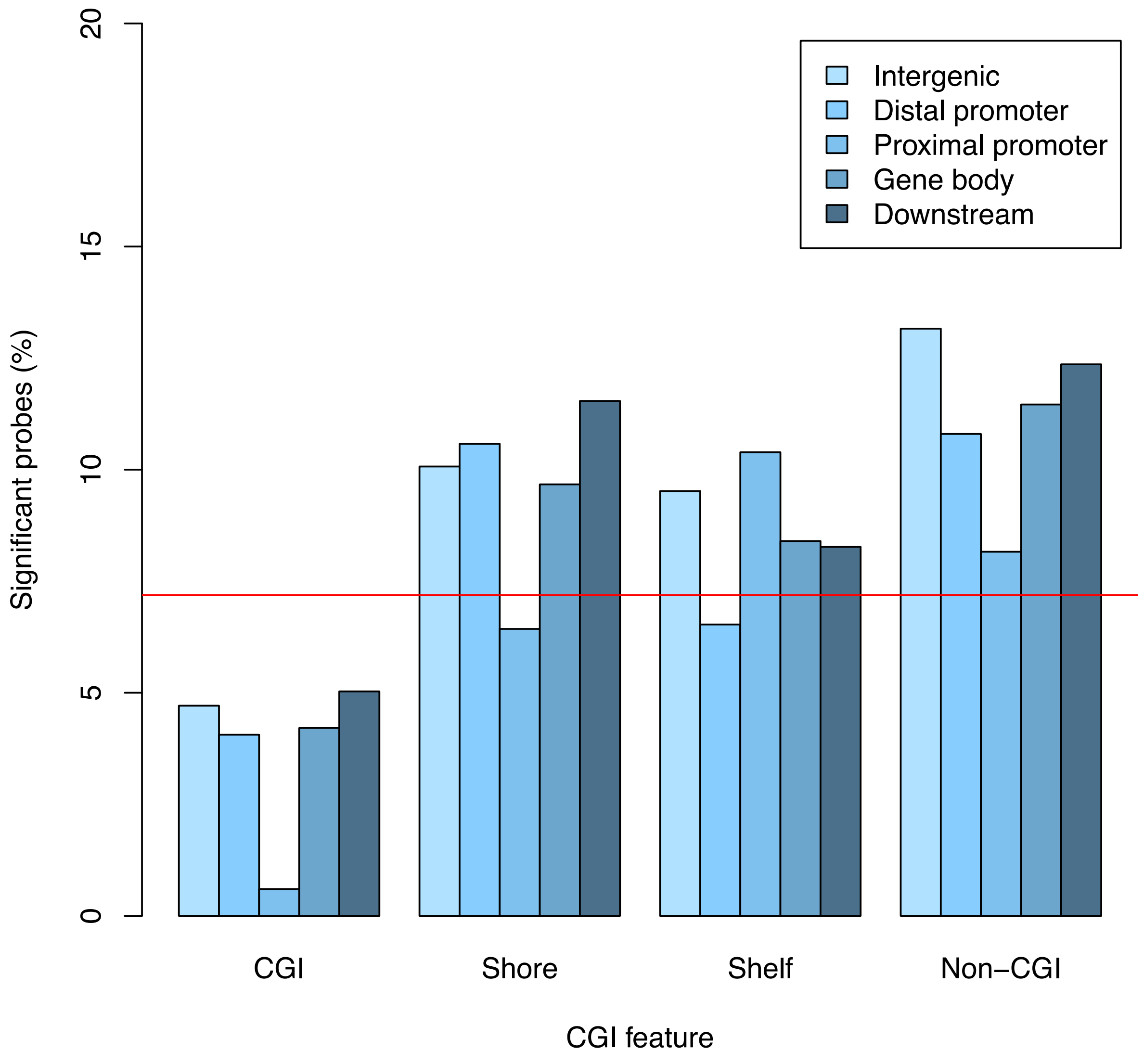

(B)

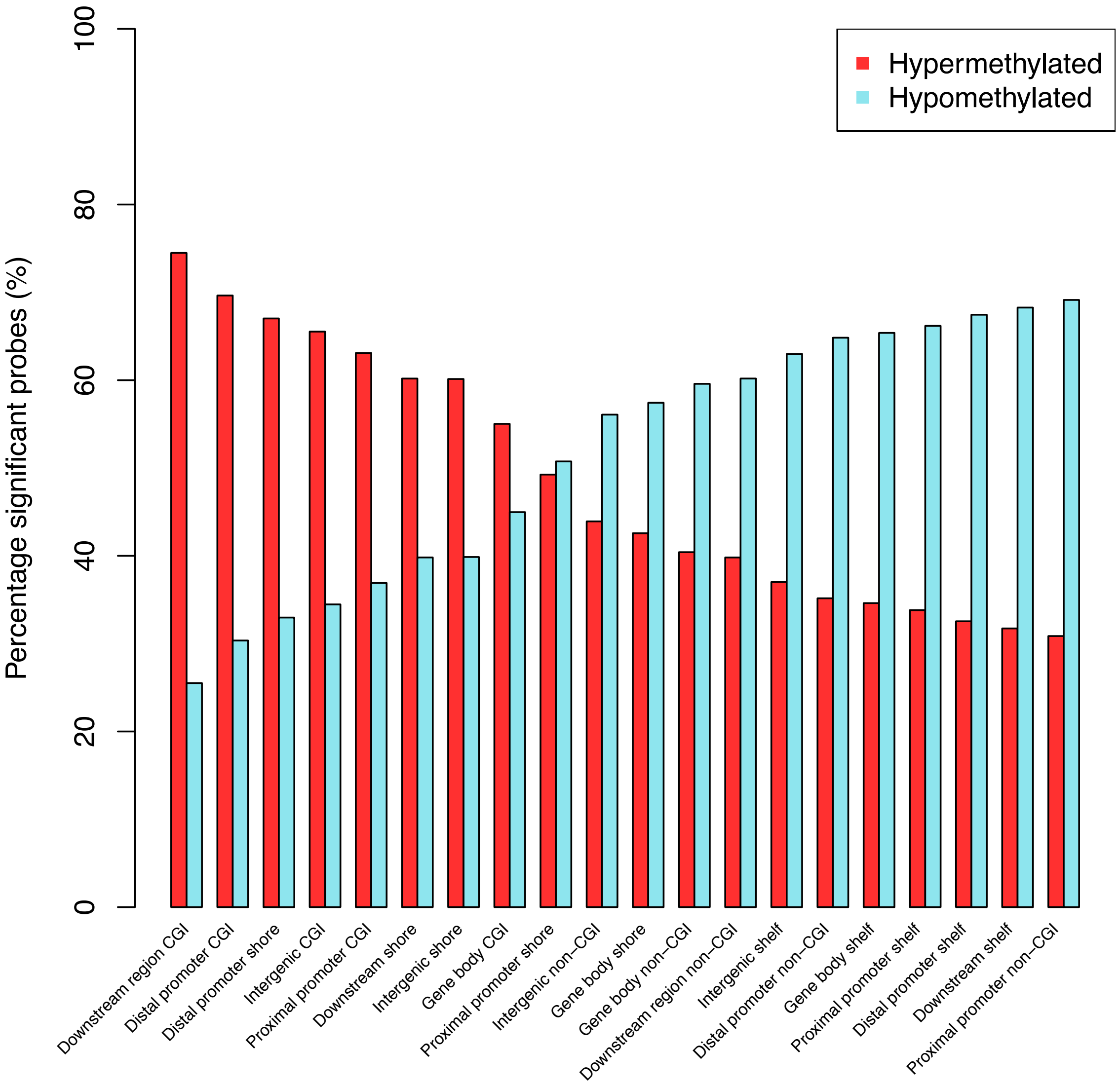
Hypermethylated
Hypomethylated
Percentage significant probes (%)
0
20
40
60
80
100
Downstream region CGI
Distal promoter CGI
Distal promoter shore
Intergenic CGI
Proximal promoter CGI
Downstream shore
Intergenic shore
Gene body CGI
Proximal promoter shore
Intergenic non–CGI
Gene body shore
Gene body non–CGI
Downstream region non–CGI
Intergenic shelf
Distal promoter non–CGI
Gene body shelf
Proximal promoter shelf
Distal promoter shelf
Downstream shelf
Proximal promoter non–CGI

SUPPLEMENTARY FIGURE 8 – (A) Distribution and (B) direction of fetal brain dDMPs associated with alternative transcription events (see also **Supplementary Table 8).** Red line represents average percentage of dDMPs across all autosomal probe sites (7.19%). A3SS = Alternative 3' splice site, A5SS = Alternative 5' splice site, AFE = Alternative first exon, ALE = Alternative last exon, CE = Cassette exon, CNE = Constitutive exon, EI = Exon isoforms, II = Intron isoforms, IR = Intron retention, MXE = Mutually exclusive exon.

(A)

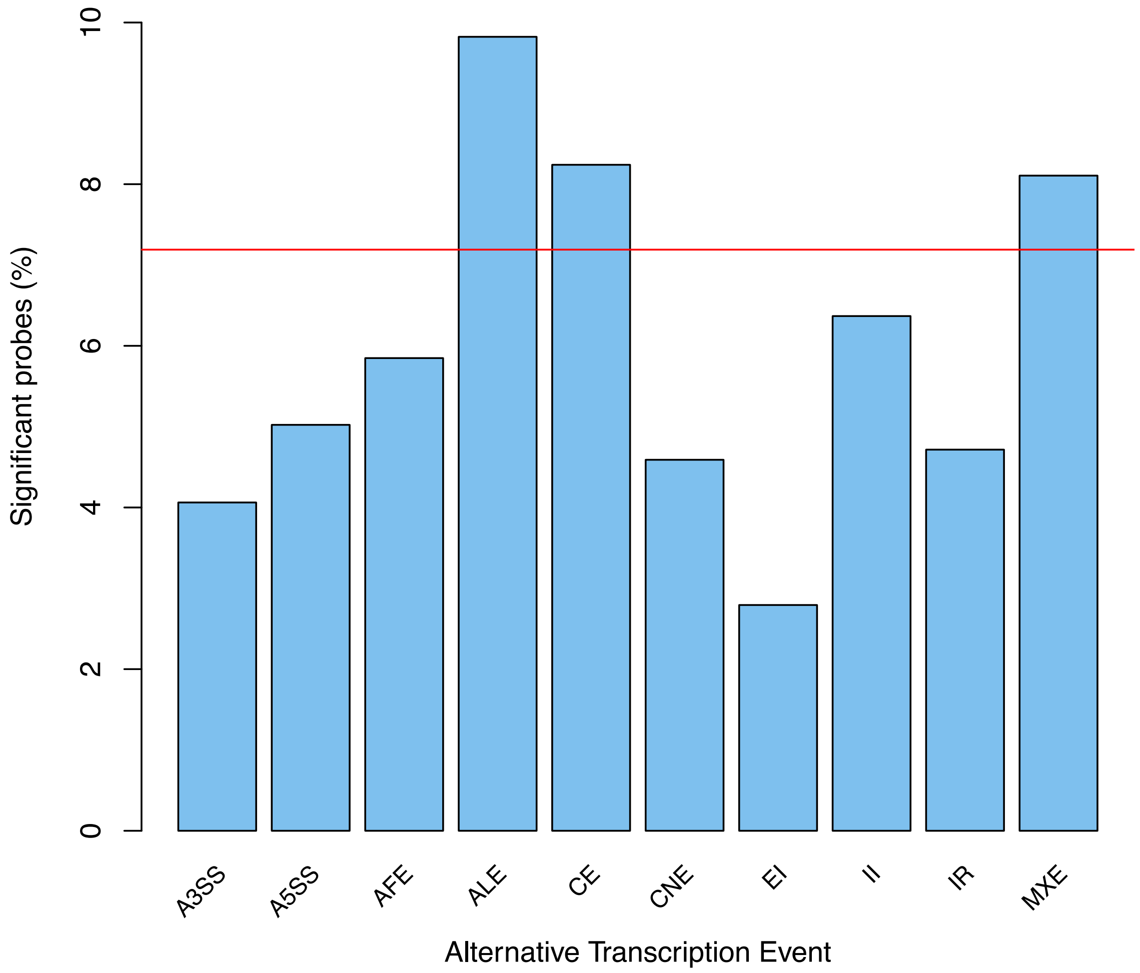

(B)

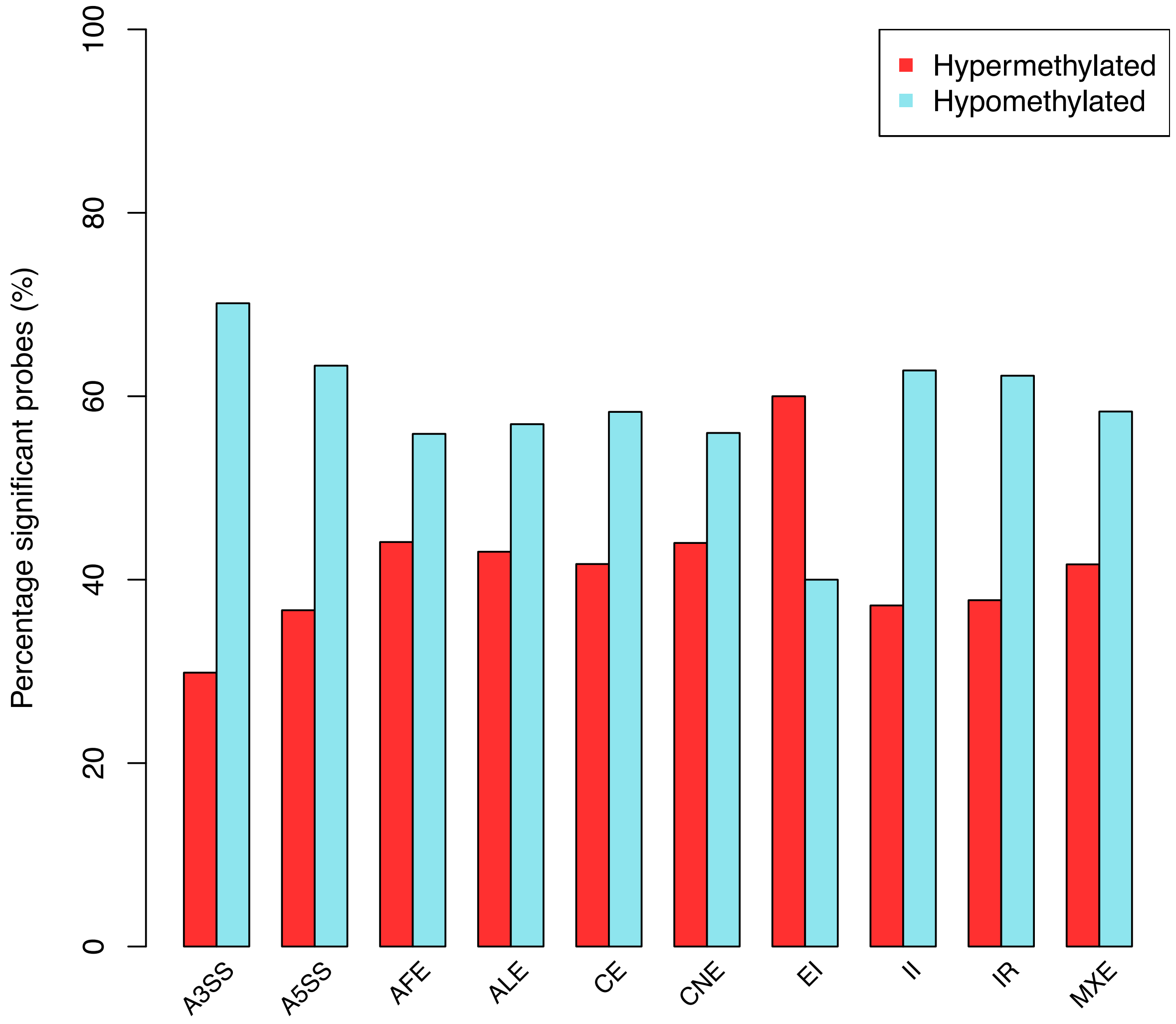

Hypermethylated
Hypomethylated
Percentage significant probes (%)
0
20
40
60
80
100
A3SS
A5SS
AFE
ALE
CE
CNE
EI
II
IR
MXE

SUPPLEMENTARY FIGURE 9 – Manhattan plot of sex-associated CpG sites in the fetal brain (p value corresponds to association with sex). Red line represents Bonferroni corrected threshold. Points above the X-axis indicate hypermethylation in females, and points below the X-axis indicate hypermethylation in males.

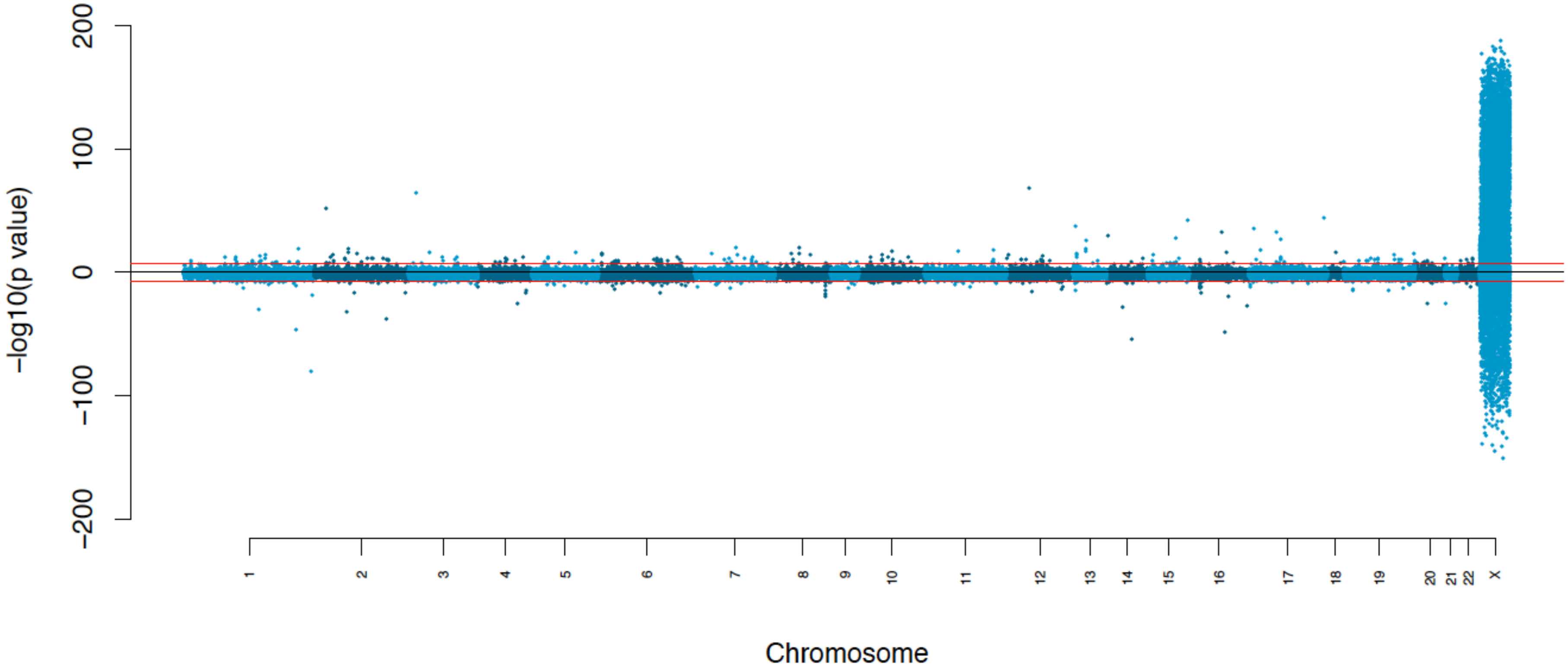

SUPPLEMENTARY FIGURE 10 – Significant correlation (r = 0.88, p <1E-200) between DNA methylation differences (female minus male) at autosomal sex-associated probes (n = 544) in the adult cortex (from Xu et al. 2014) and differences observed at the same loci in fetal cortex. These data indicate that stable autosomal differences in DNA methylation between males and females become manifest early in fetal development.

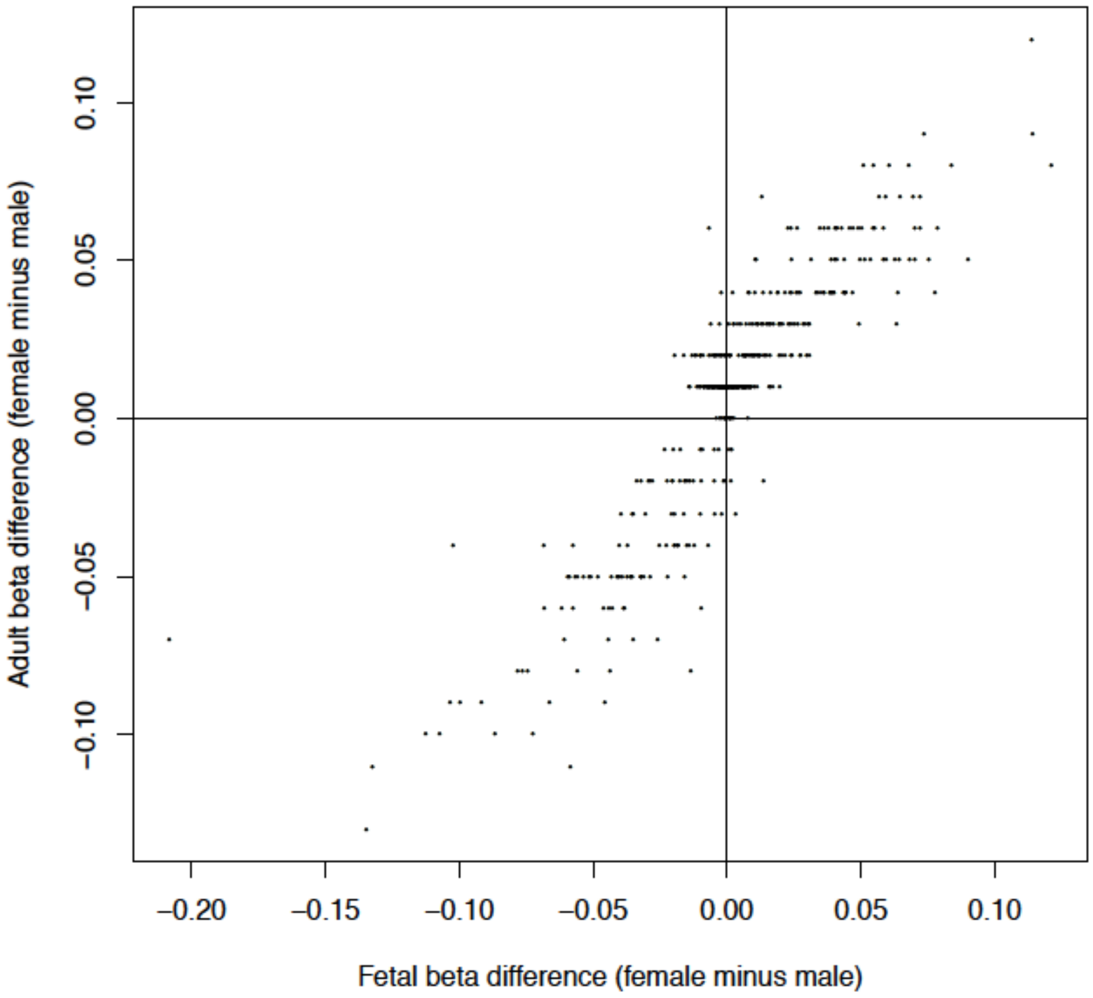

SUPPLEMENTARY FIGURE 11 – The top-ranked autosomal sex-associated DMR spans 103bp (Chr13: 49796387 – 49796490) **within the *MLNR* gene**. See **Supplementary Table 10** for a list of top-ranked autosomal sex-associated DMRs. A full list of sex-associated DMRs is available for download from our laboratory website - http://epigenetics.iop.kcl.ac.uk/fetalbrain/1099_sex_DMRs.csv. The sex-associated DMR is denoted by dashed lines. Chromosomal coordinates correspond to human genome build Feb. 2009 (GRCh37/hg19).

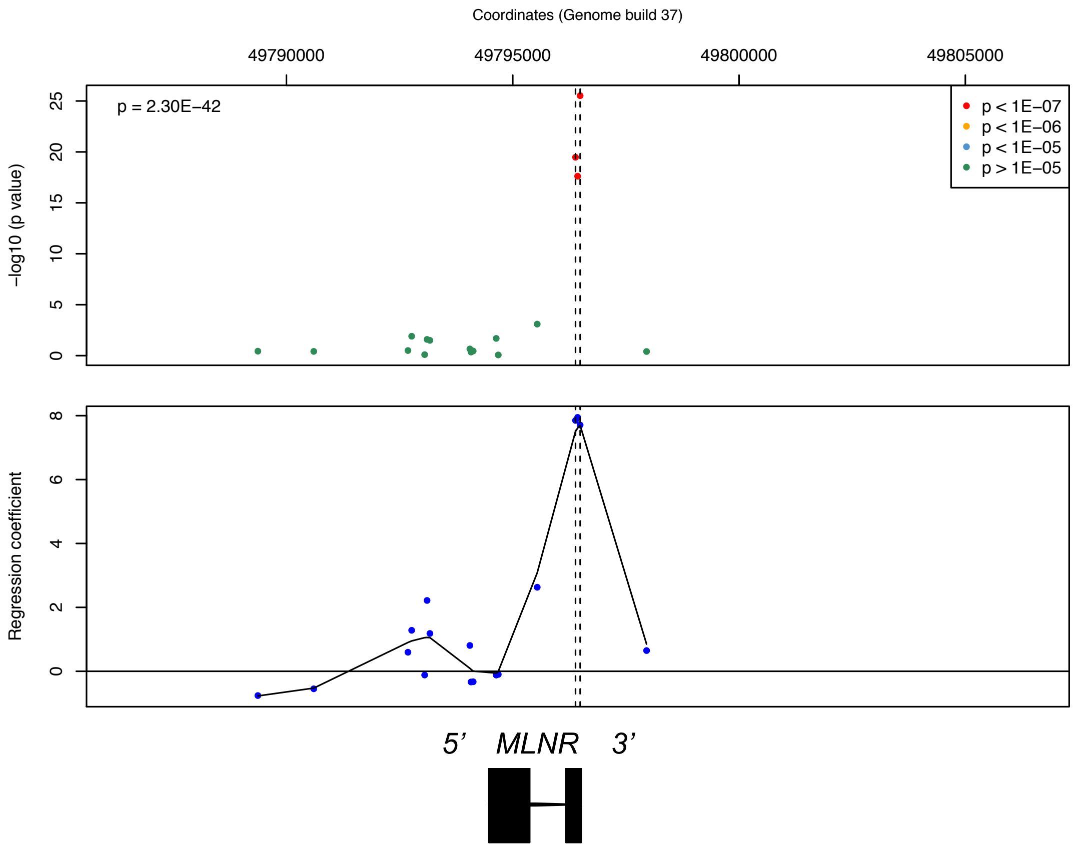

SUPPLEMENTARY FIGURE 12 – Manhattan plot depicting the location of sex-specific dDMPs (p value corresponds to association with age and sex). Red line represents a Bonferroni corrected threshold of (-log10) 1.22E-7.

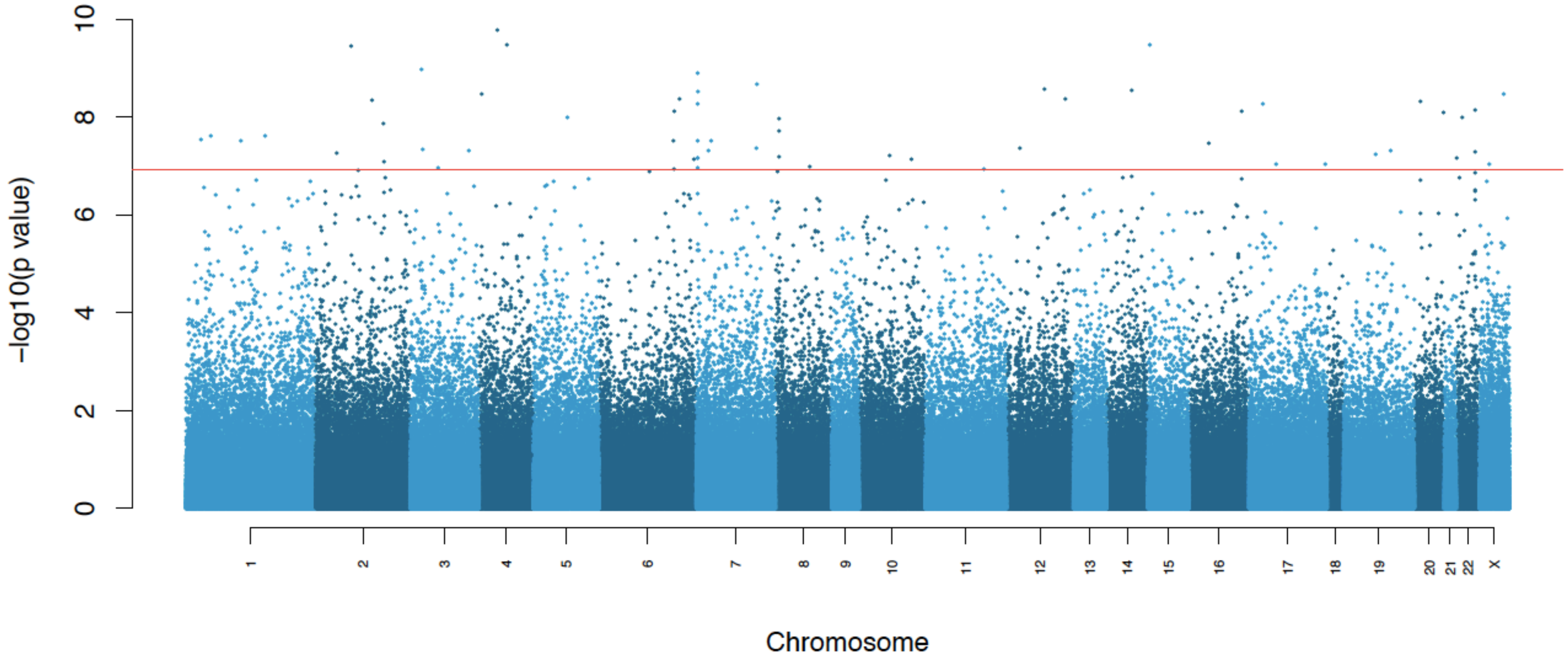

SUPPLEMENTARY FIGURE 13 – The top-ranked sex-specific dDMRs span two regions (Chr7:640338 - 640821, 483bp, p = 5.19E-16, denoted by dark blue dashed lines and Chr7:632926 - 633203, 277bp, p = 2.45E-12, denoted by light blue dashed lines) within the *PRKAR1B* gene. See **Supplementary Table 11** for a list of top-ranked autosomal sex-associated DMRs Chromosomal coordinates correspond to human genome build Feb. 2009 (GRCh37/hg19).

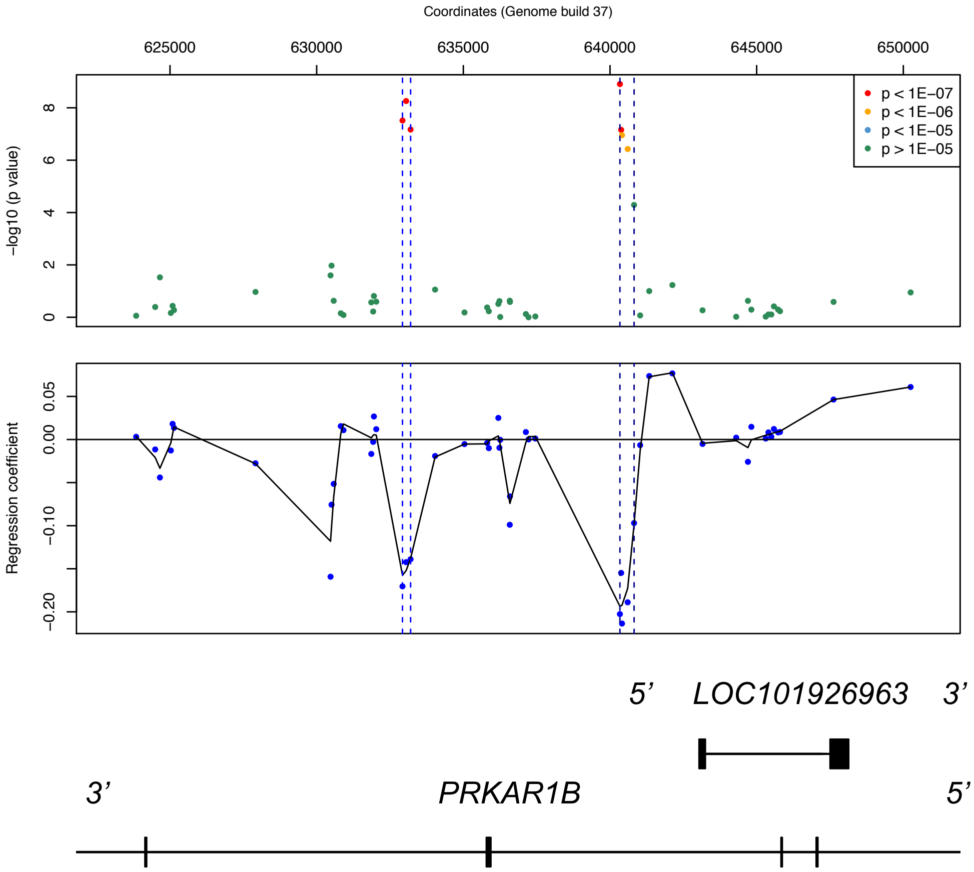

SUPPLEMENTARY FIGURE 14 – Relationship between module eigengene and fetal brain development. Panels are ordered by decreasing correlation with DPC from top-left to bottom-right. See also **Figure 4b**.

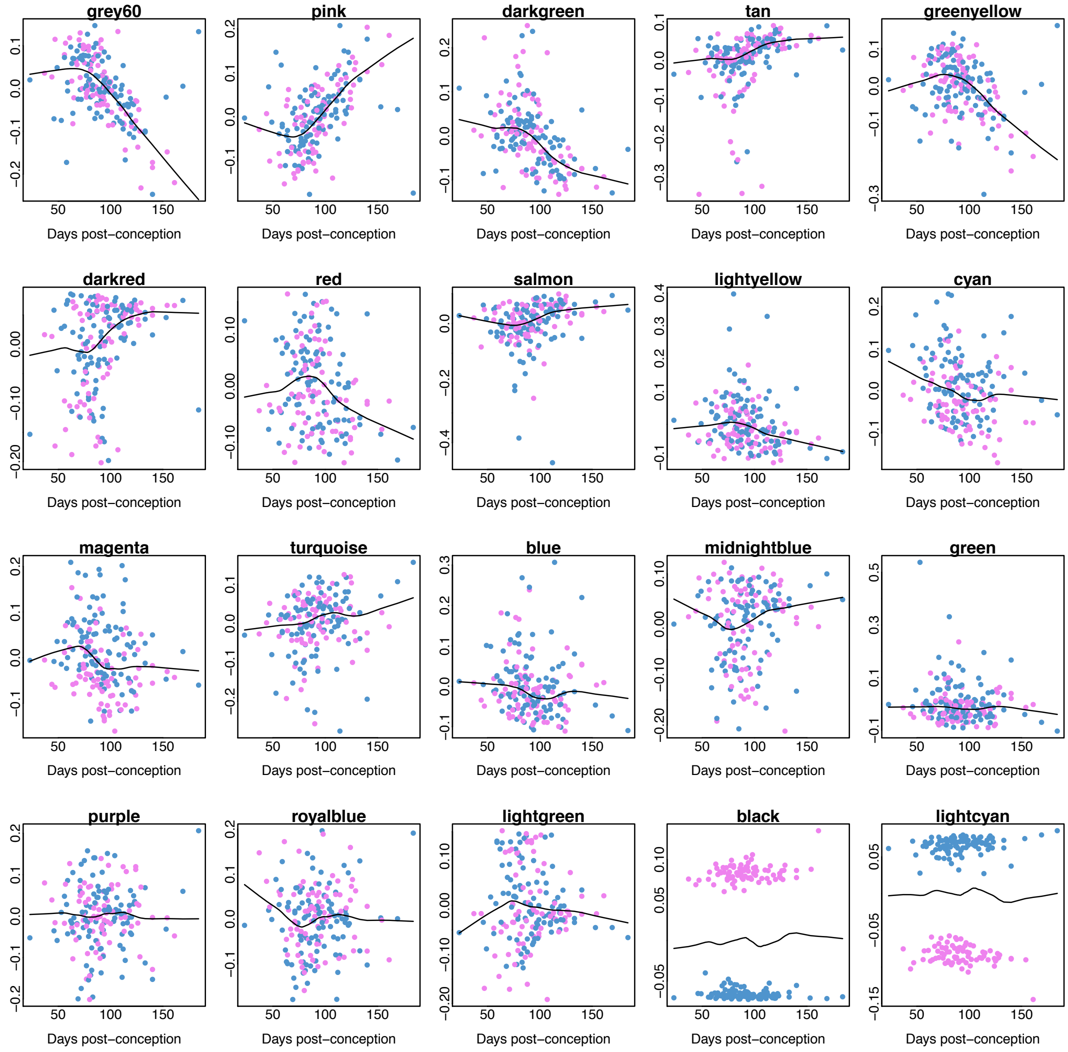

Supplement: Supplemental Material [file supp_gr.180273.114_Supplemental_Figures.pdf]
