# Supplementary material for: Methylomic trajectories across human fetal brain development
Source: Genome Res. 2015 Mar;25(3):338–52. doi: 10.1101/gr.180273.114 (PMC4352878; doi:10.1101/gr.180273.114)
Supplement: Supplemental Material [file supp_gr.180273.114_Supplemental_Tables.docx]

**SUPPLEMENTARY TABLE 1 – Distribution of dDMPs significantly associated with human brain development split by autosomal chromosome and direction of effect.** Bonferroni correction (p < 1.25E-7) was used as a threshold to determine significance. See also **Figure 1b**.

| **Chromosome** | **Total probes** | **Significant**  **(p < 1.25E-7) probes (%)** | **Enrichment**  **(95% CI)** | **p value** | **Hypermethylated**  **(%)** | **Hypomethylated**  **(%)** | **Proportion hypermethylated**  **(95% CI)** | **Enrichment p value** | |
| --- | --- | --- | --- | --- | --- | --- | --- | --- | --- |
| 1 | 40079 | 3109 (7.76) | 1.09 (1.04 -1.13) | 3.56E-05 | 1375 (44.23) | 1734 (55.77) | 0.79 (0.72 -0.88) | | 5.40E-06 |
| 2 | 29186 | 2310 (7.91) | 1.11 (1.06 -1.16) | 5.32E-06 | 1133 (49.05) | 1177 (50.95) | 0.96 (0.86 -1.08) | | 5.37E-01 |
| 3 | 21158 | 1532 (7.24) | 1.01 (0.95 -1.06) | 7.85E-01 | 675 (44.06) | 857 (55.94) | 0.79 (0.68 -0.91) | | 1.12E-03 |
| 4 | 16866 | 1143 (6.78) | 0.94 (0.88 -1.00) | 4.12E-02 | 513 (44.88) | 630 (55.12) | 0.81 (0.69 -0.96) | | 1.51E-02 |
| 5 | 20246 | 1427 (7.05) | 0.98 (0.93 -1.03) | 4.51E-01 | 596 (41.77) | 831 (58.23) | 0.72 (0.62 -0.83) | | 1.10E-05 |
| 6 | 30026 | 1896 (6.31) | 0.87 (0.83 -0.91) | 7.97E-09 | 834 (43.99) | 1062 (56.01) | 0.79 (0.69 -0.89) | | 2.35E-04 |
| 7 | 24385 | 1947 (7.98) | 1.12 (1.07 -1.17) | 4.57E-06 | 873 (44.84) | 1074 (55.16) | 0.81 (0.72 -0.92) | | 1.33E-03 |
| 8 | 17438 | 1407 (8.07) | 1.13 (1.07 -1.20) | 1.57E-05 | 669 (47.55) | 738 (52.45) | 0.91 (0.78 -1.05) | | 2.00E-01 |
| 9 | 8416 | 746 (8.86) | 1.26 (1.16 -1.35) | 1.31E-08 | 330 (44.24) | 416 (55.76) | 0.79 (0.64 -0.98) | | 2.93E-02 |
| 10 | 20511 | 1620 (7.90) | 1.11 (1.05 -1.17) | 1.60E-04 | 801 (49.44) | 819 (50.56) | 0.98 (0.85 -1.13) | | 7.79E-01 |
| 11 | 25024 | 1850 (7.39) | 1.03 (0.98 -1.08) | 2.31E-01 | 788 (42.59) | 1062 (57.41) | 0.74 (0.65 -0.85) | | 7.26E-06 |
| 12 | 20720 | 1428 (6.89) | 0.96 (0.90 -1.01) | 1.06E-01 | 610 (42.72) | 818 (57.28) | 0.75 (0.64 -0.87) | | 1.10E-04 |
| 13 | 10168 | 902 (8.87) | 1.26 (1.17 -1.35) | 3.68E-10 | 492 (54.55) | 410 (45.45) | 1.20 (0.99 -1.45) | | 5.93E-02 |
| 14 | 12746 | 939 (7.37) | 1.03 (0.96 -1.10) | 4.44E-01 | 369 (39.30) | 570 (60.70) | 0.65 (0.54 -0.78) | | 3.39E-06 |
| 15 | 12681 | 938 (7.40) | 1.03 (0.96 -1.10) | 3.73E-01 | 433 (46.16) | 505 (53.84) | 0.86 (0.71 -1.03) | | 1.06E-01 |
| 16 | 18523 | 1330 (7.18) | 1.00 (0.94 -1.06) | 9.77E-01 | 499 (37.52) | 831 (62.48) | 0.60 (0.51 -0.70) | | 1.06E-10 |
| 17 | 24067 | 1763 (7.33) | 1.02 (0.97 -1.07) | 4.33E-01 | 661 (37.49) | 1102 (62.51) | 0.60 (0.52 -0.69) | | 7.51E-14 |
| 18 | 5113 | 294 (5.75) | 0.79 (0.70 -0.89) | 4.77E-05 | 153 (52.04) | 141 (47.96) | 1.08 (0.78 -1.52) | | 6.80E-01 |
| 19 | 21883 | 894 (4.09) | 0.55 (0.51 -0.59) | 7.01E-79 | 244 (27.29) | 650 (72.71) | 0.38 (0.31 -0.46) | | 5.90E-23 |
| 20 | 9254 | 526 (5.68) | 0.78 (0.71 -0.85) | 1.09E-08 | 223 (42.40) | 303 (57.60) | 0.74 (0.57 -0.95) | | 1.58E-02 |
| 21 | 3663 | 208 (5.68) | 0.78 (0.67 -0.89) | 3.12E-04 | 76 (36.54) | 132 (63.46) | 0.58 (0.38 -0.87) | | 7.46E-03 |
| 22 | 7211 | 509 (7.06) | 0.98 (0.89 -1.07) | 6.96E-01 | 181 (35.56) | 328 (64.44) | 0.55 (0.43 -0.72) | | 3.62E-06 |
| Total | 399364 | 28718 (7.19) | NA | NA | 12528 (43.6) | 16190 (56.4) | 0.77 (0.75 - 0.80) | | 6.74E-53 |

**SUPPLEMENTARY TABLE 2 - Association between gene expression and brain development for top-ranked dDMPs.** Fetal brain microarray gene expression data for loci annotated to the top 20 hypermethylated and top 20 hypomethylated dDMPs (listed in **Table 1**) was extracted from Brain Cloud (<http://braincloud.jhmi.edu>) (Colantuoni et al. 2011). Gene corresponds to the official gene symbol corresponding to the probe. Clone Index refers to the ID of the Illumina oligonucleotide probe. Gene position corresponds to human genome build Mar 2006 (NCB136/hg18). dDMP direction corresponds to the relationship between DNA methylation and age; “hyper” refers to a positive correlation, “hypo” refers to a negative correlation. See also **Supplementary Figure 3**.

| **Gene (Clone Index)** | **Accession** | **Gene description** | **Gene position** | **Regression coefficient** | **p value** | **dDMP direction** |
| --- | --- | --- | --- | --- | --- | --- |
| *BTG3* | NM_001130914 | BTG family, member 3 | Chr21:17887840-17907139 | -1.03E+01 | 2.49E-06 | hypo |
| *KLHL35* | NM_001039548 | Kelch-like 35 (Drosophila) | Chr11:74811085-74818881 | 9.35E+00 | 2.08E-05 | hyper |
| *FAM49A* (7311) | AK091280 | Family with sequence similarity 49, member A | Chr2:16594766-16606855 | 7.73E+00 | 8.68E-05 | hypo |
| *WIPF1* | NM_001077269 | WAS/WASL interacting protein family, member 1 | Chr2:175132547-175255873 | -7.07E+00 | 3.75E-03 | hypo |
| *VCL* (5369) | NM_014000 | Vinculin | Chr10:75427877-75549920 | -5.54E+00 | 8.90E-03 | hypo |
| *NFIC* (35404) | NM_205843 | Nuclear factor I/C (CCAAT-binding transcription factor) | Chr19:3310615-3414603 | -9.60E+00 | 9.88E-03 | hyper |
| *FAM49A* (1692) | NM_030797 | Family with sequence similarity 49, member A | Chr2:16597381-16710577 | 7.50E+00 | 1.91E-02 | hypo |
| *NFIC* (25857) | NM_205843 | Nuclear factor I/C (CCAAT-binding transcription factor) | Chr19:3310615-3414603 | -5.58E+00 | 1.95E-02 | hyper |
| *EBF4* | NM_001110514 | Early B-cell factor 4 | Chr20:2621523-2688754 | 3.44E+00 | 2.05E-02 | hyper |
| *TTYH3* (23255) | NM_025250 | Tweety homolog 3 (Drosophila) | Chr7:2638128-2670962 | 3.45E+00 | 5.51E-02 | hypo |
| *EMILIN3* | NM_052846 | Elastin microfibril interfacer 3 | Chr20:39422019-39428912 | -3.40E+00 | 6.53E-02 | hyper |
| *PHKG1* (26747) | NM_006213 | Phosphorylase kinase, gamma 1 (muscle) | Chr7:56116168-56128183 | -5.46E+00 | 1.29E-01 | hypo |
| *HPS4* (38999) | NM_022081 | Hermansky-Pudlak syndrome 4 | Chr22:25177445-25209820 | 1.18E+00 | 2.25E-01 | hypo |
| *NPHS2* | NM_014625 | Nephrosis 2, idiopathic, steroid-resistant (podocin) | Chr1:177786296-177811707 | -2.82E+00 | 2.37E-01 | hyper |
| *SLC44A4* | NM_025257 | Solute carrier family 44, member 4 | Chr6:31938948-31954802 | -2.06E+00 | 3.16E-01 | hypo |
| *NFIC* (39741) | NM_205843 | Nuclear factor I/C (CCAAT-binding transcription factor) | Chr19:3310615-3414603 | -9.37E-01 | 3.46E-01 | hyper |
| *ZNF251* | NM_138367 | Zinc finger protein 251 | Chr8:145917102-145951775 | 2.16E+00 | 4.17E-01 | hypo |
| *CHSY1* | NM_014918 | Chondroitin sulfate synthase 1 | Chr15:99533454-99609649 | 9.78E-01 | 4.92E-01 | hypo |
| *TTYH3* (11389) | NM_025250 | Tweety homolog 3 (Drosophila) | Chr7:2638128-2670962 | -1.02E+00 | 5.71E-01 | hypo |
| *HPS4* (9256) | CF143296 | Hermansky-Pudlak syndrome 4 | Chr22:25196588-25196980 | -7.81E-01 | 5.89E-01 | hypo |
| *GUCY2C* (36631) | NM_004963 | Guanylate cyclase 2C (heat stable enterotoxin receptor) | Chr12:14656835-14740786 | -1.06E+00 | 7.35E-01 | hypo |
| *GUCY2C* (22186) | NM_004963 | Guanylate cyclase 2C (heat stable enterotoxin receptor) | Chr12:14656835-14740786 | -2.86E-01 | 7.91E-01 | hypo |
| *STEAP2* | NM_152999 | Six transmembrane epithelial antigen of the prostate 2 | Chr7:89678935-89704928 | -1.20E+00 | 8.16E-01 | hyper |
| *PHKG1* (29456) | BJ996346 | EST | Chr7:56046939-56151451 | -3.33E-01 | 8.22E-01 | hypo |
| *CDX1* | NM_001804 | Caudal type homeobox 1 | Chr5:149526536-149544314 | -1.23E-01 | 8.54E-01 | hyper |
| *PLEKHG3* (18517) | NM_015549 | Pleckstrin homology domain containing, family G (with RhoGef domain) member 3 | Chr14:64240945-64280813 | 4.21E-01 | 8.83E-01 | hyper |
| *GRHL2* | NM_024915 | Grainyhead-like 2 (Drosophila) | Chr8:102573843-102751128 | -1.41E-01 | 9.16E-01 | hyper |
| *PACS2* | NM_001100913 | Phosphofurin acidic cluster sorting protein 2 | Chr14:104852125-104935529 | -7.95E-02 | 9.58E-01 | hypo |
| *PHKG1* (20639) | AK300928 | Phosphorylase kinase, gamma 1 (muscle) | Chr7:56115786-56128135 | -6.31E-02 | 9.71E-01 | hypo |
| *VCL* (37372) | NM_014000 | Vinculin | Chr10:75427877-75549920 | -4.54E-02 | 9.87E-01 | hypo |
| *PLEKHG3* (21344) | AB011171 | Pleckstrin homology domain containing, family G (with RhoGef domain) member 3 | Chr14:64277554-64283363 | -2.92E-02 | 9.91E-01 | hyper |

**SUPPLEMENTARY TABLE 3 - Summary of differentially methylated regions (DMRs) associated with fetal brain development, sex, and development-sex interactions by chromosome.** Spatially-correlated regions (Sidak corrected p < 0.05, number of probes ≥ 3) of differential DNA methylation were identified with *comb-p* (using the parameters: seed = E-4; distance = 500).

|  | **Age association** | **Sex association** | **Age-sex interaction** |
| --- | --- | --- | --- |
| **Regions (n)** | 4825 | 1099 | 66 |
| **Mean probes per region (n)** | 4.77 | 6.06 | 4.91 |
| **Mean region size (bp) (SD)** | 391 (250) | 472 (271) | 372 (194) |
| **Number of regions by chromosome:** |  |  |  |
| **1** | 445 | 17 | 7 |
| **2** | 316 | 13 | 7 |
| **3** | 178 | 2 | 2 |
| **4** | 182 | 7 | 3 |
| **5** | 190 | 8 | 3 |
| **6** | 332 | 21 | 3 |
| **7** | 402 | 16 | 8 |
| **8** | 248 | 11 | 3 |
| **9** | 42 | 3 | 0 |
| **10** | 358 | 10 | 2 |
| **11** | 336 | 8 | 5 |
| **12** | 238 | 8 | 2 |
| **13** | 182 | 5 | 2 |
| **14** | 152 | 3 | 1 |
| **15** | 138 | 4 | 3 |
| **16** | 254 | 10 | 2 |
| **17** | 375 | 10 | 4 |
| **18** | 52 | 0 | 0 |
| **19** | 184 | 19 | 4 |
| **20** | 83 | 4 | 1 |
| **21** | 27 | 6 | 0 |
| **22** | 78 | 6 | 3 |
| **X** | 33 | 908 | 1 |

**SUPPLEMENTARY TABLE 4 – The twenty top-ranked dDMRs associated with fetal brain development.** Chromosomal coordinates correspond to human genome build Feb. 2009 (GRCh37/hg19). A complete list of all significant dDMRs is available for download from our laboratory website (<http://epigenetics.iop.kcl.ac.uk/fetalbrain/4825_age_DMRs.csv>). **See also Supplementary Figure 4**.

| **Chr** | **Start** | **End** | **Size (bp)** | **Probes (n)** | **p value** | **Gene** |
| --- | --- | --- | --- | --- | --- | --- |
| 8 | 41167660 | 41168482 | 822 | 12 | 4.86E-69 | *SFRP1* |
| 8 | 65291287 | 65292499 | 1212 | 14 | 1.38E-65 | *MIR124-2* |
| 1 | 10509682 | 10510500 | 818 | 9 | 3.77E-64 | *APITD1; CORT* |
| 5 | 149545907 | 149546947 | 1040 | 12 | 5.48E-63 | *CDX1* |
| 16 | 89118603 | 89119710 | 1107 | 8 | 3.09E-62 | *-* |
| 22 | 24979254 | 24979965 | 711 | 11 | 1.63E-60 | *GGT1* |
| 2 | 157184137 | 157184979 | 842 | 9 | 4.82E-58 | *NR4A2* |
| 7 | 100463206 | 100464146 | 940 | 7 | 1.56E-57 | *SLC12A9; TRIP6* |
| 5 | 134827136 | 134828037 | 901 | 7 | 1.84E-57 | *-* |
| 7 | 2653651 | 2654531 | 880 | 8 | 2.73E-57 | *IQCE* |
| 13 | 100642408 | 100643389 | 981 | 8 | 1.51E-56 | *-* |
| 13 | 100643858 | 100644658 | 800 | 8 | 1.60E-56 | *-* |
| 12 | 89748726 | 89749378 | 652 | 9 | 2.81E-56 | *-* |
| 13 | 95357638 | 95358152 | 514 | 6 | 5.20E-55 | *-* |
| 6 | 30720080 | 30720492 | 412 | 8 | 5.61E-55 | *-* |
| 20 | 19866743 | 19867146 | 403 | 8 | 1.62E-54 | *-* |
| 11 | 70557445 | 70557882 | 437 | 6 | 4.31E-54 | *SHANK2* |
| 17 | 42992658 | 42993051 | 393 | 7 | 4.92E-54 | *GFAP* |
| 13 | 100641243 | 100642107 | 864 | 9 | 1.63E-53 | *-* |
| 22 | 26875499 | 26876076 | 577 | 7 | 9.21E-53 | *HPS4* |

**SUPPLEMENTARY TABLE 5 – DNA methylation at fourteen non-CpG sites is significantly associated with fetal brain development.** Chromosomal coordinates correspond to human genome build Feb. 2009 (GRCh37/hg19). Regression coefficient = DNA methylation change (%) per day development. Bonferroni corrected p value threshold of p < 6.73E-05. See also **Supplementary Figure 6**.

| **Probe** | **Chr** | **Position** | **Gene** | **Genic Probe Location** | **Regression Coefficient** | **DNA methylation (%) mean (SD)** | **p value** |
| --- | --- | --- | --- | --- | --- | --- | --- |
| ch.4.26668475F | 4 | 27059377 |  |  | -0.11 | 18.77 (6.69) | 7.82E-09 |
| ch.8.214553F | 8 | 8189620 | *PRAGMIN* | Body | -0.11 | 22.24 (7.21) | 2.02E-07 |
| ch.6.169008488F | 6 | 169266563 |  |  | -0.18 | 30.70 (11.59) | 3.29E-07 |
| ch.17.480931F | 17 | 14532155 |  |  | -0.05 | 12.30 (3.59) | 3.32E-07 |
| ch.14.96436874F | 14 | 97367121 |  |  | -0.13 | 28.57 (8.39) | 3.92E-07 |
| ch.2.16090152F | 2 | 16172701 |  |  | -0.15 | 38.96 (10.32) | 6.82E-07 |
| ch.15.75593923F | 15 | 77806868 |  |  | -0.08 | 14.36 (5.55) | 7.50E-07 |
| ch.6.1239582F | 6 | 52412686 | *TRAM2* | Body | -0.05 | 17.71 (3.86) | 2.63E-06 |
| ch.22.533187F | 22 | 35783418 | *HMOX1* | Body | -0.03 | 9.72 (2.41) | 1.93E-05 |
| ch.7.87722890F | 7 | 87884954 |  |  | -0.03 | 11.72 (2.51) | 3.11E-05 |
| ch.16.1630026F | 16 | 72492804 |  |  | -0.08 | 22.24 (6.43) | 4.42E-05 |
| ch.4.134210136F | 4 | 133990686 |  |  | -0.02 | 7.49 (1.90) | 5.14E-05 |
| ch.4.6573835F | 4 | 6522934 |  |  | -0.06 | 14.39 (5.36) | 5.33E-05 |
| ch.19.50335620F | 19 | 45643780 |  |  | -0.05 | 10.52 (4.48) | 5.83E-05 |

**SUPPLEMENTARY TABLE 6– Characterization of dDMPs in genic features stratified by CpG density.** Bonferroni correction (p < 1.25E-7) was used as a threshold to determine significance.

|  | **Total probes** | **Significant**  **(p < 1.25E-7) probes (%)** | **Enrichment**  **(95% CI)** | **p value** | **Hypermethylated**  **(%)** | **Hypomethylated**  **(%)** | **Enrichment hypermethylated**  **(95% CI)** | **p value** |
| --- | --- | --- | --- | --- | --- | --- | --- | --- |
| *Intergenic non-CGI* | 34015 | 4476 (13.16) | 1.96 (1.89 - 2.02) | 3.92E-294 | 1966 (43.92) | 2510 (56.08) | 0.78 (0.72 - 0.85) | 9.42E-09 |
| *Intergenic CGI* | 9969 | 470 (4.71) | 0.64 (0.58 - 0.70) | 1.09E-23 | 308 (65.53) | 162 (34.47) | 1.90 (1.45- 2.49) | 1.89E-06 |
| *Intergenic shelf* | 4568 | 435 (9.52) | 1.36 (1.23 - 1.50) | 6.00E-09 | 161 (37.01) | 274 (62.99) | 0.59 (0.44- 0.78) | 1.26E-04 |
| *Intergenic shore* | 8915 | 898 (10.07) | 1.45 (1.35 - 1.55) | 6.66E-23 | 540 (60.13) | 358 (39.87) | 1.51 (1.25- 1.83) | 1.93E-05 |
| *Distal promoter non-CGI* | 3371 | 364 (10.80) | 1.56 (1.40 - 1.74) | 4.35E-14 | 128 (35.16) | 236 (64.84) | 0.54 (0.40- 0.74) | 6.89E-05 |
| *Distal promoter CGI* | 4139 | 168 (4.06) | 0.55 (0.46 - 0.64) | 5.85E-17 | 117 (69.64) | 51 (30.36) | 2.29 (1.43- 3.68) | 3.53E-04 |
| *Distal promoter shelf* | 6491 | 424 (6.53) | 0.90 (0.81 - 1.00) | 4.18E-02 | 138 (32.55) | 286 (67.45) | 0.48 (0.36- 0.64) | 3.29E-07 |
| *Distal promoter shore* | 4358 | 461 (10.58) | 1.53 (1.38 - 1.68) | 6.06E-16 | 309 (67.03) | 152 (32.97) | 2.03 (1.54- 2.68) | 1.73E-07 |
| *Proximal promoter non-CGI* | 28469 | 2323 (8.16) | 1.15 (1.10 - 1.20) | 2.17E-09 | 717 (30.87) | 1606 (69.13) | 0.45 (0.40- 0.50) | 1.74E-40 |
| *Proximal promoter CGI* | 83400 | 504 (0.60) | 0.08 (0.07 - 0.09) | <3.92E-294 | 318 (63.10) | 186 (36.90) | 1.71 (1.32- 2.22) | 3.53E-05 |
| *Proximal promoter shelf* | 3331 | 346 (10.39) | 1.50 (1.33 - 1.67) | 1.93E-11 | 117 (33.82) | 229 (66.18) | 0.51 (0.37- 0.70) | 2.17E-05 |
| *Proximal promoter shore* | 44672 | 2873 (6.43) | 0.89 (0.85 - 0.92) | 2.12E-09 | 1415 (49.25) | 1458 (50.75) | 0.97 (0.87- 1.08) | 5.80E-01 |
| *Gene body non-CGI* | 67790 | 7772 (11.46) | 1.67 (1.63 - 1.72) | 4.89E-291 | 3141 (40.41) | 4631 (59.59) | 0.68 (0.64- 0.72) | 3.56E-33 |
| *Gene body CGI* | 33056 | 1392 (4.21) | 0.57 (0.54 - 0.60) | 9.53E-106 | 766 (55.03) | 626 (44.97) | 1.22 (1.05- 1.42) | 8.81E-03 |
| *Gene body shelf* | 20421 | 1716 (8.40) | 1.18 (1.12 - 1.25) | 1.77E-10 | 594 (34.62) | 1122 (65.38) | 0.53 (0.46- 0.61) | 8.48E-20 |
| *Gene body shore* | 34743 | 3359 (9.67) | 1.38 (1.33 - 1.43) | 1.31E-59 | 1430 (42.57) | 1929 (57.43) | 0.74 (0.67- 0.82) | 1.09E-09 |
| *Downstream region non-CGI* | 2581 | 319 (12.36) | 1.82 (1.61 - 2.05) | 1.89E-20 | 127 (39.81) | 192 (60.19) | 0.66 (0.48- 0.92) | 1.08E-02 |
| *Downstream region CGI* | 1947 | 98 (5.03) | 0.68 (0.55 - 0.84) | 1.49E-04 | 73 (74.49) | 25 (25.51) | 2.90 (1.53- 5.60) | 6.53E-04 |
| *Downstream region shelf* | 1257 | 104 (8.27) | 1.16 (0.94 - 1.42) | 1.40E-01 | 33 (31.73) | 71 (68.27) | 0.47 (0.25- 0.85) | 1.09E-02 |
| *Downstream region shore* | 1871 | 216 (11.54) | 1.68 (1.45 - 1.94) | 1.86E-11 | 130 (60.19) | 86 (39.81) | 1.51 (1.01- 2.25) | 4.21E-02 |

**SUPPLEMENTARY TABLE 7: The majority of ENCODE transcription factor binding-sites (TFBSs) are under-enriched for dDMPs.** TFBSs displaying a significant (p < 0.05) under or over-enrichment of significant dDMPs are shown, as well as direction of effect. Bonferroni correction (p < 1.25E-7) was used as a threshold to determine significance. (Slieker et al. 2013; The ENCODE Project Consortium 2012).

| **Transcription Factor Binding Site** | **Total probes** | **Significant**  **(p < 1.25E-7) probes (%)** | **Enrichment**  **(95% CI)** | **p value** | **Hypermethylated**  **(%)** | **Hypomethylated**  **(%)** | **Enrichment hypermethylated**  **(95% CI)** | **p value** |
| --- | --- | --- | --- | --- | --- | --- | --- | --- |
| **Under-enriched for dDMPs** | |  |  |  |  |  |  |  |
| *BRF1* | 235 | 0 (0.00) | 0.00 (0.00 - 0.20) | 3.94E-08 | 0 (NA) | 0 (NA) | 0.00 (0.00 - Inf) | 1.00E+00 |
| *GRP20* | 1380 | 0 (0.00) | 0.00 (0.00 - 0.03) | 3.53E-45 | 0 (NA) | 0 (NA) | 0.00 (0.00 - Inf) | 1.00E+00 |
| *POL3* | 162 | 0 (0.00) | 0.00 (0.00 - 0.30) | 1.04E-05 | 0 (NA) | 0 (NA) | 0.00 (0.00 - Inf) | 1.00E+00 |
| *SP2 (SC-643)* | 7492 | 7 (0.09) | 0.01 (0.00 - 0.02) | 2.56E-225 | 3 (42.86) | 4 (57.14) | 0.77 (0.05 - 10.67) | 1.00E+00 |
| *NELFE* | 1044 | 1 (0.10) | 0.01 (0.00 - 0.07) | 2.85E-32 | 0 (0.00) | 1 (100.00) | 0.00 (0.00 - Inf) | 1.00E+00 |
| *NFE2 (H-230)* | 1389 | 2 (0.14) | 0.02 (0.00 - 0.07) | 1.50E-41 | 1 (50.00) | 1 (50.00) | 1.00 (0.01 - 155.93) | 1.00E+00 |
| *SIX5* | 11436 | 17 (0.15) | 0.02 (0.01 - 0.03) | 0.00E+00 | 9 (52.94) | 8 (47.06) | 1.12 (0.23 - 5.42) | 1.00E+00 |
| *IRF3* | 5336 | 8 (0.15) | 0.02 (0.01 - 0.04) | 5.55E-156 | 2 (25.00) | 6 (75.00) | 0.36 (0.02 - 4.02) | 6.08E-01 |
| *THAP1 (SC-98174)* | 5016 | 16 (0.32) | 0.04 (0.02 - 0.07) | 7.16E-134 | 6 (37.50) | 10 (62.50) | 0.61 (0.12 - 3.02) | 7.22E-01 |
| *E2F1* | 9681 | 32 (0.33) | 0.04 (0.03 - 0.06) | 4.33E-254 | 15 (46.88) | 17 (53.13) | 0.88 (0.30 - 2.63) | 1.00E+00 |
| *NFYA* | 10704 | 39 (0.36) | 0.05 (0.03 - 0.06) | 1.35E-275 | 8 (20.51) | 31 (79.49) | 0.26 (0.08 - 0.78) | 8.83E-03 |
| *NRF1* | 15219 | 58 (0.38) | 0.05 (0.04 - 0.06) | 0.00E+00 | 35 (60.34) | 23 (39.66) | 1.52 (0.68 - 3.39) | 3.51E-01 |
| *E2F4* | 15708 | 63 (0.40) | 0.05 (0.04 - 0.07) | 0.00E+00 | 22 (34.92) | 41 (65.08) | 0.54 (0.25 - 1.17) | 1.05E-01 |
| *NFYB* | 11466 | 49 (0.43) | 0.06 (0.04 - 0.07) | 1.68E-285 | 15 (30.61) | 34 (69.39) | 0.45 (0.18 - 1.09) | 6.37E-02 |
| *GTF2B* | 4955 | 22 (0.44) | 0.06 (0.04 - 0.09) | 2.41E-124 | 10 (45.45) | 12 (54.55) | 0.84 (0.22 - 3.18) | 1.00E+00 |
| *KAT2A* | 175 | 1 (0.57) | 0.07 (0.00 - 0.42) | 5.32E-05 | 0 (0.00) | 1 (100.00) | 0.00 (0.00 - Inf) | 1.00E+00 |
| *SREBP1* | 4855 | 30 (0.62) | 0.08 (0.05 - 0.11) | 3.40E-112 | 19 (63.33) | 11 (36.67) | 1.71 (0.55 - 5.50) | 4.35E-01 |
| *IRF1* | 26886 | 172 (0.64) | 0.08 (0.07 - 0.10) | 0.00E+00 | 65 (37.79) | 107 (62.21) | 0.61 (0.39 - 0.95) | 2.96E-02 |
| *SREBP2* | 458 | 3 (0.66) | 0.09 (0.02 - 0.25) | 2.16E-11 | 1 (33.33) | 2 (66.67) | 0.58 (0.00 - 78.09) | 1.00E+00 |
| *CHD2 (N-1250)* | 19991 | 132 (0.66) | 0.09 (0.07 - 0.10) | 0.00E+00 | 66 (50.00) | 66 (50.00) | 1.00 (0.60 - 1.67) | 1.00E+00 |
| *TAF7 (SQ-8)* | 11442 | 78 (0.68) | 0.09 (0.07 - 0.11) | 5.88E-252 | 50 (64.10) | 28 (35.90) | 1.78 (0.90 - 3.57) | 1.05E-01 |
| *ZNF143 (16618-1-AP)* | 15394 | 112 (0.73) | 0.09 (0.08 - 0.11) | 0.00E+00 | 51 (45.54) | 61 (54.46) | 0.84 (0.48 - 1.46) | 5.93E-01 |
| *ELK4* | 14786 | 109 (0.74) | 0.10 (0.08 - 0.12) | 2.39E-315 | 33 (30.28) | 76 (69.72) | 0.44 (0.24 - 0.79) | 3.63E-03 |
| *ZBTB33* | 5164 | 39 (0.76) | 0.10 (0.07 - 0.13) | 9.26E-112 | 24 (61.54) | 15 (38.46) | 1.59 (0.59 - 4.37) | 3.63E-01 |
| *ETS1* | 17004 | 136 (0.80) | 0.10 (0.09 - 0.12) | 0.00E+00 | 76 (55.88) | 60 (44.12) | 1.27 (0.76 - 2.10) | 3.95E-01 |
| *WRNIP1* | 1723 | 14 (0.81) | 0.11 (0.06 - 0.18) | 2.20E-37 | 7 (50.00) | 7 (50.00) | 1.00 (0.18 - 5.58) | 1.00E+00 |
| *BRCA1 (C-1863)* | 8984 | 76 (0.85) | 0.11 (0.09 - 0.14) | 2.91E-184 | 17 (22.37) | 59 (77.63) | 0.29 (0.13 - 0.61) | 6.68E-04 |
| *BDP1* | 335 | 3 (0.90) | 0.12 (0.02 - 0.34) | 1.06E-07 | 2 (66.67) | 1 (33.33) | 1.73 (0.01 - 233.82) | 1.00E+00 |
| *HSF1* | 1067 | 10 (0.94) | 0.12 (0.06 - 0.23) | 2.24E-22 | 4 (40.00) | 6 (60.00) | 0.68 (0.08 - 5.36) | 1.00E+00 |
| *ATF3* | 7963 | 75 (0.94) | 0.12 (0.10 - 0.15) | 1.08E-156 | 32 (42.67) | 43 (57.33) | 0.75 (0.37 - 1.49) | 4.13E-01 |
| *POL2 (phosphoS2)* | 20923 | 209 (1.00) | 0.13 (0.11 - 0.15) | 0.00E+00 | 73 (34.93) | 136 (65.07) | 0.54 (0.36 - 0.81) | 2.13E-03 |
| *YY1* | 26132 | 269 (1.03) | 0.13 (0.12 - 0.15) | 0.00E+00 | 126 (46.84) | 143 (53.16) | 0.88 (0.62 - 1.25) | 4.90E-01 |
| *NR2C2* | 6750 | 70 (1.04) | 0.14 (0.11 - 0.17) | 9.71E-128 | 54 (77.14) | 16 (22.86) | 3.34 (1.54 - 7.52) | 1.45E-03 |
| *BCLAF1 (M33-P5B11)* | 7675 | 80 (1.04) | 0.14 (0.11 - 0.17) | 2.08E-144 | 35 (43.75) | 45 (56.25) | 0.78 (0.40 - 1.52) | 5.26E-01 |
| *HMGN3* | 23082 | 247 (1.07) | 0.14 (0.12 - 0.16) | 0.00E+00 | 111 (44.94) | 136 (55.06) | 0.82 (0.56 - 1.18) | 2.80E-01 |
| *ZZZ3* | 642 | 7 (1.09) | 0.14 (0.06 - 0.29) | 6.10E-13 | 2 (28.57) | 5 (71.43) | 0.43 (0.02 - 6.32) | 5.92E-01 |
| *SIN3A (k-20)* | 32761 | 387 (1.18) | 0.15 (0.14 - 0.17) | 0.00E+00 | 198 (51.16) | 189 (48.84) | 1.05 (0.78 - 1.40) | 7.74E-01 |
| *CCNT2* | 27001 | 336 (1.24) | 0.16 (0.15 - 0.18) | 0.00E+00 | 161 (47.92) | 175 (52.08) | 0.92 (0.67 - 1.26) | 6.43E-01 |
| *PBX3* | 6742 | 84 (1.25) | 0.16 (0.13 - 0.20) | 5.24E-116 | 46 (54.76) | 38 (45.24) | 1.21 (0.63 - 2.32) | 6.43E-01 |
| *HA-E2F1* | 49025 | 618 (1.26) | 0.16 (0.15 - 0.18) | 0.00E+00 | 224 (36.25) | 394 (63.75) | 0.57 (0.45 - 0.72) | 1.35E-06 |
| *GABP* | 28921 | 370 (1.28) | 0.17 (0.15 - 0.19) | 0.00E+00 | 205 (55.41) | 165 (44.59) | 1.24 (0.92 - 1.68) | 1.62E-01 |
| *HEY1* | 40796 | 533 (1.31) | 0.17 (0.16 - 0.19) | 0.00E+00 | 272 (51.03) | 261 (48.97) | 1.04 (0.81 - 1.33) | 7.59E-01 |
| *ZEB1 (SC-25388)* | 8582 | 118 (1.37) | 0.18 (0.15 - 0.22) | 1.66E-138 | 75 (63.56) | 43 (36.44) | 1.74 (1.00 - 3.04) | 4.85E-02 |
| *PPARGC1A* | 421 | 6 (1.43) | 0.19 (0.07 - 0.41) | 7.71E-08 | 3 (50.00) | 3 (50.00) | 1.00 (0.06 - 15.64) | 1.00E+00 |
| *SETDB1* | 5901 | 86 (1.46) | 0.19 (0.15 - 0.24) | 1.32E-92 | 40 (46.51) | 46 (53.49) | 0.87 (0.46 - 1.65) | 7.60E-01 |
| *PAX5 (C20)* | 20103 | 296 (1.47) | 0.19 (0.17 - 0.22) | 3.97E-302 | 140 (47.30) | 156 (52.70) | 0.90 (0.64 - 1.26) | 5.65E-01 |
| *TAF1* | 57616 | 866 (1.50) | 0.20 (0.18 - 0.21) | 0.00E+00 | 503 (58.08) | 363 (41.92) | 1.39 (1.14 - 1.68) | 8.73E-04 |
| *E2F6* | 22689 | 346 (1.52) | 0.20 (0.18 - 0.22) | 0.00E+00 | 167 (48.27) | 179 (51.73) | 0.93 (0.68 - 1.27) | 7.04E-01 |
| *SRF* | 10471 | 160 (1.53) | 0.20 (0.17 - 0.23) | 1.44E-156 | 80 (50.00) | 80 (50.00) | 1.00 (0.63 - 1.59) | 1.00E+00 |
| *MXI1 (bHLH)* | 19193 | 299 (1.56) | 0.20 (0.18 - 0.23) | 9.65E-278 | 89 (29.77) | 210 (70.23) | 0.42 (0.30 - 0.60) | 4.93E-07 |
| *CTCFL (SC-98982)* | 7537 | 119 (1.58) | 0.21 (0.17 - 0.25) | 4.40E-111 | 59 (49.58) | 60 (50.42) | 0.98 (0.57 - 1.69) | 1.00E+00 |
| *YY1 (C-20)* | 33867 | 540 (1.59) | 0.21 (0.19 - 0.23) | 0.00E+00 | 272 (50.37) | 268 (49.63) | 1.01 (0.79 - 1.30) | 9.51E-01 |
| *ZBTB7A (SC-34508)* | 20364 | 343 (1.68) | 0.22 (0.20 - 0.25) | 4.48E-277 | 188 (54.81) | 155 (45.19) | 1.21 (0.89 - 1.66) | 2.21E-01 |
| *E2F6 (H-50)* | 28946 | 489 (1.69) | 0.22 (0.20 - 0.24) | 0.00E+00 | 267 (54.60) | 222 (45.40) | 1.20 (0.93 - 1.56) | 1.59E-01 |
| *OCT2* | 16306 | 289 (1.77) | 0.23 (0.21 - 0.26) | 1.02E-214 | 130 (44.98) | 159 (55.02) | 0.82 (0.58 - 1.15) | 2.44E-01 |
| *POU2F2* | 16217 | 289 (1.78) | 0.23 (0.21 - 0.26) | 1.61E-212 | 130 (44.98) | 159 (55.02) | 0.82 (0.58 - 1.15) | 2.44E-01 |
| *ELF1 (SC-631)* | 43708 | 783 (1.79) | 0.24 (0.22 - 0.25) | 0.00E+00 | 404 (51.60) | 379 (48.40) | 1.07 (0.87 - 1.31) | 5.44E-01 |
| *GTF2F1 (RAP-74)* | 16373 | 296 (1.81) | 0.24 (0.21 - 0.27) | 5.20E-212 | 102 (34.46) | 194 (65.54) | 0.53 (0.37 - 0.74) | 1.75E-04 |
| *EGR1* | 29022 | 537 (1.85) | 0.24 (0.22 - 0.27) | 0.00E+00 | 274 (51.02) | 263 (48.98) | 1.04 (0.81 - 1.33) | 7.60E-01 |
| *USF1 (SC-8983)* | 16146 | 299 (1.85) | 0.24 (0.22 - 0.27) | 6.98E-205 | 103 (34.45) | 196 (65.55) | 0.53 (0.37 - 0.74) | 1.36E-04 |
| *RFX5 (N-494)* | 17473 | 330 (1.89) | 0.25 (0.22 - 0.28) | 2.80E-217 | 123 (37.27) | 207 (62.73) | 0.59 (0.43 - 0.82) | 1.27E-03 |
| *NFKB* | 33129 | 667 (2.01) | 0.27 (0.25 - 0.29) | 0.00E+00 | 298 (44.68) | 369 (55.32) | 0.81 (0.65 - 1.01) | 5.49E-02 |
| *TBP* | 52581 | 1059 (2.01) | 0.27 (0.25 - 0.28) | 0.00E+00 | 507 (47.88) | 552 (52.12) | 0.92 (0.77 - 1.09) | 3.39E-01 |
| *POLR3A* | 764 | 16 (2.09) | 0.28 (0.16 - 0.45) | 4.12E-10 | 9 (56.25) | 7 (43.75) | 1.28 (0.26 - 6.42) | 1.00E+00 |
| *POLR2A (Pol2(b))* | 21800 | 468 (2.15) | 0.28 (0.26 - 0.31) | 3.20E-237 | 175 (37.39) | 293 (62.61) | 0.60 (0.46 - 0.78) | 1.30E-04 |
| *GTF3C2* | 1069 | 24 (2.25) | 0.30 (0.19 - 0.44) | 8.91E-13 | 7 (29.17) | 17 (70.83) | 0.42 (0.10 - 1.57) | 2.38E-01 |
| *SP1* | 32631 | 743 (2.28) | 0.30 (0.28 - 0.32) | 0.00E+00 | 436 (58.68) | 307 (41.32) | 1.42 (1.15 - 1.75) | 8.54E-04 |
| *SUPT20H* | 130 | 3 (2.31) | 0.30 (0.06 - 0.91) | 2.61E-02 | 1 (33.33) | 2 (66.67) | 0.58 (0.00 - 78.09) | 1.00E+00 |
| *REST* | 17427 | 418 (2.40) | 0.32 (0.29 - 0.35) | 4.34E-169 | 150 (35.89) | 268 (64.11) | 0.56 (0.42 - 0.75) | 4.92E-05 |
| *POLR2A (Pol2-4H8)* | 60326 | 1451 (2.41) | 0.32 (0.30 - 0.34) | 0.00E+00 | 638 (43.97) | 813 (56.03) | 0.78 (0.68 - 0.91) | 1.21E-03 |
| *MYC* | 34021 | 835 (2.45) | 0.32 (0.30 - 0.35) | 6.96E-310 | 322 (38.56) | 513 (61.44) | 0.63 (0.51 - 0.77) | 2.80E-06 |
| *SMARCB1* | 10218 | 252 (2.47) | 0.33 (0.29 - 0.37) | 8.91E-98 | 90 (35.71) | 162 (64.29) | 0.56 (0.38 - 0.81) | 1.60E-03 |
| *CTCF (C-20)* | 12565 | 320 (2.55) | 0.34 (0.30 - 0.38) | 1.25E-114 | 178 (55.63) | 142 (44.38) | 1.25 (0.91 - 1.73) | 1.78E-01 |
| *PAX5 (PAX5-N19)* | 9167 | 250 (2.73) | 0.36 (0.32 - 0.41) | 3.48E-77 | 118 (47.20) | 132 (52.80) | 0.89 (0.62 - 1.29) | 5.91E-01 |
| *POL2* | 101434 | 2945 (2.90) | 0.39 (0.37 - 0.40) | 0.00E+00 | 1268 (43.06) | 1677 (56.94) | 0.76 (0.68 - 0.84) | 9.76E-08 |
| *USF2* | 12881 | 379 (2.94) | 0.39 (0.35 - 0.43) | 1.83E-95 | 175 (46.17) | 204 (53.83) | 0.86 (0.64 - 1.15) | 3.09E-01 |
| *MAX* | 29460 | 900 (3.05) | 0.41 (0.38 - 0.44) | 8.21E-197 | 401 (44.56) | 499 (55.44) | 0.80 (0.66 - 0.97) | 2.34E-02 |
| *MEF2A* | 6223 | 191 (3.07) | 0.41 (0.35 - 0.47) | 1.88E-44 | 72 (37.70) | 119 (62.30) | 0.61 (0.39 - 0.93) | 1.76E-02 |
| *BHLHE40* | 2801 | 89 (3.18) | 0.42 (0.34 - 0.52) | 8.42E-20 | 52 (58.43) | 37 (41.57) | 1.40 (0.74 - 2.66) | 2.93E-01 |
| *ZNF263* | 26785 | 856 (3.20) | 0.43 (0.40 - 0.46) | 2.90E-166 | 334 (39.02) | 522 (60.98) | 0.64 (0.53 - 0.78) | 5.98E-06 |
| *SMARCC2* | 2487 | 80 (3.22) | 0.43 (0.34 - 0.54) | 2.24E-17 | 35 (43.75) | 45 (56.25) | 0.78 (0.40 - 1.52) | 5.26E-01 |
| *SUZ12* | 7036 | 229 (3.25) | 0.43 (0.38 - 0.50) | 4.94E-45 | 184 (80.35) | 45 (19.65) | 4.08 (2.64 - 6.36) | 7.65E-12 |
| *NANOG (SC-33759)* | 6153 | 204 (3.32) | 0.44 (0.38 - 0.51) | 2.59E-38 | 163 (79.90) | 41 (20.10) | 3.96 (2.51 - 6.33) | 3.04E-10 |
| *GATA1* | 14886 | 503 (3.38) | 0.45 (0.41 - 0.49) | 9.08E-86 | 168 (33.40) | 335 (66.60) | 0.50 (0.39 - 0.65) | 1.04E-07 |
| *TCF12* | 12218 | 416 (3.40) | 0.45 (0.41 - 0.50) | 4.39E-70 | 263 (63.22) | 153 (36.78) | 1.72 (1.29 - 2.29) | 1.55E-04 |
| *CTCF (SC-5916)* | 14196 | 492 (3.47) | 0.46 (0.42 - 0.51) | 6.37E-78 | 267 (54.27) | 225 (45.73) | 1.19 (0.92 - 1.54) | 2.02E-01 |
| *CTBP2* | 4000 | 142 (3.55) | 0.48 (0.40 - 0.56) | 2.11E-22 | 104 (73.24) | 38 (26.76) | 2.73 (1.62 - 4.64) | 8.67E-05 |
| *BCL3* | 3622 | 130 (3.59) | 0.48 (0.40 - 0.57) | 5.75E-20 | 59 (45.38) | 71 (54.62) | 0.83 (0.50 - 1.39) | 5.35E-01 |
| *USF1* | 23835 | 871 (3.65) | 0.49 (0.46 - 0.52) | 2.23E-113 | 469 (53.85) | 402 (46.15) | 1.17 (0.96 - 1.41) | 1.14E-01 |
| *NFE2* | 944 | 35 (3.71) | 0.50 (0.34 - 0.70) | 8.98E-06 | 16 (45.71) | 19 (54.29) | 0.84 (0.29 - 2.40) | 8.11E-01 |
| *SMC3 (ab9263)* | 14945 | 556 (3.72) | 0.50 (0.46 - 0.54) | 4.21E-70 | 252 (45.32) | 304 (54.68) | 0.83 (0.65 - 1.06) | 1.33E-01 |
| *IRF4 (M-17)* | 5873 | 224 (3.81) | 0.51 (0.45 - 0.59) | 2.64E-27 | 99 (44.20) | 125 (55.80) | 0.79 (0.54 - 1.17) | 2.56E-01 |
| *JUND (eGFP-JunD)* | 11423 | 447 (3.91) | 0.53 (0.48 - 0.58) | 5.60E-48 | 206 (46.09) | 241 (53.91) | 0.85 (0.65 - 1.12) | 2.55E-01 |
| *TFAP2C* | 17213 | 694 (4.03) | 0.54 (0.50 - 0.59) | 2.83E-65 | 244 (35.16) | 450 (64.84) | 0.54 (0.43 - 0.68) | 2.89E-08 |
| *MEF2C (SC-13268)* | 2585 | 105 (4.06) | 0.55 (0.44 - 0.66) | 4.01E-11 | 39 (37.14) | 66 (62.86) | 0.59 (0.33 - 1.06) | 7.03E-02 |
| *TFAP2A* | 11548 | 473 (4.10) | 0.55 (0.50 - 0.60) | 5.60E-43 | 183 (38.69) | 290 (61.31) | 0.63 (0.48 - 0.82) | 5.17E-04 |
| *STAT1* | 9459 | 388 (4.10) | 0.55 (0.50 - 0.61) | 1.79E-35 | 116 (29.90) | 272 (70.10) | 0.43 (0.31 - 0.58) | 1.46E-08 |
| *TCF4* | 17396 | 720 (4.14) | 0.56 (0.52 - 0.60) | 3.02E-61 | 353 (49.03) | 367 (50.97) | 0.96 (0.78 - 1.19) | 7.52E-01 |
| *HDAC2 (SC-6296)* | 11133 | 478 (4.29) | 0.58 (0.53 - 0.64) | 3.99E-36 | 268 (56.07) | 210 (43.93) | 1.28 (0.98 - 1.66) | 6.95E-02 |
| *SIRT6* | 867 | 38 (4.38) | 0.59 (0.42 - 0.82) | 9.52E-04 | 18 (47.37) | 20 (52.63) | 0.90 (0.33 - 2.43) | 1.00E+00 |
| *STAT2* | 2394 | 106 (4.43) | 0.60 (0.49 - 0.73) | 3.06E-08 | 37 (34.91) | 69 (65.09) | 0.54 (0.30 - 0.97) | 3.69E-02 |
| *SPI1* | 13249 | 587 (4.43) | 0.60 (0.55 - 0.65) | 1.86E-38 | 263 (44.80) | 324 (55.20) | 0.81 (0.64 - 1.03) | 7.95E-02 |
| *TRIM28* | 13026 | 580 (4.45) | 0.60 (0.55 - 0.65) | 3.43E-37 | 274 (47.24) | 306 (52.76) | 0.90 (0.71 - 1.13) | 3.78E-01 |
| *CTCF* | 44931 | 2004 (4.46) | 0.60 (0.57 - 0.63) | 8.17E-116 | 949 (47.36) | 1055 (52.64) | 0.90 (0.79 - 1.02) | 1.00E-01 |
| *FOSL2* | 8631 | 397 (4.60) | 0.62 (0.56 - 0.69) | 1.01E-22 | 203 (51.13) | 194 (48.87) | 1.05 (0.78 - 1.40) | 7.76E-01 |
| *RXRA* | 7463 | 365 (4.89) | 0.66 (0.60 - 0.74) | 8.80E-16 | 228 (62.47) | 137 (37.53) | 1.66 (1.23 - 2.26) | 7.77E-04 |
| *EBF1 (C-8)* | 10518 | 528 (5.02) | 0.68 (0.62 - 0.75) | 3.91E-19 | 237 (44.89) | 291 (55.11) | 0.81 (0.63 - 1.04) | 1.09E-01 |
| *JUNB (eGFP-JunB)* | 4310 | 221 (5.13) | 0.70 (0.61 - 0.80) | 5.30E-08 | 115 (52.04) | 106 (47.96) | 1.08 (0.73 - 1.60) | 7.04E-01 |
| *RAD21* | 19809 | 1033 (5.21) | 0.71 (0.67 - 0.76) | 3.19E-28 | 486 (47.05) | 547 (52.95) | 0.89 (0.74 - 1.06) | 1.87E-01 |
| *FOS* | 16423 | 864 (5.26) | 0.72 (0.67 - 0.77) | 9.48E-23 | 317 (36.69) | 547 (63.31) | 0.58 (0.48 - 0.71) | 2.98E-08 |
| *JUND* | 14247 | 761 (5.34) | 0.73 (0.68 - 0.78) | 1.94E-18 | 322 (42.31) | 439 (57.69) | 0.73 (0.60 - 0.90) | 2.85E-03 |
| *CEBPB* | 17526 | 959 (5.47) | 0.75 (0.70 - 0.80) | 2.97E-19 | 336 (35.04) | 623 (64.96) | 0.54 (0.45 - 0.65) | 3.65E-11 |
| *HNF4A* | 3376 | 192 (5.69) | 0.78 (0.67 - 0.90) | 5.59E-04 | 104 (54.17) | 88 (45.83) | 1.18 (0.78 - 1.80) | 4.75E-01 |
| *SMARCC1* | 4505 | 265 (5.88) | 0.81 (0.71 - 0.91) | 5.49E-04 | 106 (40.00) | 159 (60.00) | 0.67 (0.47 - 0.95) | 2.31E-02 |
| *HNF4G (SC-6558)* | 5217 | 307 (5.88) | 0.81 (0.72 - 0.91) | 2.14E-04 | 180 (58.63) | 127 (41.37) | 1.42 (1.02 - 1.98) | 3.51E-02 |
| *MAFK (SC-477)* | 2883 | 179 (6.21) | 0.85 (0.73 - 0.99) | 4.26E-02 | 85 (47.49) | 94 (52.51) | 0.90 (0.58 - 1.40) | 6.72E-01 |
| *GATA3 (SC-268)* | 5898 | 371 (6.29) | 0.87 (0.78 - 0.96) | 7.61E-03 | 120 (32.35) | 251 (67.65) | 0.48 (0.35 - 0.65) | 1.17E-06 |
|  |  |  |  |  |  |  |  |  |
| **Enriched for dDMPs** |  |  |  |  |  |  |  |  |
| *JUN* | 9476 | 782 (8.25) | 1.16 (1.08 - 1.25) | 1.05E-04 | 287 (36.70) | 495 (63.30) | 0.58 (0.47 - 0.71) | 1.41E-07 |
| *FOXA2 (SC-6554)* | 5199 | 443 (8.52) | 1.20 (1.09 - 1.33) | 3.28E-04 | 233 (52.60) | 210 (47.40) | 1.11 (0.84 - 1.46) | 4.60E-01 |
| *BATF* | 2897 | 251 (8.66) | 1.22 (1.07 - 1.39) | 2.75E-03 | 101 (40.24) | 150 (59.76) | 0.67 (0.47 - 0.97) | 3.13E-02 |
| *GATA2 (eGFP-GATA2)* | 2150 | 189 (8.79) | 1.24 (1.07 - 1.45) | 5.05E-03 | 82 (43.39) | 107 (56.61) | 0.77 (0.50 - 1.17) | 2.16E-01 |
| *NR3C1* | 8402 | 752 (8.95) | 1.27 (1.17 - 1.37) | 2.42E-09 | 278 (36.97) | 474 (63.03) | 0.59 (0.47 - 0.72) | 4.35E-07 |
| *BCL11A* | 2383 | 217 (9.11) | 1.29 (1.12 - 1.49) | 4.65E-04 | 93 (42.86) | 124 (57.14) | 0.75 (0.50 - 1.11) | 1.49E-01 |
| *ESR1 (ERalpha_a)* | 5695 | 533 (9.36) | 1.33 (1.22 - 1.46) | 1.53E-09 | 231 (43.34) | 302 (56.66) | 0.77 (0.60 - 0.98) | 3.16E-02 |
| *POU5F1 (SC-9081)* | 502 | 48 (9.56) | 1.36 (0.99 - 1.84) | 4.63E-02 | 38 (79.17) | 10 (20.83) | 3.74 (1.43 - 10.44) | 5.15E-03 |
| *FOS (eGFP-FOS)* | 2196 | 223 (10.15) | 1.46 (1.26 - 1.68) | 4.12E-07 | 99 (44.39) | 124 (55.61) | 0.80 (0.54 - 1.18) | 2.55E-01 |
| *GATA2* | 10882 | 1132 (10.40) | 1.50 (1.41 - 1.60) | 2.52E-33 | 487 (43.02) | 645 (56.98) | 0.76 (0.64 - 0.89) | 1.01E-03 |
| *GATA2 (CG2-96)* | 1551 | 166 (10.70) | 1.55 (1.31 - 1.82) | 4.75E-07 | 88 (53.01) | 78 (46.99) | 1.13 (0.72 - 1.78) | 6.61E-01 |
| *P300 (N-15)* | 3186 | 369 (11.58) | 1.69 (1.51 - 1.89) | 9.09E-19 | 113 (30.62) | 256 (69.38) | 0.44 (0.32 - 0.60) | 8.99E-08 |

**SUPPLEMENTARY TABLE 8: dDMPs in genomic regions characterized by alternative transcription events** (Slieker et al. 2013, Wang et al. 2008; Koscielny et al. 2009; Flicek et al. 2014). Bonferroni correction (p < 1.25E-7) was used as a threshold to determine significance.

| **Alternative transcription event** | **Total probes** | **Significant**  **(p < 1.25E-7) probes (%)** | **Enrichment**  **(95% CI)** | **p value** | **Hypermethylated**  **(%)** | **Hypomethylated**  **(%)** | **Enrichment hypermethylated**  **(95% CI)** | **p value** |
| --- | --- | --- | --- | --- | --- | --- | --- | --- |
| *Alternative 3’ splice site* | 3545 | 144 (4.06) | 0.55 (0.46 - 0.65) | 1.16E-14 | 43 (29.86) | 101 (70.14) | 0.43 (0.25 - 0.71) | 7.23E-04 |
| *Alternative 5’ splice site* | 3584 | 180 (5.02) | 0.68 (0.58 - 0.79) | 1.86E-07 | 66 (36.67) | 114 (63.33) | 0.58 (0.37 - 0.90) | 1.43E-02 |
| *Alternative first exon* | 61181 | 3578 (5.85) | 0.80 (0.77 - 0.83) | 3.11E-35 | 1578 (44.10) | 2000 (55.90) | 0.79 (0.72 - 0.87) | 6.53E-07 |
| *Alternative last exon* | 10027 | 985 (9.82) | 1.41 (1.31 - 1.50) | 1.05E-21 | 424 (43.05) | 561 (56.95) | 0.76 (0.63 - 0.91) | 2.12E-03 |
| *Cassette exon* | 64806 | 5340 (8.24) | 1.16 (1.12 - 1.19) | 8.09E-21 | 2227 (41.70) | 3113 (58.30) | 0.72 (0.66 - 0.77) | 8.81E-18 |
| *Constitutive exon* | 21982 | 1009 (4.59) | 0.62 (0.58 - 0.66) | 2.11E-54 | 444 (44.00) | 565 (56.00) | 0.79 (0.66 - 0.94) | 7.43E-03 |
| *Exon isoforms* | 179 | 5 (2.79) | 0.37 (0.12 - 0.88) | 1.95E-02 | 3 (60.00) | 2 (40.00) | 1.43 (0.05 - 39.79) | 1.00E+00 |
| *Intron isoforms* | 29601 | 1885 (6.37) | 0.88 (0.84 - 0.92) | 7.77E-08 | 701 (37.19) | 1184 (62.81) | 0.59 (0.52 - 0.68) | 2.25E-15 |
| *Intron retention* | 17240 | 813 (4.72) | 0.64 (0.59 - 0.69) | 3.41E-39 | 307 (37.76) | 506 (62.24) | 0.61 (0.50 - 0.74) | 7.29E-07 |
| *Mutually exclusive exon* | 14212 | 1152 (8.11) | 1.14 (1.07 - 1.21) | 4.62E-05 | 480 (41.67) | 672 (58.33) | 0.71 (0.60 - 0.84) | 7.06E-05 |

**SUPPLEMENTARY TABLE 9 – Sex differences in autosomal DNA methylation identified in both adult and fetal cortex samples.**

|  |  |  | **Adult cortex (Xu *et al.*, 2014)** | | | | **Fetal cortex** | |
| --- | --- | --- | --- | --- | --- | --- | --- | --- |
| **Probe** | **Gene** | **Chr** | **Male (β)** | **Female (β)** | **β difference (Female-Male)** | **p value** | **β difference (Female-Male)** | **p value** |
| cg04946709 |  | 16 | 0.79 | 0.71 | -0.08 | 1.84E-21 | -0.08 | 1.05E-48 |
| cg11643285 | *RFTN1* | 3 | 0.73 | 0.85 | 0.12 | 1.62E-20 | 0.11 | 8.78E-65 |
| cg03691818 | *KRT77* | 12 | 0.05 | 0.11 | 0.06 | 1.05E-19 | 0.07 | 7.59E-69 |
| cg15817705 |  | 1 | 0.79 | 0.69 | -0.10 | 9.89E-19 | -0.09 | 5.81E-47 |
| cg17232883 |  | 11 | 0.09 | 0.14 | 0.05 | 9.49E-17 | 0.04 | 1.40E-17 |
| cg22227586 | *FAM35A; GLUD1* | 10 | 0.03 | 0.05 | 0.02 | 1.39E-14 | 0.01 | 7.71E-18 |
| cg26516287 | *SCIN* | 7 | 0.77 | 0.81 | 0.04 | 8.94E-14 | 0.04 | 3.76E-16 |
| cg01225095 | *YARS2* | 12 | 0.11 | 0.15 | 0.04 | 1.92E-13 | 0.04 | 1.48E-11 |
| cg06710937 |  | 13 | 0.06 | 0.10 | 0.04 | 1.11E-12 | 0.04 | 6.65E-38 |
| cg16169375 | *DNPH1* | 6 | 0.08 | 0.11 | 0.03 | 2.04E-12 | 0.02 | 1.13E-11 |
| cg03608000 | *ZNF69* | 19 | 0.06 | 0.08 | 0.02 | 3.27E-12 | 0.01 | 8.53E-10 |
| cg24016844 | *LRIF1* | 1 | 0.13 | 0.10 | -0.03 | 6.87E-12 | -0.04 | 5.25E-31 |
| cg06962442 |  | 16 | 0.09 | 0.07 | -0.02 | 3.45E-11 | -0.03 | 2.40E-20 |
| cg10047026 | *ARRB2* | 17 | 0.12 | 0.16 | 0.04 | 1.01E-10 | 0.03 | 1.86E-11 |
| cg12611723 | *NPDC1* | 9 | 0.09 | 0.11 | 0.02 | 2.74E-10 | 0.01 | 7.30E-09 |
| cg07816873 | *ERC1* | 12 | 0.21 | 0.26 | 0.05 | 4.54E-10 | 0.03 | 2.95E-12 |
| cg21148594 |  | 14 | 0.69 | 0.61 | -0.08 | 8.19E-10 | -0.08 | 3.50E-29 |
| cg17612569 | *GABPA; ATP5J* | 21 | 0.09 | 0.06 | -0.03 | 1.05E-09 | -0.03 | 2.81E-26 |
| cg17743279 | *CDK6* | 7 | 0.04 | 0.05 | 0.01 | 1.34E-09 | 0.01 | 4.11E-15 |
| cg13323902 | *VTRNA1-1* | 5 | 0.17 | 0.22 | 0.05 | 1.77E-09 | 0.05 | 8.66E-17 |
| cg12900929 | *PRDM4* | 12 | 0.10 | 0.13 | 0.03 | 2.24E-09 | 0.02 | 5.93E-10 |
| cg06644124 | *ZNF281* | 1 | 0.23 | 0.29 | 0.06 | 2.66E-09 | 0.03 | 4.27E-09 |
| cg03218192 | *AP2B1* | 17 | 0.26 | 0.34 | 0.08 | 4.15E-09 | 0.05 | 1.23E-33 |
| cg22345911 | *CSNK1D* | 17 | 0.06 | 0.08 | 0.02 | 6.05E-09 | 0.02 | 4.08E-45 |
| cg09971754 | *ANKRD11* | 16 | 0.07 | 0.05 | -0.02 | 7.89E-09 | -0.03 | 4.15E-28 |
| cg23814743 | *NICN1* | 3 | 0.27 | 0.32 | 0.04 | 1.05E-08 | 0.04 | 1.47E-16 |
| cg06642617 | *CCT7; PRADC1* | 2 | 0.06 | 0.03 | -0.03 | 1.13E-08 | -0.02 | 5.05E-33 |
| cg22889142 | *A1BG-AS1; A1BG* | 19 | 0.51 | 0.57 | 0.06 | 1.13E-08 | 0.06 | 1.03E-15 |
| cg05100634 | *SMAD2* | 18 | 0.04 | 0.05 | 0.01 | 1.41E-08 | 0.01 | 8.32E-17 |
| cg17561891 | *TMEM243* | 7 | 0.12 | 0.14 | 0.03 | 2.30E-08 | 0.03 | 1.41E-20 |
| cg25438440 | *CLDND1* | 3 | 0.08 | 0.09 | 0.01 | 4.25E-08 | 0.02 | 1.95E-13 |
| cg12611527 |  | 2 | 0.08 | 0.10 | 0.02 | 4.53E-08 | 0.02 | 6.54E-12 |
| cg09725915 |  | 2 | 0.04 | 0.05 | 0.01 | 4.57E-08 | 0.01 | 3.80E-12 |
| cg03253995 | *EIF4A1; SNORA48* | 17 | 0.39 | 0.46 | 0.07 | 6.11E-08 | 0.06 | 5.76E-19 |
| cg26213873 | *CTTNBP2NL* | 1 | 0.10 | 0.12 | 0.02 | 8.30E-08 | 0.01 | 3.12E-09 |
| cg16810031 | *ZPBP2* | 17 | 0.71 | 0.80 | 0.09 | 1.12E-07 | 0.11 | 6.93E-28 |
| cg09513416 |  | 6 | 0.78 | 0.72 | -0.06 | 1.91E-07 | -0.07 | 1.87E-17 |
| cg03687700 | *FKBP1B* | 2 | 0.19 | 0.27 | 0.08 | 2.36E-07 | 0.12 | 9.76E-53 |
| cg23719534 |  | 15 | 0.85 | 0.89 | 0.04 | 5.47E-07 | 0.08 | 2.17E-43 |
| cg14030268 | *PDZD8* | 10 | 0.04 | 0.05 | 0.01 | 5.80E-07 | 0.01 | 1.36E-13 |
| cg26095395 |  | 4 | 0.60 | 0.55 | -0.05 | 7.64E-07 | -0.06 | 5.28E-12 |
| cg11065518 | *MDH1B; FASTKD2* | 2 | 0.08 | 0.09 | 0.01 | 1.21E-06 | 0.02 | 1.08E-11 |
| cg06877024 |  | 6 | 0.23 | 0.20 | -0.02 | 1.47E-06 | -0.03 | 1.52E-14 |
| cg08721112 |  | 1 | 0.55 | 0.50 | -0.05 | 1.47E-06 | -0.03 | 8.90E-08 |
| cg12900170 | *COX7C* | 5 | 0.12 | 0.15 | 0.03 | 1.52E-06 | 0.03 | 3.57E-08 |
| cg11841231 | *PARD3B* | 2 | 0.82 | 0.78 | -0.04 | 2.05E-06 | -0.10 | 2.20E-38 |
| cg12266551 | *TOMM40* | 19 | 0.05 | 0.06 | 0.01 | 2.19E-06 | 0.01 | 2.10E-15 |
| cg12691488 |  | 1 | 0.39 | 0.32 | -0.07 | 2.42E-06 | -0.21 | 1.66E-80 |
| cg11240062 |  | 8 | 0.48 | 0.35 | -0.13 | 3.30E-06 | -0.13 | 1.87E-18 |
| cg11460509 |  | 21 | 0.74 | 0.76 | 0.03 | 3.30E-06 | 0.03 | 8.14E-12 |
| cg02625623 |  | 13 | 0.84 | 0.80 | -0.04 | 3.32E-06 | -0.03 | 3.24E-15 |
| cg26533311 |  | 6 | 0.38 | 0.45 | 0.08 | 3.41E-06 | 0.06 | 1.05E-08 |
| cg03151810 |  | 8 | 0.73 | 0.63 | -0.11 | 4.50E-06 | -0.13 | 1.15E-15 |
| cg27462519 |  | 16 | 0.69 | 0.60 | -0.10 | 5.28E-06 | -0.11 | 1.58E-17 |
| cg12204423 | *JADE1* | 4 | 0.16 | 0.14 | -0.02 | 6.62E-06 | -0.03 | 1.25E-25 |
| cg10749792 | *PSPH; CCT6A* | 7 | 0.04 | 0.05 | 0.01 | 6.70E-06 | 0.01 | 7.15E-12 |
| cg19712189 | *TDRD12* | 19 | 0.87 | 0.82 | -0.06 | 7.23E-06 | -0.06 | 5.27E-08 |
| cg12736438 | *RAB20* | 13 | 0.12 | 0.14 | 0.02 | 7.73E-06 | 0.02 | 2.79E-10 |
| cg01065780 | *MB* | 22 | 0.10 | 0.11 | 0.01 | 7.80E-06 | 0.02 | 1.81E-11 |
| cg03894796 |  | 8 | 0.47 | 0.38 | -0.09 | 8.99E-06 | -0.09 | 1.91E-20 |
| cg08906898 | *RBM39* | 20 | 0.80 | 0.76 | -0.04 | 1.04E-05 | -0.04 | 7.20E-26 |
| cg16844333 |  | 10 | 0.45 | 0.52 | 0.07 | 1.76E-05 | 0.06 | 1.90E-08 |
| cg18407136 | *C15orf40* | 15 | 0.73 | 0.76 | 0.04 | 1.96E-05 | 0.04 | 1.79E-12 |
| cg22429776 | *ST7L; CAPZA1* | 1 | 0.04 | 0.04 | 0.01 | 2.06E-05 | 0.01 | 4.94E-14 |
| cg09639931 | *ZPBP2* | 17 | 0.23 | 0.29 | 0.06 | 2.20E-05 | 0.05 | 6.54E-12 |
| cg26612727 | *ZPBP2* | 17 | 0.34 | 0.39 | 0.05 | 2.29E-05 | 0.06 | 9.76E-13 |
| cg20050113 | *SLC9A2* | 2 | 0.38 | 0.43 | 0.05 | 2.57E-05 | 0.07 | 4.75E-16 |
| cg10334916 | *RNPEPL1* | 2 | 0.12 | 0.10 | -0.01 | 2.62E-05 | -0.02 | 2.59E-17 |
| cg09045105 | *BOLA1* | 1 | 0.62 | 0.68 | 0.05 | 3.09E-05 | 0.06 | 9.61E-15 |
| cg17622922 | *MCAM* | 11 | 0.16 | 0.17 | 0.01 | 3.55E-05 | -0.01 | 1.30E-09 |
| cg14870223 | *WDR48* | 3 | 0.52 | 0.56 | 0.04 | 3.60E-05 | 0.05 | 3.91E-08 |
| cg26952796 |  | 19 | 0.22 | 0.17 | -0.05 | 3.71E-05 | -0.05 | 3.44E-13 |
| cg11388673 |  | 8 | 0.80 | 0.72 | -0.09 | 4.28E-05 | -0.10 | 1.75E-11 |
| cg14266237 | *KLF1* | 19 | 0.48 | 0.58 | 0.09 | 4.30E-05 | 0.07 | 7.98E-10 |
| cg03934782 | *VAT1L* | 16 | 0.30 | 0.34 | 0.04 | 4.64E-05 | 0.02 | 7.05E-08 |
| cg18417954 | *DNAAF3* | 19 | 0.17 | 0.11 | -0.06 | 5.63E-05 | -0.04 | 9.31E-09 |
| cg22744079 | *DCHS2* | 4 | 0.59 | 0.67 | 0.08 | 6.30E-05 | 0.08 | 1.76E-13 |
| cg26052357 |  | 1 | 0.74 | 0.78 | 0.05 | 6.87E-05 | 0.05 | 3.25E-09 |
| cg15108991 | *S100A12* | 1 | 0.63 | 0.61 | -0.02 | 6.97E-05 | -0.02 | 7.74E-11 |
| cg15035382 | *CNTD2* | 19 | 0.82 | 0.78 | -0.04 | 7.62E-05 | -0.07 | 1.20E-15 |
| cg09779044 | *CKAP5* | 11 | 0.88 | 0.87 | -0.01 | 9.83E-05 | -0.02 | 1.01E-10 |
| cg07462804 | *PRDM8* | 4 | 0.05 | 0.05 | 0.01 | 1.18E-04 | 0.01 | 1.55E-11 |
| cg06606198 | *DDX55* | 12 | 0.05 | 0.04 | -0.01 | 1.26E-04 | -0.01 | 9.33E-13 |
| cg26715042 | *CRISP2* | 6 | 0.16 | 0.21 | 0.05 | 1.37E-04 | 0.04 | 8.75E-10 |
| cg10448227 | *PRSS30P* | 16 | 0.38 | 0.34 | -0.05 | 1.48E-04 | -0.05 | 1.31E-09 |
| cg25997306 |  | 2 | 0.16 | 0.22 | 0.06 | 1.54E-04 | 0.05 | 2.52E-12 |
| cg19760250 | *C17orf98* | 17 | 0.87 | 0.91 | 0.04 | 1.69E-04 | 0.06 | 9.15E-09 |
| cg14825413 | *GLI4* | 8 | 0.36 | 0.26 | -0.10 | 1.78E-04 | -0.07 | 1.80E-08 |
| cg21535947 | *NUPL1* | 13 | 0.31 | 0.38 | 0.07 | 1.89E-04 | 0.07 | 2.22E-16 |
| cg13040392 |  | 9 | 0.60 | 0.55 | -0.05 | 2.16E-04 | -0.06 | 1.68E-09 |
| cg18795569 | *SMIM24* | 19 | 0.45 | 0.41 | -0.04 | 2.19E-04 | -0.06 | 7.13E-15 |
| cg08151623 |  | 16 | 0.50 | 0.42 | -0.08 | 2.30E-04 | -0.07 | 3.45E-12 |
| cg14997592 | *CRISP2* | 6 | 0.30 | 0.36 | 0.06 | 2.49E-04 | 0.07 | 5.85E-10 |
| cg26838023 | *LINC00028; REM1* | 20 | 0.67 | 0.71 | 0.04 | 2.58E-04 | 0.04 | 1.26E-09 |
| cg16547629 | *RNF220* | 1 | 0.20 | 0.23 | 0.03 | 2.82E-04 | 0.05 | 2.31E-13 |
| cg05330360 | *ZPBP2* | 17 | 0.44 | 0.49 | 0.05 | 2.86E-04 | 0.07 | 1.07E-11 |
| cg03314840 | *PKLR* | 1 | 0.52 | 0.56 | 0.04 | 3.06E-04 | 0.04 | 5.52E-08 |
| cg08532057 | *NUPL1* | 13 | 0.19 | 0.25 | 0.06 | 3.33E-04 | 0.08 | 3.89E-15 |
| cg10766585 | *SOD3* | 4 | 0.55 | 0.60 | 0.06 | 3.59E-04 | 0.05 | 1.73E-09 |
| cg01185530 | *DNAJC3* | 13 | 0.06 | 0.07 | 0.01 | 4.28E-04 | 0.01 | 5.85E-08 |
| cg22857963 | *GLI4* | 8 | 0.27 | 0.19 | -0.09 | 4.32E-04 | -0.07 | 1.16E-08 |
| cg14373579 | *LOC100272217; FUBP3* | 9 | 0.07 | 0.08 | 0.01 | 4.60E-04 | 0.01 | 1.94E-08 |
| cg04595372 | *CRISP2* | 6 | 0.34 | 0.39 | 0.05 | 5.28E-04 | 0.06 | 4.22E-12 |
| cg13161852 |  | 15 | 0.80 | 0.82 | 0.02 | 5.34E-04 | 0.02 | 7.19E-09 |
| cg27362048 | *APOA5* | 11 | 0.80 | 0.84 | 0.03 | 5.57E-04 | 0.06 | 3.24E-11 |
| cg10776186 | *NUPL1* | 13 | 0.62 | 0.67 | 0.04 | 6.61E-04 | 0.04 | 2.53E-13 |
| cg20118422 | *PPIB* | 15 | 0.06 | 0.07 | 0.01 | 6.83E-04 | 0.01 | 2.59E-08 |
| cg02093808 |  | 4 | 0.23 | 0.27 | 0.05 | 6.83E-04 | 0.05 | 1.79E-12 |
| cg01006802 | *PRSS30P* | 16 | 0.49 | 0.40 | -0.09 | 6.89E-04 | -0.10 | 2.73E-12 |
| cg25390787 | *CRISP2* | 6 | 0.20 | 0.25 | 0.06 | 6.94E-04 | 0.04 | 4.27E-08 |
| cg08942800 | *CRISP2* | 6 | 0.31 | 0.36 | 0.05 | 6.95E-04 | 0.09 | 7.47E-11 |
| cg19160878 | *GTF3C3* | 2 | 0.81 | 0.83 | 0.02 | 7.57E-04 | 0.03 | 9.45E-12 |
| cg00655923 |  | 7 | 0.64 | 0.62 | -0.03 | 7.77E-04 | -0.04 | 1.48E-13 |
| cg07645761 | *PRSS30P* | 16 | 0.55 | 0.45 | -0.10 | 8.01E-04 | -0.11 | 1.09E-13 |
| cg01076129 | *CRISP2* | 6 | 0.39 | 0.44 | 0.05 | 8.06E-04 | 0.06 | 2.96E-09 |
| cg20047230 | *CP110* | 16 | 0.12 | 0.13 | 0.02 | 8.60E-04 | 0.03 | 1.06E-07 |
| cg01604592 | *DMAP1* | 1 | 0.04 | 0.04 | 0.00 | 8.73E-04 | 0.01 | 4.11E-11 |
| cg20851828 |  | 6 | 0.69 | 0.77 | 0.07 | 9.09E-04 | 0.07 | 5.02E-09 |
| cg08652337 | *ARF5* | 7 | 0.04 | 0.04 | 0.01 | 9.98E-04 | 0.00 | 6.20E-09 |

**SUPPLEMENTARY TABLE 10 – The twenty top-ranked autosomal sex-associated DMRs identified in the developing fetal brain.** Chromosomal coordinates correspond to human genome build Feb. 2009 (GRCh37/hg19). A full list of sex-associated DMRs is available for download from our laboratory website (<http://epigenetics.iop.kcl.ac.uk/fetalbrain/1099_sex_DMRs.csv>). See also **Supplementary Figure 11**.

| **Chr** | **Start** | **End** | **Size (bp)** | **Probes (n)** | **p value** | **Gene** |
| --- | --- | --- | --- | --- | --- | --- |
| 13 | 49796387 | 49796490 | 103 | 3 | 2.30E-42 | *MLNR* |
| 17 | 38023993 | 38024637 | 644 | 4 | 3.64E-37 | *ZPBP2* |
| 6 | 5087031 | 5087750 | 719 | 5 | 7.82E-28 | *PPP1R3G* |
| 11 | 116661043 | 116661679 | 636 | 5 | 2.46E-26 | *APOA5* |
| 8 | 57030408 | 57030825 | 417 | 3 | 6.63E-25 |  |
| 6 | 49681178 | 49681392 | 214 | 7 | 4.29E-24 | *CRISP2* |
| 10 | 64572927 | 64573477 | 550 | 4 | 2.92E-20 | *EGR2* |
| 6 | 28601269 | 28601520 | 251 | 11 | 3.34E-20 |  |
| 16 | 2892518 | 2892913 | 395 | 6 | 3.72E-20 | *PRSS30P* |
| 12 | 81110706 | 81111013 | 307 | 5 | 7.65E-20 | *MYF5* |
| 4 | 77341841 | 77342105 | 264 | 4 | 2.41E-19 |  |
| 19 | 3480508 | 3480673 | 165 | 4 | 5.04E-19 | *SMIM24* |
| 6 | 74104097 | 74104869 | 772 | 8 | 2.03E-18 | *DDX43* |
| 1 | 92011732 | 92012737 | 1005 | 9 | 6.21E-18 |  |
| 5 | 23507450 | 23507657 | 207 | 6 | 1.26E-17 | *PRDM9* |
| 8 | 144371537 | 144372052 | 515 | 4 | 6.30E-16 |  |
| 16 | 3242639 | 3242851 | 212 | 3 | 9.98E-16 |  |
| 1 | 149871080 | 149872166 | 1086 | 8 | 3.98E-15 | *BOLA1* |
| 1 | 32741454 | 32741620 | 166 | 4 | 2.92E-14 | *LCK* |
| 19 | 19626251 | 19626822 | 571 | 8 | 3.94E-13 | *NDUFA13, TSSK6* |

**SUPPLEMENTARY TABLE 11 – The twenty top-ranked autosomal dDMRs with sex-specific developmental trajectories in fetal brain.** Chromosomal coordinates correspond to human genome build Feb. 2009 (GRCh37/hg19). A complete list of all significant sex-specific dDMRs is available for download from our laboratory website (<http://epigenetics.iop.kcl.ac.uk/fetalbrain/66_age_sex_DMRs.csv>). See also **Supplementary Figure 13**.

| **Chr** | **Start** | **End** | **Size (bp)** | **Probes (n)** | **p value** | **Gene** |
| --- | --- | --- | --- | --- | --- | --- |
| 7 | 640338 | 640821 | 483 | 5 | 5.19E-16 | *PRKAR1B* |
| 7 | 632926 | 633203 | 277 | 3 | 2.45E-12 | *PRKAR1B* |
| 20 | 18122816 | 18122895 | 79 | 4 | 4.56E-12 | *CSRP2BP; PET117* |
| 8 | 1885886 | 1885998 | 112 | 3 | 7.16E-12 | *ARHGEF10* |
| 22 | 46473039 | 46473344 | 305 | 3 | 2.07E-10 |  |
| 1 | 10509682 | 10510500 | 818 | 9 | 2.64E-10 | *CORT; APITD1* |
| 1 | 221055401 | 221055965 | 564 | 6 | 1.37E-07 | *HLX* |
| 15 | 67356310 | 67356943 | 633 | 5 | 2.93E-07 | *SMAD3* |
| 3 | 35785041 | 35785378 | 337 | 3 | 3.86E-07 | *MIR128-2; ARPP21* |
| 19 | 42546648 | 42546978 | 330 | 4 | 5.37E-07 | *GRIK5* |
| 1 | 119532056 | 119532926 | 870 | 13 | 8.99E-07 | *TBX15* |
| 8 | 618674 | 619023 | 349 | 4 | 1.67E-06 | *ERICH1* |
| 11 | 45928805 | 45928989 | 184 | 7 | 1.74E-06 | *C11orf94* |
| 5 | 83016630 | 83017185 | 555 | 7 | 1.89E-06 | *HAPLN1* |
| 22 | 17646207 | 17646264 | 57 | 3 | 1.95E-06 | *CECR5-AS1; CECR5* |
| 2 | 240723946 | 240724177 | 231 | 3 | 3.53E-06 |  |
| 15 | 70767183 | 70767650 | 467 | 4 | 9.91E-06 |  |
| 14 | 93419016 | 93419555 | 539 | 6 | 4.12E-05 | *ITPK1* |
| 1 | 217309794 | 217310384 | 590 | 5 | 6.65E-05 | *ESRRG* |
| 7 | 101558400 | 101558699 | 299 | 5 | 6.77E-05 | *CUX1* |

**SUPPLEMENTARY TABLE 12 – Co-methylation modules in the developing fetal brain.** A total of 22 modules were identified by WGCNA analysis. Modules are shown alongside their assigned color and ordered by module size. Shown for each module is the module correlation with age and sex. DPC = days post conception.

| **Module** | **Color label** | **Probes** | **Genes associated with module** | **Un-annotated probes** | **XY probes (%)** | **Correlation with DPC**  **(p value)** | **Correlation with sex**  **(p value)** |
| --- | --- | --- | --- | --- | --- | --- | --- |
| 1 | turquoise | 84079 | 16834 | 21707 | 821 (0.98) | 0.19 (9.87E-03) | 0.01 (8.65E-01) |
| 2 | blue | 75124 | 14667 | 9786 | 205 (0.27) | -0.14 (6.44E-02) | -0.13 (9.43E-02) |
| 3 | brown | 24002 | 6396 | 7801 | 116 (0.48) | 0.54 (5.63E-15) | -0.02 (7.69E-01) |
| 4 | yellow | 21999 | 7181 | 6751 | 144 (0.65) | -0.59 (3.94E-18) | -0.04 (6.08E-01) |
| 5 | green | 10647 | 4648 | 2556 | 18 (0.17) | -0.12 (9.72E-02) | -0.16 (2.80E-02) |
| 6 | red | 5352 | 2087 | 1946 | 20 (0.37) | -0.23 (1.68E-03) | -0.11 (1.29E-01) |
| 7 | black | 4956 | 631 | 575 | 4877 (98.41) | 0.01 (8.75E-01) | 0.99 (7.41E-172) |
| 8 | pink | 4223 | 2263 | 1310 | 11 (0.26) | 0.47 (3.84E-11) | -0.06 (4.38E-01) |
| 9 | magenta | 4222 | 3655 | 400 | 7 (0.17) | -0.21 (5.64E-03) | -0.39 (9.43E-08) |
| 10 | purple | 2838 | 2600 | 231 | 3 (0.11) | 0.06 (3.99E-01) | 0.03 (7.33E-01) |
| 11 | greenyellow | 2784 | 1907 | 724 | 17 (0.61) | -0.32 (9.86E-06) | -0.04 (6.40E-01) |
| 12 | tan | 2740 | 1386 | 851 | 11 (0.40) | 0.33 (4.81E-06) | -0.12 (9.79E-02) |
| 13 | salmon | 2649 | 1368 | 794 | 15 (0.57) | 0.22 (2.56E-03) | 0.10 (1.72E-01) |
| 14 | cyan | 2066 | 1898 | 276 | 1 (0.05) | -0.22 (3.42E-03) | -0.32 (1.73E-05) |
| 15 | midnightblue | 1807 | 982 | 505 | 5 (0.28) | 0.12 (9.63E-02) | 0.00 (9.48E-01) |
| 16 | lightcyan | 1795 | 614 | 330 | 1733 (96.55) | -0.01 (9.40E-01) | -0.99 (1.96E-143) |
| 17 | grey60 | 1741 | 1171 | 405 | 2 (0.11) | -0.52 (1.59E-13) | -0.01 (9.27E-01) |
| 18 | lightgreen | 1149 | 629 | 395 | 3 (0.26) | -0.05 (5.23E-01) | -0.05 (4.89E-01) |
| 19 | lightyellow | 930 | 592 | 256 | 0 (0.00) | -0.22 (3.31E-03) | -0.20 (7.42E-03) |
| 20 | royalblue | 884 | 899 | 65 | 1 (0.11) | 0.05 (4.91E-01) | 0.08 (3.09E-01) |
| 21 | darkred | 646 | 393 | 211 | 1 (0.15) | 0.31 (2.30E-05) | -0.04 (5.70E-01) |
| 22 | darkgreen | 599 | 308 | 233 | 3 (0.50) | -0.45 (1.61E-10) | -0.01 (9.46E-01) |

**SUPPLEMENTARY TABLE 13 – Gene ontology pathways significantly enriched (Bonferroni p < 0.05) in the brown WGCNA module,** which is strongly associated with fetal brain development.

| **Rank** | **GO category** | **ID** | **Total probes in pathway** | **dDMPs in pathway** | **Odds ratio** | **p value** | **Bonferroni-corrected p value** |
| --- | --- | --- | --- | --- | --- | --- | --- |
| 1 | epithalamus development | GO:0021538 | 52 | 19 | 78.18 | 3.79E-28 | 4.37E-24 |
| 2 | habenula development | GO:0021986 | 52 | 19 | 78.18 | 3.79E-28 | 4.37E-24 |
| 3 | general adaptation syndrome | GO:0051866 | 56 | 19 | 72.57 | 1.23E-27 | 1.42E-23 |
| 4 | central nervous system neuron axonogenesis | GO:0021955 | 940 | 36 | 8.26 | 9.70E-21 | 1.12E-16 |
| 5 | central nervous system projection neuron axonogenesis | GO:0021952 | 753 | 32 | 9.15 | 7.42E-20 | 8.55E-16 |
| 6 | dopamine biosynthetic process | GO:0042416 | 219 | 19 | 18.55 | 1.17E-17 | 1.35E-13 |
| 7 | response to insecticide | GO:0017085 | 230 | 19 | 17.65 | 2.73E-17 | 3.14E-13 |
| 8 | central nervous system neuron development | GO:0021954 | 1745 | 42 | 5.20 | 7.15E-17 | 8.25E-13 |
| 9 | regulation of respiratory gaseous exchange | GO:0043576 | 451 | 23 | 10.93 | 2.71E-16 | 3.12E-12 |
| 10 | axon part | GO:0033267 | 5046 | 73 | 3.16 | 3.18E-16 | 3.67E-12 |
| 11 | regulation of dopamine metabolic process | GO:0042053 | 266 | 19 | 15.27 | 3.31E-16 | 3.82E-12 |
| 12 | regulation of catecholamine metabolic process | GO:0042069 | 282 | 19 | 14.40 | 9.01E-16 | 1.04E-11 |
| 13 | neuron maturation | GO:0042551 | 863 | 28 | 6.97 | 9.78E-15 | 1.13E-10 |
| 14 | steroid hormone receptor activity | GO:0003707 | 1479 | 35 | 5.09 | 4.24E-14 | 4.89E-10 |
| 15 | nervous system development | GO:0007399 | 47582 | 333 | 1.61 | 1.25E-13 | 1.44E-09 |
| 16 | catecholamine biosynthetic process | GO:0042423 | 385 | 19 | 10.55 | 1.78E-13 | 2.05E-09 |
| 17 | catechol-containing compound biosynthetic process | GO:0009713 | 385 | 19 | 10.55 | 1.78E-13 | 2.05E-09 |
| 18 | dopaminergic neuron differentiation | GO:0071542 | 397 | 19 | 10.22 | 2.98E-13 | 3.44E-09 |
| 19 | neurofilament cytoskeleton | GO:0060053 | 652 | 23 | 7.55 | 4.43E-13 | 5.11E-09 |
| 20 | phenol-containing compound biosynthetic process | GO:0046189 | 604 | 22 | 7.79 | 7.75E-13 | 8.93E-09 |
| 21 | central nervous system neuron differentiation | GO:0021953 | 4784 | 63 | 2.86 | 1.82E-12 | 2.10E-08 |
| 22 | somatic stem cell maintenance | GO:0035019 | 1841 | 36 | 4.21 | 3.94E-12 | 4.54E-08 |
| 23 | neurofilament | GO:0005883 | 530 | 20 | 8.07 | 4.55E-12 | 5.25E-08 |
| 24 | single-organism developmental process | GO:0044767 | 98913 | 593 | 1.44 | 6.62E-12 | 7.63E-08 |
| 25 | dopamine metabolic process | GO:0042417 | 482 | 19 | 8.42 | 7.54E-12 | 8.69E-08 |
| 26 | developmental process | GO:0032502 | 99818 | 597 | 1.44 | 8.09E-12 | 9.32E-08 |
| 27 | response to amphetamine | GO:0001975 | 632 | 21 | 7.11 | 1.32E-11 | 1.53E-07 |
| 28 | positive regulation of gene expression | GO:0010628 | 27884 | 211 | 1.70 | 1.67E-11 | 1.93E-07 |
| 29 | positive regulation of transcription | GO:0045893 | 25980 | 198 | 1.70 | 4.36E-11 | 5.03E-07 |
| 30 | cell differentiation | GO:0030154 | 63755 | 407 | 1.48 | 4.59E-11 | 5.29E-07 |
| 31 | single-multicellular organism process | GO:0044707 | 115095 | 667 | 1.40 | 6.44E-11 | 7.43E-07 |
| 32 | negative regulation of cell differentiation | GO:0045596 | 12800 | 116 | 1.99 | 6.54E-11 | 7.55E-07 |
| 33 | cell morphogenesis involved in differentiation | GO:0000904 | 17845 | 148 | 1.83 | 6.99E-11 | 8.06E-07 |
| 34 | multicellular organismal process | GO:0032501 | 118290 | 682 | 1.40 | 8.12E-11 | 9.36E-07 |
| 35 | response to amine | GO:0014075 | 791 | 22 | 5.95 | 1.16E-10 | 1.34E-06 |
| 36 | central nervous system development | GO:0007417 | 19618 | 158 | 1.78 | 1.21E-10 | 1.40E-06 |
| 37 | multicellular organismal development | GO:0007275 | 91270 | 546 | 1.41 | 1.41E-10 | 1.63E-06 |
| 38 | axon | GO:0030424 | 8715 | 87 | 2.18 | 2.09E-10 | 2.41E-06 |
| 39 | negative regulation of GTPase activity | GO:0034260 | 1535 | 30 | 4.19 | 2.39E-10 | 2.76E-06 |
| 40 | sequence-specific DNA binding | GO:0043565 | 18374 | 149 | 1.79 | 2.74E-10 | 3.16E-06 |
| 41 | regulation of GTPase activity | GO:0043087 | 10415 | 98 | 2.06 | 3.20E-10 | 3.69E-06 |
| 42 | regulation of GTP catabolic process | GO:0033124 | 10432 | 98 | 2.05 | 3.48E-10 | 4.01E-06 |
| 43 | anatomical structure development | GO:0048856 | 90274 | 538 | 1.40 | 3.96E-10 | 4.56E-06 |
| 44 | cell development | GO:0048468 | 35622 | 249 | 1.57 | 4.00E-10 | 4.61E-06 |
| 45 | brain development | GO:0007420 | 14937 | 127 | 1.87 | 4.00E-10 | 4.61E-06 |
| 46 | positive regulation of RNA metabolic process | GO:0051254 | 27427 | 202 | 1.64 | 4.48E-10 | 5.16E-06 |
| 47 | forebrain development | GO:0030900 | 8712 | 86 | 2.15 | 4.49E-10 | 5.18E-06 |
| 48 | system development | GO:0048731 | 80128 | 486 | 1.42 | 4.51E-10 | 5.21E-06 |
| 49 | diencephalon development | GO:0021536 | 2102 | 35 | 3.58 | 5.09E-10 | 5.87E-06 |
| 50 | axonogenesis | GO:0007409 | 14089 | 121 | 1.89 | 5.99E-10 | 6.90E-06 |
| 51 | amygdala development | GO:0021764 | 54 | 8 | 31.46 | 6.43E-10 | 7.42E-06 |
| 52 | multicellular organismal response to stress | GO:0033555 | 1732 | 31 | 3.84 | 9.40E-10 | 1.08E-05 |
| 53 | long-term synaptic potentiation | GO:0060291 | 978 | 23 | 5.03 | 1.02E-09 | 1.18E-05 |
| 54 | axon development | GO:0061564 | 14415 | 122 | 1.86 | 1.18E-09 | 1.36E-05 |
| 55 | regulation of cell differentiation | GO:0045595 | 28300 | 205 | 1.61 | 1.23E-09 | 1.42E-05 |
| 56 | neural crest formation | GO:0014029 | 60 | 8 | 28.32 | 1.37E-09 | 1.58E-05 |
| 57 | adult behavior | GO:0030534 | 3488 | 46 | 2.84 | 1.67E-09 | 1.93E-05 |
| 58 | regulation of nucleoside metabolic process | GO:0009118 | 11073 | 100 | 1.97 | 1.70E-09 | 1.96E-05 |
| 59 | negative regulation of myeloid leukocyte differentiation | GO:0002762 | 1380 | 27 | 4.19 | 1.81E-09 | 2.09E-05 |
| 60 | negative regulation of B cell differentiation | GO:0045578 | 94 | 9 | 20.33 | 1.89E-09 | 2.18E-05 |
| 61 | cellular response to oxidative stress | GO:0034599 | 2678 | 39 | 3.13 | 2.12E-09 | 2.44E-05 |
| 62 | cellular developmental process | GO:0048869 | 65887 | 408 | 1.42 | 2.15E-09 | 2.48E-05 |
| 63 | regulation of generation of precursor metabolites and energy | GO:0043467 | 2338 | 36 | 3.31 | 2.16E-09 | 2.49E-05 |
| 64 | cell morphogenesis involved in neuron differentiation | GO:0048667 | 14897 | 124 | 1.83 | 2.22E-09 | 2.56E-05 |
| 65 | O-fucosylpeptide 3-beta-N-acetylglucosaminyltransferase activity | GO:0033829 | 65 | 8 | 26.15 | 2.44E-09 | 2.82E-05 |
| 66 | regulation of purine nucleotide catabolic process | GO:0033121 | 11049 | 99 | 1.96 | 3.04E-09 | 3.50E-05 |
| 67 | regulation of Wnt signaling pathway involved in dorsal/ventral axis specification | GO:2000053 | 67 | 8 | 25.36 | 3.04E-09 | 3.51E-05 |
| 68 | regulation of nucleotide catabolic process | GO:0030811 | 11064 | 99 | 1.95 | 3.25E-09 | 3.74E-05 |
| 69 | anatomical structure morphogenesis | GO:0009653 | 48079 | 312 | 1.47 | 4.02E-09 | 4.63E-05 |
| 70 | regulation of cellular catabolic process | GO:0031329 | 16715 | 134 | 1.76 | 4.61E-09 | 5.31E-05 |
| 71 | transcription initiation from RNA polymerase II promoter | GO:0006367 | 4524 | 53 | 2.53 | 5.23E-09 | 6.03E-05 |
| 72 | regulation of developmental process | GO:0050793 | 37948 | 256 | 1.51 | 5.50E-09 | 6.34E-05 |
| 73 | direct ligand regulated sequence-specific DNA binding transcription factor activity | GO:0098531 | 1360 | 26 | 4.09 | 5.68E-09 | 6.54E-05 |
| 74 | ligand-activated sequence-specific DNA binding RNA polymerase II transcription factor activity | GO:0004879 | 1360 | 26 | 4.09 | 5.68E-09 | 6.54E-05 |
| 75 | cellular response to extracellular stimulus | GO:0031668 | 2903 | 40 | 2.96 | 5.93E-09 | 6.84E-05 |
| 76 | regulation of canonical Wnt signaling pathway involved in controlling type B pancreatic cell proliferation | GO:2000079 | 74 | 8 | 22.94 | 6.24E-09 | 7.20E-05 |
| 77 | neurogenesis | GO:0022008 | 34232 | 235 | 1.53 | 6.44E-09 | 7.43E-05 |
| 78 | neuron projection morphogenesis | GO:0048812 | 15217 | 124 | 1.79 | 7.33E-09 | 8.46E-05 |
| 79 | positive regulation of nucleobase-containing compound metabolic process | GO:0045935 | 30549 | 214 | 1.56 | 7.41E-09 | 8.55E-05 |
| 80 | negative regulation of developmental process | GO:0051093 | 15384 | 125 | 1.78 | 7.47E-09 | 8.61E-05 |
| 81 | organ development | GO:0048513 | 55700 | 351 | 1.43 | 7.59E-09 | 8.76E-05 |
| 82 | positive regulation of macromolecule biosynthetic process | GO:0010557 | 29508 | 208 | 1.57 | 7.77E-09 | 8.96E-05 |
| 83 | catecholamine metabolic process | GO:0006584 | 745 | 19 | 5.44 | 8.10E-09 | 9.34E-05 |
| 84 | catechol-containing compound metabolic process | GO:0009712 | 745 | 19 | 5.44 | 8.10E-09 | 9.34E-05 |
| 85 | negative regulation of leukocyte differentiation | GO:1902106 | 1917 | 31 | 3.47 | 9.32E-09 | 1.07E-04 |
| 86 | positive regulation of nitrogen compound metabolic process | GO:0051173 | 31014 | 216 | 1.55 | 9.81E-09 | 1.13E-04 |
| 87 | regulation of cell morphogenesis involved in differentiation | GO:0010769 | 5845 | 62 | 2.29 | 1.02E-08 | 1.18E-04 |
| 88 | synapse assembly | GO:0007416 | 1521 | 27 | 3.80 | 1.31E-08 | 1.51E-04 |
| 89 | negative regulation of B cell activation | GO:0050869 | 461 | 15 | 6.93 | 1.39E-08 | 1.61E-04 |
| 90 | positive regulation of biosynthetic process | GO:0009891 | 31902 | 220 | 1.54 | 1.52E-08 | 1.75E-04 |
| 91 | cell morphogenesis | GO:0000902 | 21833 | 162 | 1.64 | 1.85E-08 | 2.13E-04 |
| 92 | stem cell maintenance | GO:0019827 | 2911 | 39 | 2.88 | 1.89E-08 | 2.18E-04 |
| 93 | adult locomotory behavior | GO:0008344 | 2234 | 33 | 3.17 | 2.57E-08 | 2.96E-04 |
| 94 | cellular component morphogenesis | GO:0032989 | 23154 | 169 | 1.61 | 2.61E-08 | 3.00E-04 |
| 95 | negative regulation of fibroblast apoptotic process | GO:2000270 | 130 | 9 | 14.71 | 2.65E-08 | 3.05E-04 |
| 96 | sequence-specific DNA binding RNA polymerase II transcription factor activity | GO:0000981 | 7012 | 69 | 2.13 | 2.70E-08 | 3.11E-04 |
| 97 | positive regulation of cellular biosynthetic process | GO:0031328 | 31602 | 217 | 1.53 | 2.70E-08 | 3.11E-04 |
| 98 | positive regulation of synaptic transmission | GO:0050806 | 1798 | 29 | 3.45 | 2.91E-08 | 3.36E-04 |
| 99 | post-embryonic development | GO:0009791 | 2599 | 36 | 2.97 | 2.95E-08 | 3.40E-04 |
| 100 | terminal bouton | GO:0043195 | 1929 | 30 | 3.33 | 3.69E-08 | 4.25E-04 |
| 101 | skeletal system morphogenesis | GO:0048705 | 5784 | 60 | 2.24 | 3.76E-08 | 4.34E-04 |
| 102 | compartment pattern specification | GO:0007386 | 184 | 10 | 11.55 | 3.93E-08 | 4.54E-04 |
| 103 | positive regulation of cellular process | GO:0048522 | 74825 | 445 | 1.36 | 4.73E-08 | 5.46E-04 |
| 104 | white fat cell differentiation | GO:0050872 | 1114 | 22 | 4.22 | 4.75E-08 | 5.47E-04 |
| 105 | regulation of planar cell polarity pathway involved in axis elongation | GO:2000040 | 98 | 8 | 17.33 | 4.78E-08 | 5.51E-04 |
| 106 | negative regulation of planar cell polarity pathway involved in axis elongation | GO:2000041 | 98 | 8 | 17.33 | 4.78E-08 | 5.51E-04 |
| 107 | DNA-templated transcription | GO:0006352 | 5012 | 54 | 2.32 | 5.55E-08 | 6.40E-04 |
| 108 | positive regulation of non-canonical Wnt signaling pathway | GO:2000052 | 143 | 9 | 13.36 | 5.74E-08 | 6.61E-04 |
| 109 | neuron migration | GO:0001764 | 3042 | 39 | 2.75 | 5.79E-08 | 6.68E-04 |
| 110 | positive regulation of transmission of nerve impulse | GO:0051971 | 1860 | 29 | 3.34 | 5.87E-08 | 6.76E-04 |
| 111 | axis elongation involved in somitogenesis | GO:0090245 | 101 | 8 | 16.81 | 5.95E-08 | 6.86E-04 |
| 112 | positive regulation of neurological system process | GO:0031646 | 2091 | 31 | 3.18 | 6.21E-08 | 7.16E-04 |
| 113 | negative regulation of cellular process | GO:0048523 | 69297 | 415 | 1.37 | 8.02E-08 | 9.25E-04 |
| 114 | regulation of granulocyte differentiation | GO:0030852 | 960 | 20 | 4.45 | 8.31E-08 | 9.58E-04 |
| 115 | negative regulation of transmembrane receptor protein serine/threonine kinase signaling pathway | GO:0090101 | 2964 | 38 | 2.75 | 8.52E-08 | 9.82E-04 |
| 116 | negative regulation of hydrolase activity | GO:0051346 | 5950 | 60 | 2.18 | 9.68E-08 | 1.12E-03 |
| 117 | cellular response to external stimulus | GO:0071496 | 4033 | 46 | 2.45 | 1.11E-07 | 1.28E-03 |
| 118 | negative regulation of granulocyte differentiation | GO:0030853 | 796 | 18 | 4.82 | 1.13E-07 | 1.31E-03 |
| 119 | regulation of cell communication | GO:0010646 | 55745 | 343 | 1.39 | 1.37E-07 | 1.58E-03 |
| 120 | negative regulation of cell development | GO:0010721 | 3671 | 43 | 2.52 | 1.42E-07 | 1.64E-03 |
| 121 | regulation of cellular respiration | GO:0043457 | 723 | 17 | 5.01 | 1.48E-07 | 1.71E-03 |
| 122 | somite development | GO:0061053 | 1619 | 26 | 3.43 | 1.61E-07 | 1.85E-03 |
| 123 | transport vesicle membrane | GO:0030658 | 2425 | 33 | 2.92 | 1.61E-07 | 1.86E-03 |
| 124 | negative regulation of myeloid cell differentiation | GO:0045638 | 2196 | 31 | 3.02 | 1.76E-07 | 2.03E-03 |
| 125 | prostate glandular acinus morphogenesis | GO:0060526 | 219 | 10 | 9.70 | 1.85E-07 | 2.13E-03 |
| 126 | prostate epithelial cord arborization involved in prostate glandular acinus morphogenesis | GO:0060527 | 219 | 10 | 9.70 | 1.85E-07 | 2.13E-03 |
| 127 | behavior | GO:0007610 | 11267 | 94 | 1.81 | 1.88E-07 | 2.17E-03 |
| 128 | phenol-containing compound metabolic process | GO:0018958 | 1418 | 24 | 3.62 | 1.90E-07 | 2.19E-03 |
| 129 | locomotory behavior | GO:0007626 | 4660 | 50 | 2.31 | 1.92E-07 | 2.21E-03 |
| 130 | generation of neurons | GO:0048699 | 32093 | 215 | 1.48 | 1.96E-07 | 2.26E-03 |
| 131 | axon terminus | GO:0043679 | 2569 | 34 | 2.84 | 1.97E-07 | 2.27E-03 |
| 132 | positive regulation of fibroblast apoptotic process | GO:2000271 | 120 | 8 | 14.16 | 2.07E-07 | 2.38E-03 |
| 133 | transcriptional repressor complex | GO:0017053 | 3084 | 38 | 2.64 | 2.23E-07 | 2.57E-03 |
| 134 | cell fate commitment | GO:0045165 | 6691 | 64 | 2.07 | 2.25E-07 | 2.59E-03 |
| 135 | cellular response to X-ray | GO:0071481 | 122 | 8 | 13.92 | 2.33E-07 | 2.69E-03 |
| 136 | somitogenesis | GO:0001756 | 1436 | 24 | 3.57 | 2.37E-07 | 2.73E-03 |
| 137 | negative regulation of neuron apoptotic process | GO:0043524 | 2967 | 37 | 2.68 | 2.41E-07 | 2.78E-03 |
| 138 | regulation of catabolic process | GO:0009894 | 18468 | 137 | 1.62 | 2.57E-07 | 2.96E-03 |
| 139 | cellular response to heparin | GO:0071504 | 124 | 8 | 13.70 | 2.62E-07 | 3.02E-03 |
| 140 | middle ear morphogenesis | GO:0042474 | 583 | 15 | 5.48 | 2.63E-07 | 3.03E-03 |
| 141 | regulation of purine nucleotide metabolic process | GO:1900542 | 13629 | 108 | 1.73 | 2.68E-07 | 3.09E-03 |
| 142 | transmembrane receptor protein phosphatase activity | GO:0019198 | 1672 | 26 | 3.33 | 2.91E-07 | 3.36E-03 |
| 143 | transmembrane receptor protein tyrosine phosphatase activity | GO:0005001 | 1672 | 26 | 3.33 | 2.91E-07 | 3.36E-03 |
| 144 | regulation of nucleotide metabolic process | GO:0006140 | 13660 | 108 | 1.72 | 2.98E-07 | 3.43E-03 |
| 145 | intraspecies interaction between organisms | GO:0051703 | 1145 | 21 | 3.92 | 3.08E-07 | 3.56E-03 |
| 146 | social behavior | GO:0035176 | 1145 | 21 | 3.92 | 3.08E-07 | 3.56E-03 |
| 147 | positive regulation of biological process | GO:0048518 | 82374 | 477 | 1.33 | 3.24E-07 | 3.73E-03 |
| 148 | regulation of signaling | GO:0023051 | 55564 | 339 | 1.37 | 3.77E-07 | 4.35E-03 |
| 149 | female gonad development | GO:0008585 | 1825 | 27 | 3.16 | 4.46E-07 | 5.14E-03 |
| 150 | signaling receptor activity | GO:0038023 | 18334 | 135 | 1.61 | 4.67E-07 | 5.39E-03 |
| 151 | negative regulation of neuron death | GO:1901215 | 3065 | 37 | 2.59 | 5.15E-07 | 5.94E-03 |
| 152 | neuron differentiation | GO:0030182 | 25006 | 173 | 1.52 | 5.37E-07 | 6.19E-03 |
| 153 | cell projection morphogenesis | GO:0048858 | 17721 | 131 | 1.62 | 5.71E-07 | 6.59E-03 |
| 154 | sensory organ development | GO:0007423 | 12220 | 98 | 1.74 | 5.83E-07 | 6.72E-03 |
| 155 | dorsal/ventral axis specification | GO:0009950 | 623 | 15 | 5.13 | 5.90E-07 | 6.81E-03 |
| 156 | convergent extension involved in axis elongation | GO:0060028 | 139 | 8 | 12.22 | 5.96E-07 | 6.87E-03 |
| 157 | ionotropic glutamate receptor binding | GO:0035255 | 803 | 17 | 4.51 | 6.11E-07 | 7.04E-03 |
| 158 | regionalization | GO:0003002 | 7977 | 71 | 1.92 | 6.69E-07 | 7.71E-03 |
| 159 | neuron projection terminus | GO:0044306 | 2975 | 36 | 2.59 | 6.91E-07 | 7.96E-03 |
| 160 | regulation of blood vessel endothelial cell migration | GO:0043535 | 1110 | 20 | 3.84 | 7.61E-07 | 8.78E-03 |
| 161 | synapse organization | GO:0050808 | 3124 | 37 | 2.54 | 7.98E-07 | 9.20E-03 |
| 162 | response to heparin | GO:0071503 | 149 | 8 | 11.40 | 9.80E-07 | 1.13E-02 |
| 163 | GKAP/Homer scaffold activity | GO:0030160 | 333 | 11 | 7.02 | 1.00E-06 | 1.16E-02 |
| 164 | anterior/posterior pattern specification | GO:0009952 | 5373 | 53 | 2.12 | 1.01E-06 | 1.16E-02 |
| 165 | regulation of branching involved in prostate gland morphogenesis | GO:0060687 | 269 | 10 | 7.90 | 1.12E-06 | 1.29E-02 |
| 166 | cell part morphogenesis | GO:0032990 | 17971 | 131 | 1.59 | 1.16E-06 | 1.34E-02 |
| 167 | ovulation cycle | GO:0042698 | 1926 | 27 | 3.00 | 1.21E-06 | 1.39E-02 |
| 168 | cellular response to vitamin D | GO:0071305 | 154 | 8 | 11.03 | 1.24E-06 | 1.43E-02 |
| 169 | ovulation cycle process | GO:0022602 | 1814 | 26 | 3.06 | 1.27E-06 | 1.47E-02 |
| 170 | branching involved in prostate gland morphogenesis | GO:0060442 | 344 | 11 | 6.80 | 1.36E-06 | 1.57E-02 |
| 171 | multi-organism behavior | GO:0051705 | 1823 | 26 | 3.05 | 1.39E-06 | 1.60E-02 |
| 172 | regulation of multicellular organismal process | GO:0051239 | 46832 | 289 | 1.38 | 1.45E-06 | 1.68E-02 |
| 173 | negative regulation of non-canonical Wnt signaling pathway | GO:2000051 | 158 | 8 | 10.75 | 1.49E-06 | 1.72E-02 |
| 174 | regulation of timing of cell differentiation | GO:0048505 | 423 | 12 | 6.03 | 1.53E-06 | 1.76E-02 |
| 175 | regulation of cell morphogenesis | GO:0022604 | 8640 | 74 | 1.85 | 1.53E-06 | 1.77E-02 |
| 176 | regulation of cell development | GO:0060284 | 15356 | 115 | 1.63 | 1.60E-06 | 1.85E-02 |
| 177 | regulation of B cell differentiation | GO:0045577 | 425 | 12 | 6.00 | 1.60E-06 | 1.85E-02 |
| 178 | tongue development | GO:0043586 | 1064 | 19 | 3.81 | 1.62E-06 | 1.86E-02 |
| 179 | cell projection part | GO:0044463 | 16209 | 120 | 1.61 | 1.62E-06 | 1.87E-02 |
| 180 | menstrual cycle phase | GO:0022601 | 160 | 8 | 10.61 | 1.63E-06 | 1.88E-02 |
| 181 | single-organism behavior | GO:0044708 | 8504 | 73 | 1.85 | 1.67E-06 | 1.92E-02 |
| 182 | response to toxic substance | GO:0009636 | 2454 | 31 | 2.70 | 1.71E-06 | 1.97E-02 |
| 183 | positive regulation of metabolic process | GO:0009893 | 50168 | 306 | 1.36 | 1.74E-06 | 2.01E-02 |
| 184 | anterior commissure morphogenesis | GO:0021960 | 163 | 8 | 10.42 | 1.86E-06 | 2.14E-02 |
| 185 | regulation of development | GO:0040034 | 433 | 12 | 5.89 | 1.93E-06 | 2.22E-02 |
| 186 | regulation of fibroblast apoptotic process | GO:2000269 | 222 | 9 | 8.61 | 1.94E-06 | 2.23E-02 |
| 187 | mesodermal cell fate specification | GO:0007501 | 167 | 8 | 10.17 | 2.21E-06 | 2.55E-02 |
| 188 | regulation of glycolysis | GO:0006110 | 888 | 17 | 4.08 | 2.29E-06 | 2.64E-02 |
| 189 | positive regulation of fat cell differentiation | GO:0045600 | 797 | 16 | 4.28 | 2.51E-06 | 2.89E-02 |
| 190 | regulation of cell migration involved in sprouting angiogenesis | GO:0090049 | 369 | 11 | 6.34 | 2.61E-06 | 3.01E-02 |
| 191 | negative regulation of biological process | GO:0048519 | 74155 | 428 | 1.31 | 2.69E-06 | 3.10E-02 |
| 192 | neuron development | GO:0048666 | 20549 | 144 | 1.53 | 2.71E-06 | 3.12E-02 |
| 193 | regulation of Wnt signaling pathway | GO:2000095 | 233 | 9 | 8.20 | 2.83E-06 | 3.26E-02 |
| 194 | forebrain generation of neurons | GO:0021872 | 1666 | 24 | 3.08 | 2.95E-06 | 3.40E-02 |
| 195 | regulation of timing of neuron differentiation | GO:0060164 | 174 | 8 | 9.76 | 2.95E-06 | 3.41E-02 |
| 196 | telencephalon development | GO:0021537 | 5145 | 50 | 2.09 | 3.01E-06 | 3.47E-02 |
| 197 | negative regulation of cell proliferation | GO:0008285 | 13385 | 102 | 1.65 | 3.08E-06 | 3.55E-02 |
| 198 | regulation of hydrolase activity | GO:0051336 | 21489 | 149 | 1.52 | 3.14E-06 | 3.62E-02 |
| 199 | regulation of cellular amine metabolic process | GO:0033238 | 1568 | 23 | 3.13 | 3.52E-06 | 4.06E-02 |
| 200 | regulation of somitogenesis | GO:0014807 | 241 | 9 | 7.93 | 3.69E-06 | 4.25E-02 |
| 201 | neuron projection development | GO:0031175 | 17537 | 126 | 1.57 | 3.73E-06 | 4.30E-02 |
| 202 | positive regulation of macromolecule metabolic process | GO:0010604 | 45693 | 280 | 1.36 | 3.84E-06 | 4.43E-02 |
| 203 | intermediate filament | GO:0005882 | 1934 | 26 | 2.87 | 3.91E-06 | 4.51E-02 |

**SUPPLEMENTARY TABLE 14 - Gene ontology pathways significantly enriched (Bonferroni p < 0.05) in the yellow WGCNA module,** which is strongly associated with fetal brain development.

| **Rank** | **GO category** | **ID** | **Total probes in pathway** | **dDMPs in pathway** | **Odds ratio** | **p value** | **Bonferroni-corrected p value** |
| --- | --- | --- | --- | --- | --- | --- | --- |
| 1 | negative regulation of granulocyte differentiation | GO:0030853 | 796 | 35 | 4.11 | 1.57E-11 | 1.81E-07 |
| 2 | regulation of cellular respiration | GO:0043457 | 723 | 32 | 4.14 | 9.71E-11 | 1.12E-06 |
| 3 | regulation of granulocyte differentiation | GO:0030852 | 960 | 36 | 3.51 | 5.24E-10 | 6.04E-06 |
| 4 | astrocyte differentiation | GO:0048708 | 647 | 29 | 4.19 | 5.43E-10 | 6.26E-06 |
| 5 | astrocyte projection | GO:0097449 | 95 | 12 | 11.76 | 2.06E-09 | 2.38E-05 |
| 6 | glial cell projection | GO:0097386 | 99 | 12 | 11.29 | 3.15E-09 | 3.64E-05 |
| 7 | regulation of glial cell proliferation | GO:0060251 | 500 | 24 | 4.48 | 4.59E-09 | 5.29E-05 |
| 8 | focal adhesion | GO:0005925 | 3506 | 77 | 2.06 | 1.44E-08 | 1.66E-04 |
| 9 | fat cell differentiation | GO:0045444 | 2694 | 64 | 2.23 | 1.53E-08 | 1.77E-04 |
| 10 | white fat cell differentiation | GO:0050872 | 1114 | 36 | 3.02 | 2.09E-08 | 2.41E-04 |
| 11 | cell-substrate adherens junction | GO:0005924 | 3604 | 78 | 2.03 | 2.10E-08 | 2.42E-04 |
| 12 | tongue development | GO:0043586 | 1064 | 35 | 3.07 | 2.15E-08 | 2.48E-04 |
| 13 | adherens junction | GO:0005912 | 5895 | 111 | 1.77 | 3.81E-08 | 4.39E-04 |
| 14 | intermediate filament | GO:0005882 | 1934 | 50 | 2.42 | 4.78E-08 | 5.51E-04 |
| 15 | anchoring junction | GO:0070161 | 6217 | 115 | 1.74 | 5.32E-08 | 6.13E-04 |
| 16 | cell junction | GO:0030054 | 20192 | 298 | 1.41 | 5.43E-08 | 6.26E-04 |
| 17 | cell-substrate junction | GO:0030055 | 3830 | 80 | 1.96 | 5.82E-08 | 6.71E-04 |
| 18 | regulation of Schwann cell proliferation | GO:0010624 | 227 | 15 | 6.16 | 7.42E-08 | 8.56E-04 |
| 19 | negative regulation of transmembrane receptor protein serine/threonine kinase signaling pathway | GO:0090101 | 2964 | 66 | 2.09 | 9.25E-08 | 1.07E-03 |
| 20 | intracellular calcium activated chloride channel activity | GO:0005229 | 167 | 13 | 7.25 | 9.28E-08 | 1.07E-03 |
| 21 | brown fat cell differentiation | GO:0050873 | 1046 | 33 | 2.95 | 1.34E-07 | 1.54E-03 |
| 22 | positive regulation of glial cell proliferation | GO:0060252 | 144 | 12 | 7.76 | 1.45E-07 | 1.67E-03 |
| 23 | somatic stem cell maintenance | GO:0035019 | 1841 | 47 | 2.39 | 1.68E-07 | 1.94E-03 |
| 24 | plasma membrane | GO:0005886 | 82435 | 1020 | 1.22 | 1.85E-07 | 2.13E-03 |
| 25 | long-term synaptic potentiation | GO:0060291 | 978 | 31 | 2.96 | 2.86E-07 | 3.30E-03 |
| 26 | cGMP biosynthetic process | GO:0006182 | 126 | 11 | 8.13 | 3.09E-07 | 3.56E-03 |
| 27 | anion transport | GO:0006820 | 7540 | 130 | 1.63 | 3.15E-07 | 3.64E-03 |
| 28 | growth cone | GO:0030426 | 3155 | 67 | 1.99 | 3.81E-07 | 4.40E-03 |
| 29 | negative regulation of myeloid leukocyte differentiation | GO:0002762 | 1380 | 38 | 2.57 | 4.27E-07 | 4.93E-03 |
| 30 | negative regulation of transforming growth factor beta receptor signaling pathway | GO:0030512 | 2152 | 51 | 2.22 | 4.48E-07 | 5.16E-03 |
| 31 | Bergmann glial cell differentiation | GO:0060020 | 79 | 9 | 10.60 | 4.72E-07 | 5.44E-03 |
| 32 | cell periphery | GO:0071944 | 84157 | 1034 | 1.21 | 5.27E-07 | 6.08E-03 |
| 33 | gliogenesis | GO:0042063 | 3785 | 76 | 1.88 | 5.28E-07 | 6.09E-03 |
| 34 | regulation of the force of heart contraction | GO:0002026 | 515 | 21 | 3.80 | 5.34E-07 | 6.16E-03 |
| 35 | semaphorin receptor complex | GO:0002116 | 272 | 15 | 5.14 | 6.59E-07 | 7.60E-03 |
| 36 | carbon dioxide transport | GO:0015670 | 60 | 8 | 12.40 | 6.94E-07 | 8.00E-03 |
| 37 | regulation of endothelial cell chemotaxis | GO:2001026 | 276 | 15 | 5.06 | 7.84E-07 | 9.04E-03 |
| 38 | site of polarized growth | GO:0030427 | 3247 | 67 | 1.93 | 9.93E-07 | 1.15E-02 |
| 39 | inorganic anion transport | GO:0015698 | 1968 | 47 | 2.23 | 1.01E-06 | 1.16E-02 |
| 40 | negative regulation of cellular response to growth factor stimulus | GO:0090288 | 2922 | 62 | 1.99 | 1.04E-06 | 1.20E-02 |
| 41 | glial cell differentiation | GO:0010001 | 3396 | 69 | 1.90 | 1.16E-06 | 1.34E-02 |
| 42 | nervous system development | GO:0007399 | 47582 | 614 | 1.24 | 1.20E-06 | 1.39E-02 |
| 43 | brush border membrane | GO:0031526 | 958 | 29 | 2.83 | 1.63E-06 | 1.88E-02 |
| 44 | hepatocyte growth factor receptor signaling pathway | GO:0048012 | 94 | 9 | 8.91 | 1.80E-06 | 2.07E-02 |
| 45 | stem cell maintenance | GO:0019827 | 2911 | 61 | 1.96 | 1.85E-06 | 2.14E-02 |
| 46 | regulation of gene silencing by miRNA | GO:0060964 | 189 | 12 | 5.91 | 2.16E-06 | 2.49E-02 |
| 47 | regulation of posttranscriptional gene silencing | GO:0060147 | 189 | 12 | 5.91 | 2.16E-06 | 2.49E-02 |
| 48 | regulation of gene silencing by RNA | GO:0060966 | 189 | 12 | 5.91 | 2.16E-06 | 2.49E-02 |
| 49 | cell body | GO:0044297 | 8676 | 141 | 1.53 | 2.27E-06 | 2.61E-02 |
| 50 | acyl-CoA hydrolase activity | GO:0047617 | 194 | 12 | 5.76 | 2.78E-06 | 3.21E-02 |
| 51 | negative regulation of translation | GO:0040033 | 270 | 14 | 4.83 | 3.03E-06 | 3.50E-02 |
| 52 | regulation of translation | GO:0045974 | 270 | 14 | 4.83 | 3.03E-06 | 3.50E-02 |
| 53 | negative regulation of translation involved in gene silencing by miRNA | GO:0035278 | 270 | 14 | 4.83 | 3.03E-06 | 3.50E-02 |
| 54 | semaphorin receptor activity | GO:0017154 | 354 | 16 | 4.21 | 3.34E-06 | 3.86E-02 |
| 55 | cell adhesion | GO:0007155 | 17833 | 256 | 1.36 | 3.71E-06 | 4.28E-02 |

**SUPPLEMENTARY TABLE 15 - Top-twenty enriched gene ontology pathways in the lightgreen WGCNA module.** This module is not associated with either fetal brain development or sex. No GO categories reach significance after correction for multiple testing.

| **Rank** | **GO category** | **ID** | **Total probes in pathway** | **dDMPs in pathway** | **Odds ratio** | **p value** | **Bonferroni-corrected p value** |
| --- | --- | --- | --- | --- | --- | --- | --- |
| 1 | visual perception | GO:0007601 | 3551 | 7 | 7.55 | 6.91E-05 | 0.80 |
| 2 | sensory perception of light stimulus | GO:0050953 | 3619 | 7 | 7.41 | 7.77E-05 | 0.90 |
| 3 | response to methotrexate | GO:0031427 | 54 | 2 | 134.68 | 1.19E-04 | 1.00 |
| 4 | cell-cell junction assembly | GO:0007043 | 1722 | 5 | 10.91 | 1.36E-04 | 1.00 |
| 5 | cell-cell junction organization | GO:0045216 | 3833 | 6 | 5.91 | 7.79E-04 | 1.00 |
| 6 | 1-phosphatidylinositol binding | GO:0005545 | 637 | 3 | 17.32 | 8.22E-04 | 1.00 |
| 7 | cell junction assembly | GO:0034329 | 3988 | 6 | 5.68 | 9.55E-04 | 1.00 |
| 8 | photoreceptor cell development | GO:0042461 | 674 | 3 | 16.36 | 9.66E-04 | 1.00 |
| 9 | quaternary ammonium group transmembrane transporter activity | GO:0015651 | 159 | 2 | 45.81 | 9.79E-04 | 1.00 |
| 10 | lipid binding | GO:0008289 | 11167 | 10 | 3.48 | 1.19E-03 | 1.00 |
| 11 | amino acid transport | GO:0006865 | 2818 | 5 | 6.64 | 1.25E-03 | 1.00 |
| 12 | sodium:dicarboxylate symporter activity | GO:0017153 | 192 | 2 | 37.91 | 1.41E-03 | 1.00 |
| 13 | structural constituent of eye lens | GO:0005212 | 221 | 2 | 32.91 | 1.86E-03 | 1.00 |
| 14 | phospholipid binding | GO:0005543 | 6221 | 7 | 4.27 | 1.92E-03 | 1.00 |
| 15 | cell junction organization | GO:0034330 | 4581 | 6 | 4.94 | 1.92E-03 | 1.00 |
| 16 | inorganic anion exchanger activity | GO:0005452 | 226 | 2 | 32.17 | 1.94E-03 | 1.00 |
| 17 | photoreceptor cell differentiation | GO:0046530 | 879 | 3 | 12.54 | 2.05E-03 | 1.00 |
| 18 | acidic amino acid transmembrane transporter activity | GO:0015172 | 238 | 2 | 30.56 | 2.15E-03 | 1.00 |
| 19 | L-glutamate transmembrane transporter activity | GO:0005313 | 238 | 2 | 30.56 | 2.15E-03 | 1.00 |
| 20 | photoreceptor disc membrane | GO:0097381 | 248 | 2 | 29.32 | 2.33E-03 | 1.00 |

**SUPPLEMENTARY TABLE 16 - Top-twenty gene ontology pathways significantly enriched in the lightcyan WGCNA module,** which is strongly associated with sex in the developing fetal brain.

| **Rank** | **GO category** | **ID** | **Total probes in pathway** | **dDMPs in pathway** | **Odds ratio** | **p value** | **Bonferroni-corrected p value** |
| --- | --- | --- | --- | --- | --- | --- | --- |
| 1 | negative regulation of histone H3-K36 methylation | GO:0000415 | 122 | 12 | 63.83 | 7.34E-18 | 8.46E-14 |
| 2 | negative regulation of tooth mineralization | GO:0070171 | 126 | 12 | 61.80 | 1.05E-17 | 1.21E-13 |
| 3 | regulation of histone H3-K36 methylation | GO:0000414 | 153 | 12 | 50.83 | 9.41E-17 | 1.09E-12 |
| 4 | specification of axis polarity | GO:0065001 | 163 | 12 | 47.72 | 1.93E-16 | 2.23E-12 |
| 5 | negative regulation of histone H3-K4 methylation | GO:0051572 | 169 | 12 | 46.03 | 2.90E-16 | 3.34E-12 |
| 6 | regulation of tooth mineralization | GO:0070170 | 257 | 13 | 32.87 | 1.07E-15 | 1.23E-11 |
| 7 | chloride channel activity | GO:0005254 | 1164 | 20 | 11.31 | 1.17E-14 | 1.35E-10 |
| 8 | anion channel activity | GO:0005253 | 1219 | 20 | 10.80 | 2.70E-14 | 3.11E-10 |
| 9 | anion transmembrane transporter activity | GO:0008509 | 4411 | 34 | 5.18 | 9.31E-14 | 1.07E-09 |
| 10 | negative regulation of bone mineralization | GO:0030502 | 331 | 12 | 23.51 | 5.90E-13 | 6.80E-09 |
| 11 | negative regulation of biomineral tissue development | GO:0070168 | 335 | 12 | 23.23 | 6.76E-13 | 7.79E-09 |
| 12 | negative regulation of histone methylation | GO:0031061 | 454 | 13 | 18.61 | 1.14E-12 | 1.31E-08 |
| 13 | histone H2A monoubiquitination | GO:0035518 | 356 | 12 | 21.86 | 1.34E-12 | 1.55E-08 |
| 14 | chloride transmembrane transporter activity | GO:0015108 | 1629 | 20 | 8.07 | 4.72E-12 | 5.44E-08 |
| 15 | negative regulation of histone modification | GO:0031057 | 784 | 15 | 12.47 | 5.64E-12 | 6.50E-08 |
| 16 | histone H2A ubiquitination | GO:0033522 | 419 | 12 | 18.57 | 8.40E-12 | 9.69E-08 |
| 17 | regulation of histone H3-K4 methylation | GO:0051569 | 466 | 12 | 16.69 | 2.76E-11 | 3.18E-07 |
| 18 | voltage-gated chloride channel activity | GO:0005247 | 318 | 10 | 20.30 | 1.93E-10 | 2.23E-06 |
| 19 | histone monoubiquitination | GO:0010390 | 563 | 12 | 13.82 | 2.27E-10 | 2.62E-06 |
| 20 | negative regulation of chromosome organization | GO:2001251 | 1046 | 15 | 9.34 | 2.82E-10 | 3.25E-06 |

**SUPPLEMENTARY TABLE 17 - Top-twenty gene ontology pathways significantly enriched in the black WGCNA module,** which is strongly associated with sex in the developing fetal brain.

| **Rank** | **GO category** | **ID** | **Total probes in pathway** | **dDMPs in pathway** | **Odds ratio** | **p value** | **Bonferroni-corrected p value** |
| --- | --- | --- | --- | --- | --- | --- | --- |
| 1 | neuronal ribonucleoprotein granule | GO:0071598 | 55 | 30 | 51.81 | 1.82E-37 | 2.10E-33 |
| 2 | establishment or maintenance of neuroblast polarity | GO:0045196 | 67 | 31 | 43.97 | 6.30E-37 | 7.26E-33 |
| 3 | asymmetric neuroblast division | GO:0055059 | 67 | 31 | 43.97 | 6.30E-37 | 7.26E-33 |
| 4 | establishment of neuroblast polarity | GO:0045200 | 67 | 31 | 43.97 | 6.30E-37 | 7.26E-33 |
| 5 | negative regulation of microtubule depolymerization | GO:0007026 | 478 | 53 | 10.61 | 2.87E-34 | 3.31E-30 |
| 6 | regulation of microtubule depolymerization | GO:0031114 | 511 | 53 | 9.92 | 5.89E-33 | 6.79E-29 |
| 7 | MAPK cascade | GO:0000165 | 4234 | 140 | 3.21 | 5.24E-30 | 6.04E-26 |
| 8 | protein polymerization | GO:0051258 | 1077 | 68 | 6.06 | 1.24E-29 | 1.43E-25 |
| 9 | mitochondrial inner membrane | GO:0005743 | 5358 | 160 | 2.91 | 1.91E-29 | 2.20E-25 |
| 10 | signal transduction by phosphorylation | GO:0023014 | 4914 | 151 | 2.99 | 4.52E-29 | 5.21E-25 |
| 11 | embryonic olfactory bulb interneuron precursor migration | GO:0021831 | 61 | 25 | 38.91 | 5.16E-29 | 5.95E-25 |
| 12 | microtubule polymerization | GO:0046785 | 143 | 31 | 20.61 | 1.88E-28 | 2.17E-24 |
| 13 | histone H4-K16 acetylation | GO:0043984 | 263 | 37 | 13.40 | 1.11E-27 | 1.28E-23 |
| 14 | globus pallidus development | GO:0021759 | 71 | 25 | 33.43 | 1.14E-27 | 1.31E-23 |
| 15 | organelle inner membrane | GO:0019866 | 6062 | 168 | 2.70 | 2.07E-27 | 2.39E-23 |
| 16 | mitochondrial membrane | GO:0031966 | 8166 | 194 | 2.32 | 8.44E-24 | 9.73E-20 |
| 17 | guanylate kinase activity | GO:0004385 | 318 | 35 | 10.47 | 4.61E-23 | 5.32E-19 |
| 18 | sex determination | GO:0007530 | 630 | 46 | 6.97 | 6.46E-23 | 7.45E-19 |
| 19 | negative regulation of collateral sprouting | GO:0048671 | 231 | 31 | 12.76 | 6.78E-23 | 7.82E-19 |
| 20 | phosphotransferase activity | GO:0016776 | 718 | 48 | 6.38 | 2.45E-22 | 2.83E-18 |

**SUPPLEMENTARY TABLE 18 – Fetal brain dDMPs contained within high-confidence regions identified in a recent large GWAS analysis of schizophrenia.** Chromosomal coordinates correspond to human genome build Feb. 2009 (GRCh37/hg19). Regions containing no 450K array probes were removed. Bonferroni correction was used to define significance based on the total number of probes within the schizophrenia-associated genomic regions (n = 4864). Variant and pmin identify the most significant SNP/indel within each region and the corresponding p value reported by the PGC (Schizophrenia Working Group of the Psychiatric Genomics Consortium 2014).

| **Chr** | **Start** | **Stop** | **Variant** | **pmin** | **Total probes** | **Number**  **p < 1.03E-5 (%)** | **Probes passing p < 1.25E-7 threshold** |
| --- | --- | --- | --- | --- | --- | --- | --- |
| 1 | 2372401 | 2402501 | rs4648845 | 8.70E-10 | 41 | 7 (17.07) | cg00030604 cg14129430 cg15029248 cg15548859 |
| 1 | 8411184 | 8638984 | Chr1_8424984_D | 1.17E-09 | 61 | 10 (16.39) | cg01771201 cg04096723 cg08653904 cg11080552 cg15148088 cg15701149 cg25722041 |
| 1 | 30412551 | 30437271 | rs1498232 | 2.86E-09 | 1 | 0 (0.00) |  |
| 1 | 44029384 | 44128084 | rs11210892 | 3.39E-10 | 57 | 6 (10.53) | cg13235366 cg19710386 |
| 1 | 97792625 | 98559084 | rs1702294 | 3.36E-19 | 45 | 0 (0.00) |  |
| 1 | 149998890 | 150242490 | rs140505938 | 4.49E-10 | 81 | 5 (6.17) | cg02340851 cg20791007 |
| 1 | 177247821 | 177300821 | rs6670165 | 4.45E-08 | 1 | 1 (100.00) | cg18766795 |
| 1 | 207912183 | 208024083 | rs7523273 | 4.47E-08 | 49 | 7 (14.29) | cg20817822 cg24536146 |
| 1 | 243503719 | 244002945 | rs77149735 | 3.73E-09 | 49 | 13 (26.53) | cg00295780 cg01833436 cg02528319 cg15962538 cg20059832 cg20686125 cg20995689 cg22950111 cg24455383 |
| 2 | 57943593 | 58502192 | rs11682175 | 1.47E-11 | 31 | 1 (3.23) |  |
| 2 | 72357335 | 72368185 | rs3768644 | 7.39E-09 | 10 | 3 (30.00) |  |
| 2 | 149390778 | 149520178 | Chr2_146436222_I | 1.81E-09 | 20 | 1 (5.00) | cg26872780 |
| 2 | 162798555 | 162910255 | rs2909457 | 4.62E-08 | 4 | 2 (50.00) | cg06581825 cg20427486 |
| 2 | 185601420 | 185785420 | rs11693094 | 1.53E-12 | 1 | 0 (0.00) |  |
| 2 | 198148577 | 198835577 | rs6434928 | 2.06E-11 | 147 | 1 (0.68) | cg25880834 |
| 2 | 200161422 | 200309252 | rs6704641 | 8.33E-09 | 8 | 1 (12.50) | cg04554576 |
| 2 | 200715237 | 200848037 | Chr2_200825237_I | 5.65E-14 | 31 | 0 (0.00) |  |
| 2 | 225334096 | 225467796 | rs11685299 | 1.12E-08 | 16 | 2 (12.50) | cg01474011 |
| 2 | 233559301 | 233753501 | rs6704768 | 2.32E-12 | 43 | 3 (6.98) | cg20388707 |
| 3 | 2532786 | 2561686 | rs17194490 | 2.69E-11 | 5 | 1 (20.00) |  |
| 3 | 17221366 | 17888266 | rs4330281 | 4.64E-09 | 16 | 2 (12.50) | cg07654588 cg19131667 |
| 3 | 36843183 | 36945783 | rs75968099 | 1.05E-13 | 8 | 0 (0.00) |  |
| 3 | 52541105 | 52903405 | rs2535627 | 4.26E-11 | 144 | 10 (6.94) | cg03143060 cg07757611 cg09354317 cg12941836 cg18182844 cg18591801 cg21547371 |
| 3 | 63792650 | 64004050 | rs832187 | 1.43E-08 | 44 | 4 (9.09) | cg10253967 cg24027263 cg25490800 |
| 3 | 135807405 | 136615405 | rs7432375 | 7.26E-11 | 94 | 7 (7.45) | cg00658740 cg02858132 cg08031689 cg10727673 cg20519665 cg25964728 |
| 3 | 180588843 | 181205585 | Chr3_180594593_I | 1.30E-11 | 30 | 3 (10.00) | cg14914887 |
| 4 | 23366403 | 23443403 | rs215411 | 3.06E-08 | 1 | 0 (0.00) |  |
| 4 | 103146888 | 103198090 | rs35518360 | 7.98E-15 | 2 | 0 (0.00) |  |
| 4 | 170357552 | 170646052 | rs10520163 | 1.47E-09 | 26 | 0 (0.00) |  |
| 5 | 60499143 | 60843543 | rs4391122 | 1.10E-14 | 45 | 9 (20.00) | cg07213616 cg19602446 cg20565082 cg27085488 cg27132966 cg27302175 |
| 5 | 88581331 | 88854331 | rs16867576 | 4.61E-09 | 1 | 0 (0.00) |  |
| 5 | 109030036 | 109209066 | rs4388249 | 3.05E-08 | 7 | 2 (28.57) | cg23500208 |
| 5 | 137598121 | 137948092 | rs3849046 | 4.67E-09 | 136 | 5 (3.68) | cg08519191 cg23057943 cg26941476 |
| 5 | 140023664 | 140222664 | Chr5_140143664_I | 4.85E-08 | 134 | 0 (0.00) |  |
| 5 | 151941104 | 152797656 | rs111294930 | 1.06E-10 | 12 | 5 (41.67) | cg03702477 cg09055743 cg20740845 |
| 5 | 153671057 | 153688217 | rs11740474 | 3.15E-08 | 1 | 0 (0.00) |  |
| 6 | 28303247 | 28712247 | rs115329265 | 3.48E-31 | 353 | 8 (2.27) | cg04645150 cg07734514 cg24114014 |
| 6 | 84279922 | 84407274 | Chr6_84280274_D | 8.15E-10 | 1 | 0 (0.00) |  |
| 7 | 1896096 | 2190096 | Chr7_2025096_I | 8.20E-15 | 491 | 101 (20.57) | cg00183100 cg00647879 cg00749118 cg00963171 cg01258793 cg01843768 cg01952989 cg02568153 cg02827175 cg03604067 cg04170282 cg04396112 cg04481102 cg04555379 cg04848693 cg04898039 cg05342467 cg05775771 cg05863683 cg05894015 cg06789500 cg07065756 cg07556911 cg07610468 cg07690127 cg08465311 cg08607108 cg08972190 cg09075743 cg09174162 cg09776772 cg10445988 cg10950524 cg11001739 cg11870042 cg12492273 cg13504346 cg13571479 cg13868473 cg14108894 cg14638384 cg14972721 cg15089567 cg15686782 cg15896696 cg16344173 cg16772998 cg16993108 cg17018896 cg17545141 cg17551891 cg18268948 cg18483265 cg19266014 cg19419389 cg19629631 cg22460466 cg23375948 cg23378565 cg23393892 cg23873406 cg24077588 cg24247786 cg24295561 cg24787238 cg25397636 cg26014580 cg27109748 cg27238358 cg27305769 |
| 7 | 24619494 | 24832094 | Chr7_24747494_D | 2.85E-08 | 32 | 8 (25.00) | cg01733570 cg09333471 cg19706795 cg22804000 cg25577212 cg25723149 cg27436603 |
| 7 | 86403226 | 86459326 | rs12704290 | 3.33E-10 | 5 | 1 (20.00) | cg04062190 |
| 7 | 104598064 | 105063064 | rs6466055 | 1.13E-09 | 73 | 5 (6.85) | cg02170577 cg21261709 |
| 7 | 110843815 | 111205915 | rs211829 | 3.71E-08 | 13 | 2 (15.38) | cg19380793 |
| 7 | 137039644 | 137085244 | rs7801375 | 4.42E-08 | 1 | 1 (100.00) | cg11019771 |
| 8 | 4177794 | 4192544 | rs10503253 | 1.06E-08 | 3 | 0 (0.00) |  |
| 8 | 27412627 | 27453627 | rs73229090 | 2.10E-08 | 15 | 2 (13.33) |  |
| 8 | 60475469 | 60954469 | rs6984242 | 5.97E-09 | 5 | 1 (20.00) | cg15654892 |
| 8 | 89340626 | 89753626 | rs7819570 | 1.22E-08 | 11 | 2 (18.18) |  |
| 8 | 143309503 | 143330533 | rs4129585 | 1.74E-15 | 6 | 0 (0.00) |  |
| 9 | 84630941 | 84813641 | rs11139497 | 3.61E-09 | 2 | 2 (100.00) | cg13925809 |
| 10 | 18681005 | 18770105 | rs7893279 | 1.97E-12 | 7 | 1 (14.29) | cg03587042 |
| 10 | 104423800 | 105165583 | rs11191419 | 6.20E-19 | 199 | 16 (8.04) | cg02721701 cg05885577 cg08757448 cg10632966 cg10992686 cg14015502 cg16236779 cg24435571 |
| 11 | 46342943 | 46751213 | Chr11_46350213_D | 1.26E-11 | 186 | 19 (10.22) | cg05450701 cg06266097 cg09802018 cg18568067 cg20199333 cg21756806 cg26473110 cg27137887 |
| 11 | 57386294 | 57682294 | rs9420 | 2.24E-09 | 116 | 1 (0.86) | cg06434864 |
| 11 | 109285471 | 109610071 | rs12421382 | 3.70E-08 | 22 | 0 (0.00) |  |
| 11 | 113317794 | 113423994 | rs2514218 | 2.75E-11 | 21 | 2 (9.52) | cg20424531 |
| 11 | 124610007 | 124620147 | rs55661361 | 2.80E-12 | 13 | 0 (0.00) |  |
| 11 | 130714610 | 130749330 | rs10791097 | 1.09E-12 | 7 | 0 (0.00) |  |
| 11 | 133808069 | 133852969 | rs75059851 | 3.87E-11 | 30 | 5 (16.67) | cg01152015 cg06610125 cg22821947 |
| 12 | 2321860 | 2523731 | rs2007044 | 3.22E-18 | 40 | 4 (10.00) | cg16303353 cg20495738 cg23369234 |
| 12 | 29905265 | 29940365 | rs679087 | 3.91E-08 | 14 | 2 (14.29) | cg13318543 cg26479868 |
| 12 | 57428314 | 57682971 | rs12826178 | 2.02E-12 | 235 | 15 (6.38) | cg02138198 cg05441768 cg06715885 cg09827071 cg16451431 cg16612926 cg17651972 cg23047693 |
| 12 | 92243186 | 92258286 | rs4240748 | 4.59E-08 | 1 | 0 (0.00) |  |
| 12 | 103559855 | 103616655 | rs10860964 | 4.84E-08 | 1 | 0 (0.00) |  |
| 12 | 123448113 | 123909113 | rs2851447 | 1.86E-14 | 205 | 22 (10.73) | cg02948656 cg03061518 cg04844995 cg05399718 cg07949141 cg14844236 cg16389456 cg16427983 cg20181887 cg24335116 cg25630910 cg26804387 cg27422311 |
| 14 | 99707919 | 99719219 | rs2693698 | 4.80E-09 | 10 | 2 (20.00) | cg07440398 cg10515131 |
| 14 | 103996234 | 104184834 | rs12887734 | 1.36E-13 | 116 | 10 (8.62) | cg13247935 cg13511324 cg14228272 cg18597188 cg23335576 |
| 15 | 40566759 | 40602237 | rs56205728 | 4.18E-09 | 38 | 10 (26.32) | cg02240622 cg05547778 cg10316525 cg14076977 cg16567056 cg20079298 |
| 15 | 61831663 | 61909663 | rs12903146 | 3.38E-10 | 3 | 0 (0.00) |  |
| 15 | 70573672 | 70628872 | rs12148337 | 1.79E-08 | 3 | 1 (33.33) |  |
| 15 | 78803032 | 78926732 | rs8042374 | 2.44E-13 | 49 | 4 (8.16) | cg17108064 cg19696491 cg22563815 |
| 15 | 84661161 | 85153461 | rs950169 | 1.62E-11 | 28 | 1 (3.57) |  |
| 15 | 91416560 | 91429040 | rs4702 | 8.30E-14 | 23 | 3 (13.04) |  |
| 16 | 9875519 | 9970219 | rs9922678 | 1.28E-08 | 4 | 1 (25.00) |  |
| 16 | 29924377 | 30144877 | rs12691307 | 4.55E-11 | 191 | 20 (10.47) | cg01298987 cg03303433 cg03890691 cg04502620 cg05806717 cg08464513 cg08683263 cg14370448 cg16348385 cg27106909 cg27151362 |
| 16 | 67709340 | 68311340 | rs8044995 | 1.51E-08 | 286 | 20 (6.99) | cg01068601 cg01756421 cg02215611 cg02506867 cg03061352 cg04202511 cg06378129 cg06774893 cg08082862 cg08281590 cg08571821 cg08958168 cg09756419 cg16485385 cg19899008 cg23656386 |
| 17 | 2095899 | 2220799 | rs4523957 | 2.86E-10 | 21 | 4 (19.05) | cg17648080 cg20205704 |
| 17 | 17722402 | 18030202 | rs8082590 | 1.77E-08 | 121 | 19 (15.70) | cg03376089 cg04324276 cg04901053 cg08129017 cg14611258 cg16460860 cg16928487 cg17793286 cg19142141 cg21275283 cg22315164 cg25999891 cg26588076 cg27407935 |
| 18 | 52747686 | 53200117 | rs9636107 | 3.34E-12 | 8 | 3 (37.50) | cg15171911 |
| 18 | 53453389 | 53804154 | rs72934570 | 1.97E-11 | 1 | 0 (0.00) |  |
| 19 | 19374022 | 19658022 | rs2905426 | 3.63E-10 | 141 | 5 (3.55) | cg00477287 cg00741634 cg16629695 |
| 19 | 50067499 | 50135399 | rs56873913 | 4.69E-08 | 39 | 2 (5.13) | cg21961766 |
| 20 | 37361494 | 37485994 | rs6065094 | 1.46E-11 | 35 | 5 (14.29) | cg13691108 cg16119522 cg25098585 cg26680502 cg27377213 |
| 20 | 48114136 | 48131649 | rs7267348 | 4.56E-08 | 1 | 0 (0.00) |  |
| 22 | 39975317 | 40016817 | Chr22_39987017_D | 4.73E-11 | 1 | 1 (100.00) |  |
| 22 | 41408556 | 41675156 | rs9607782 | 2.07E-11 | 67 | 2 (2.99) | cg19833679 cg22970173 |
| 22 | 42315744 | 42689414 | rs6002655 | 1.71E-09 | 153 | 22 (14.38) | cg07175554 cg08057985 cg08650629 cg09569869 cg09667013 cg14532519 cg14697880 cg16047117 cg18266530 cg19026547 cg24977886 cg25343227 cg25459323 cg26469058 |
| X | 5916533 | 6032733 | rs12845396 | 2.21E-08 | 1 | 0 (0.00) |  |
| X | 21193266 | 21570266 | rs1378559 | 1.61E-12 | 14 | 0 (0.00) |  |
| ***Total*** | ***-*** | ***-*** |  |  | ***4864*** | ***466 (9.58)*** | ***-*** |

**SUPPLEMENTARY TABLE 19 - Fetal brain dDMPs amongst probes annotated to autism risk genes.** Chromosomal coordinates correspond to human genome build Feb. 2009 (GRCh37/hg19). Genes containing no informative 450K array probes were removed. Bonferroni correction was used to define significance based on the total number of probes within the schizophrenia-associated genomic regions (n = 709).

| **Gene** | **Chr** | **Start** | **Stop** | **Total probes** | **Number**  **p < 7.05E-5 (%)** | **Probes passing p < 1.25E-7 threshold** |
| --- | --- | --- | --- | --- | --- | --- |
| *All gene-annotated probes* | - | - | - | 352,191 | 38,845 (11.03) | - |
| *NTNG1* | 1 | 107681350 | 108023482 | 33 | 2 (6.06) | cg05802545 |
| *POGZ* | 1 | 151375993 | 151433350 | 17 | 1 (5.88) | cg15197893 |
| *NRXN1* | 2 | 50163732 | 51260739 | 59 | 11 (18.64) | cg04595505 cg15912010 cg18773591 cg23851515 cg08066024 cg14329860 |
| *TBR1* | 2 | 162271412 | 162281498 | 33 | 6 (18.18) | cg14557487 cg12520549 cg12757011 cg10942521 |
| *SCN2A* | 2 | 166095825 | 166236760 | 7 | 1 (14.29) |  |
| *CUL3* | 2 | 225363361 | 225450859 | 15 | 4 (26.67) | cg01474011 |
| *CNTN4* | 3 | 2140977 | 3097738 | 34 | 5 (14.71) | cg21613099 cg08144588 |
| *ANK2* | 4 | 113738613 | 114304349 | 48 | 7 (14.58) | cg18219250 cg04522984 |
| *MET* | 7 | 116311496 | 116438184 | 21 | 4 (19.05) | cg24197567 |
| *CNTNAP2* | 7 | 145812842 | 148117075 | 63 | 8 (12.70) | cg18216587 cg19260567 cg26942930 |
| *PTEN* | 10 | 89621740 | 89725510 | 58 | 0 (0.00) |  |
| *GRIN2B* | 12 | 13715497 | 14134486 | 33 | 7 (21.21) | cg14434398 cg09977288 |
| *GRIP1* | 12 | 66742565 | 67073760 | 20 | 2 (10.00) | cg10865238 |
| *CHD8* | 14 | 21853470 | 21905982 | 18 | 0 (0.00) |  |
| *CACNA1H* | 16 | 1202015 | 1271741 | 151 | 21 (13.91) | cg01643563 cg00675160 cg14976500 cg06518618 cg05031011 cg04983933 cg07990390 cg10863145 cg27201940 cg01162915 cg00840270 cg00300794 cg06008356 cg02605306 cg09463706 |
| *KATNAL2* | 18 | 44525710 | 44627440 | 26 | 3 (11.54) | cg06573143 |
| *SHANK1* | 19 | 51165207 | 51221605 | 47 | 14 (29.79) | cg17826344 cg01427575 |
| *DYRK1A* | 21 | 38738374 | 38886664 | 17 | 3 (17.65) | cg21505925 cg25518092 |
| *PTCHD1* | X | 23351681 | 23413343 | 9 | 0 (0.00) |  |
| ***Total*** | ***-*** | ***-*** | ***-*** | ***709*** | ***99 (13.96)*** |  |

**SUPPLEMENTARY TABLE 20 – Human fetal brain samples included in this study.** M = male; F = female. Age is given in days post-conception as determined by Carnegie staging for embryonic samples defined as ≤56 days post-conception, and foot and knee to heel length measurements in the case of fetal samples defined as ≥57 days post-conception. HDBR = the Human Developmental Biology Resource (HDBR) fetal tissue bank; MRC = the MRC London Brainbank for Neurodegenerative Diseases at the Institute of Psychiatry, King’s College London (see Methods). No additional phenotypic or demographic data was available for these samples. The age and sex distribution of the samples used in this study is shown in **Figure 1a**.

| **Sample ID** | **Sex** | **Source** | **Age** |  | **n** | **Sex** | **Source** | **Age** |
| --- | --- | --- | --- | --- | --- | --- | --- | --- |
| 1 | M | HDBR | 23 |  | 91 | M | HDBR | 90 |
| 2 | F | HDBR | 37 |  | 92 | F | HDBR | 91 |
| 3 | F | HDBR | 44 |  | 93 | F | HDBR | 91 |
| 4 | M | HDBR | 46 |  | 94 | M | HDBR | 91 |
| 5 | F | HDBR | 47 |  | 95 | M | HDBR | 91 |
| 6 | M | HDBR | 49 |  | 96 | M | HDBR | 92 |
| 7 | M | HDBR | 53 |  | 97 | M | HDBR | 92 |
| 8 | F | HDBR | 56 |  | 98 | F | HDBR | 92 |
| 9 | M | HDBR | 56 |  | 99 | F | HDBR | 92 |
| 10 | F | HDBR | 57 |  | 100 | F | HDBR | 93 |
| 11 | M | HDBR | 58 |  | 101 | M | HDBR | 93 |
| 12 | M | HDBR | 59 |  | 102 | M | HDBR | 93 |
| 13 | M | HDBR | 59 |  | 103 | F | HDBR | 93 |
| 14 | F | HDBR | 61 |  | 104 | M | HDBR | 94 |
| 15 | M | HDBR | 62 |  | 105 | F | HDBR | 94 |
| 16 | F | HDBR | 63 |  | 106 | F | HDBR | 94 |
| 17 | M | HDBR | 63 |  | 107 | M | HDBR | 94 |
| 18 | F | HDBR | 64 |  | 108 | F | HDBR | 94 |
| 19 | M | HDBR | 64 |  | 109 | M | HDBR | 94 |
| 20 | M | HDBR | 64 |  | 110 | M | HDBR | 95 |
| 21 | M | HDBR | 65 |  | 111 | F | HDBR | 96 |
| 22 | F | HDBR | 65 |  | 112 | M | HDBR | 96 |
| 23 | F | HDBR | 67 |  | 113 | M | HDBR | 97 |
| 24 | M | HDBR | 68 |  | 114 | F | HDBR | 97 |
| 25 | F | HDBR | 68 |  | 115 | F | HDBR | 97 |
| 26 | M | HDBR | 69 |  | 116 | M | HDBR | 97 |
| 27 | M | HDBR | 70 |  | 117 | M | HDBR | 98 |
| 28 | F | HDBR | 70 |  | 118 | M | HDBR | 98 |
| 29 | F | HDBR | 71 |  | 119 | F | HDBR | 98 |
| 30 | F | HDBR | 71 |  | 120 | F | HDBR | 99 |
| 31 | M | HDBR | 71 |  | 121 | F | HDBR | 99 |
| 32 | M | HDBR | 71 |  | 122 | M | HDBR | 99 |
| 33 | M | HDBR | 72 |  | 123 | M | HDBR | 99 |
| 34 | M | HDBR | 72 |  | 124 | F | HDBR | 100 |
| 35 | F | HDBR | 73 |  | 125 | F | HDBR | 101 |
| 36 | M | HDBR | 73 |  | 126 | F | HDBR | 101 |
| 37 | M | HDBR | 74 |  | 127 | F | HDBR | 102 |
| 38 | F | HDBR | 74 |  | 128 | M | HDBR | 103 |
| 39 | F | HDBR | 74 |  | 129 | F | HDBR | 104 |
| 40 | M | HDBR | 75 |  | 130 | M | HDBR | 104 |
| 41 | F | HDBR | 75 |  | 131 | M | HDBR | 104 |
| 42 | M | HDBR | 76 |  | 132 | M | HDBR | 105 |
| 43 | F | HDBR | 76 |  | 133 | M | HDBR | 106 |
| 44 | M | HDBR | 76 |  | 134 | M | HDBR | 106 |
| 45 | F | HDBR | 77 |  | 135 | F | HDBR | 107 |
| 46 | M | HDBR | 77 |  | 136 | M | HDBR | 108 |
| 47 | M | HDBR | 77 |  | 137 | M | HDBR | 109 |
| 48 | F | HDBR | 77 |  | 138 | M | HDBR | 110 |
| 49 | M | HDBR | 78 |  | 139 | F | HDBR | 110 |
| 50 | M | HDBR | 78 |  | 140 | M | HDBR | 110 |
| 51 | F | HDBR | 78 |  | 141 | M | HDBR | 111 |
| 52 | M | HDBR | 79 |  | 142 | M | HDBR | 111 |
| 53 | M | HDBR | 79 |  | 143 | F | MRC | 112 |
| 54 | F | HDBR | 79 |  | 144 | M | HDBR | 112 |
| 55 | F | HDBR | 79 |  | 145 | M | HDBR | 112 |
| 56 | F | HDBR | 80 |  | 146 | M | HDBR | 113 |
| 57 | F | HDBR | 80 |  | 147 | F | HDBR | 113 |
| 58 | M | HDBR | 80 |  | 148 | M | HDBR | 114 |
| 59 | M | HDBR | 80 |  | 149 | F | HDBR | 114 |
| 60 | M | HDBR | 81 |  | 150 | F | HDBR | 115 |
| 61 | M | HDBR | 81 |  | 151 | M | HDBR | 116 |
| 62 | F | HDBR | 81 |  | 152 | M | HDBR | 117 |
| 63 | F | HDBR | 81 |  | 153 | M | HDBR | 118 |
| 64 | M | HDBR | 82 |  | 154 | F | HDBR | 118 |
| 65 | M | HDBR | 82 |  | 155 | M | MRC | 119 |
| 66 | M | HDBR | 83 |  | 156 | M | HDBR | 119 |
| 67 | F | HDBR | 83 |  | 157 | F | HDBR | 120 |
| 68 | M | HDBR | 83 |  | 158 | M | HDBR | 120 |
| 69 | M | HDBR | 84 |  | 159 | M | HDBR | 120 |
| 70 | F | HDBR | 84 |  | 160 | M | HDBR | 122 |
| 71 | M | HDBR | 84 |  | 161 | M | HDBR | 122 |
| 72 | F | HDBR | 84 |  | 162 | M | HDBR | 123 |
| 73 | M | HDBR | 85 |  | 163 | F | HDBR | 124 |
| 74 | M | HDBR | 85 |  | 164 | F | HDBR | 124 |
| 75 | M | HDBR | 86 |  | 165 | F | HDBR | 125 |
| 76 | F | HDBR | 86 |  | 166 | F | HDBR | 126 |
| 77 | M | HDBR | 86 |  | 167 | F | HDBR | 127 |
| 78 | F | HDBR | 86 |  | 168 | F | HDBR | 128 |
| 79 | M | HDBR | 87 |  | 169 | F | HDBR | 129 |
| 80 | F | HDBR | 87 |  | 170 | M | MRC | 133 |
| 81 | M | HDBR | 87 |  | 171 | M | MRC | 133 |
| 82 | F | HDBR | 87 |  | 172 | F | MRC | 140 |
| 83 | F | HDBR | 88 |  | 173 | F | MRC | 140 |
| 84 | F | HDBR | 88 |  | 174 | M | MRC | 140 |
| 85 | M | HDBR | 88 |  | 175 | M | HDBR | 153 |
| 86 | M | HDBR | 88 |  | 176 | F | MRC | 154 |
| 87 | F | HDBR | 89 |  | 177 | F | MRC | 161 |
| 88 | M | HDBR | 89 |  | 178 | M | HDBR | 169 |
| 89 | F | HDBR | 90 |  | 179 | M | HDBR | 184 |

**SUPPLEMENTARY TABLE 21 - Cross-reactive and SNP-affected probes excluded during quality control of DNA methylation data.**

| **Probe type** | **Criteria** | **(n)** | **Source** |
| --- | --- | --- | --- |
| SNP probes (rs probes) | All | 64 | Illumina annotation |
| Non-specific cg probes | All | 29,233 | Chen *et al*., 2013 |
| Non-specific ch probes | All | 1,736 | Chen *et al*., 2013 |
| Non-specific cg probes | All | 41,937 | Price *et al*., 2013 |
| SNP probes | Within ≤ 10bp of SBE site, and AF ≥ 0.05 | 14,090 | Chen *et al*., 2013 |
| SNP probes | Within ≤ 10bp of SBE site, and AF ≥ 0.05 | 17,944 | Illumina annotation |
| **Total unique probes removed:** | | **58,559** | |
